# Selective Transfer Semihydrogenation of Alkynes Catalyzed by an Iron PCP Pincer Alkyl Complex

**DOI:** 10.1021/acscatal.3c04156

**Published:** 2023-10-17

**Authors:** Heiko Schratzberger, Berthold Stöger, Luis F. Veiros, Karl Kirchner

**Affiliations:** † Institute of Applied Synthetic Chemistry, TU Wien, Getreidemarkt 9/163-AC, A-1060 Wien, Austria; ‡ X-Ray Center, TU Wien, Getreidemarkt 9/163, A-1060 Wien, Austria; $ Centro de Química Estrutural, Institute of Molecular Sciences, Departamento de Engenharia Química, Instituto Superior Técnico, Universidade de Lisboa, Av. Rovisco Pais, 1049 001 Lisboa, Portugal

**Keywords:** iron, alkyl complexes, alkynes, semihydrogenation, pincer complexes, DFT calculations

## Abstract

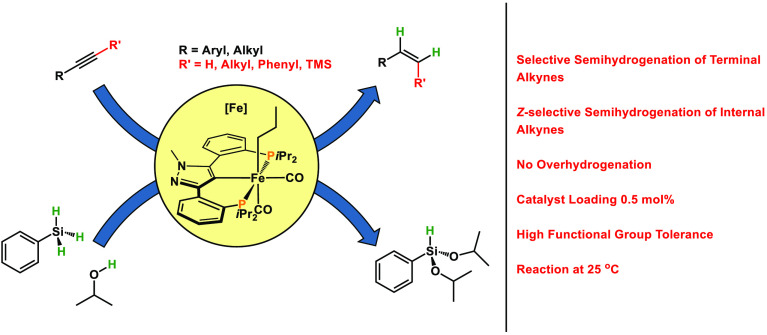

Two bench-stable Fe(II) alkyl complexes [Fe(κ^3^PCP-PCP-*i*Pr)(CO)_2_(R)] (R = CH_2_CH_2_CH_3_, CH_3_) were obtained by the treatment of [Fe(κ^3^PCP-PCP-*i*Pr)(CO)_2_(H)] with NaNH_2_ and subsequent addition of CH_3_CH_2_CH_2_Br and CH_3_I, respectively. The reaction proceeds via the anionic Fe(0) intermediate Na[Fe(κ^3^PCP-PCP-*i*Pr)(CO)_2_]. The catalytic performance of both alkyl complexes was investigated for the transfer hydrogenation of terminal and internal alkynes utilizing PhSiH_3_ and *i*PrOH as a hydrogen source. Precatalyst activation is initiated by migration of the alkyl ligand to the carbonyl C atom of an adjacent CO ligand. In agreement with previous findings, the rate of alkyl migration follows the order *n*Pr > Me. Accordingly, [Fe(κ^3^PCP-PCP-*i*Pr)(CO)_2_(CH_2_CH_2_CH_3_)] is the more active catalyst. The reaction takes place at 25 °C with a catalyst loading of 0.5 mol%. There was no overhydrogenation, and in the case of internal alkynes, exclusively, *Z*-alkenes are formed. The implemented protocol tolerates a variety of electron-donating and electron-withdrawing functional groups including halides, nitriles, unprotected amines, and heterocycles. Mechanistic investigations including deuterium labeling studies and DFT calculations were undertaken to provide a reasonable reaction mechanism.

## Introduction

The preparation of alkenes from alkynes via selective semihydrogenation is an attractive process for the synthesis of pharmaceuticals and bulk and fine chemicals in the chemical industry as well as in research.^1^ A challenging task of this reaction is the control of the chemo- and stereoselectivity, since the reduction of C≡C triple bonds to alkenes may lead to the formation of (*E*)- or (*Z*)-alkenes as well as saturated hydrocarbons.^2^ Consequently, the development of new and selective homogeneous semihydrogenation catalysts for reduction to either (*Z*)- or (*E*)-alkenes that prevent isomerization and over-reduction would still be of great importance.^3^

**Scheme 1:**
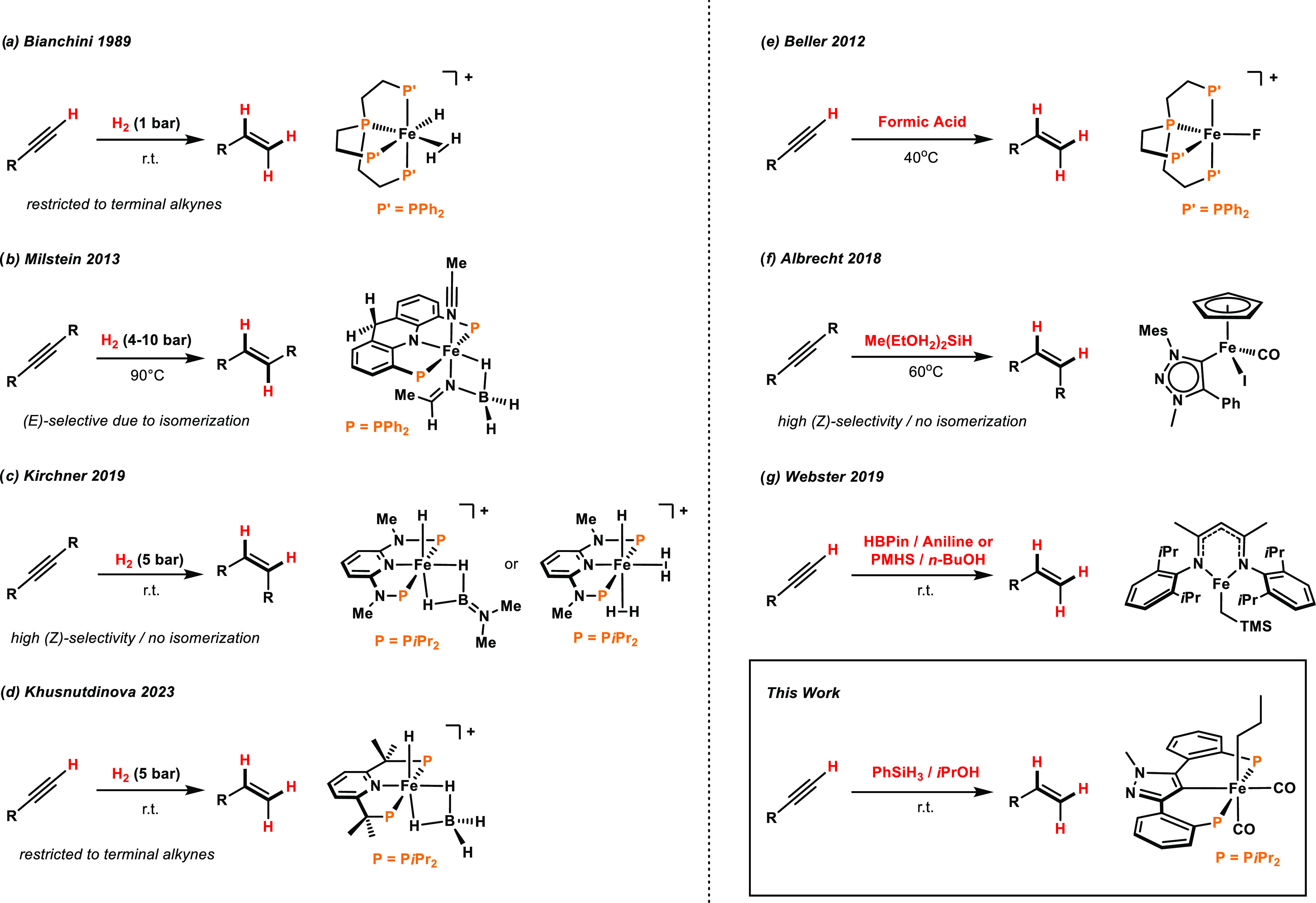
Semihydrogenation (Left) and Transfer Semihydrogenation (Right) of Alkynes Catalyzed by Well-Defined Iron Complexes

Within this context, we are interested in the design of active homogeneous catalysts based on first-row transition metals. Obvious advantages such as their low price and high abundance are complemented by their intrinsic properties that may provide unprecedented reactivities and selectivities in catalytic transformations.^4^ Iron has evolved to a particularly promising candidate in this regard in recent years.^5^ In the field of alkyne reductions, only a few iron complexes were reported that operate via direct hydrogenation or transfer hydrogenation procedures. As of yet, only a few examples are currently known that can promote this transformation, employing hydrogen gas as the reductant (Scheme 1). In 1989, Bianchini et al. discovered the semihydrogenation of terminal alkynes catalyzed by the nonclassical polyhydride [Fe(PP_3_)(H)(η^2^-H_2_)]^+^.^6^ Milstein and co-workers reported on a novel acridine-based pincer type complex that bears an imino borohydride coligand that was found to reduce internal alkynes selectively to the respective (*E*)-olefins.^7^ We described that the bench-stable cationic bis(σ–B-H) aminoborane complex [Fe(PNP^NMe^-*i*Pr)(H)(η^2^-H_2_B = NMe_2_)]^+^ as well as the cationic complex [Fe(PNP^NMe^-*i*Pr)(H)(η^2^-H_2_)_2_]^+^ efficiently catalyzes the semihydrogenation of internal alkynes, 1,3-diynes and 1,3-enynes.^8^ More recently, Khusnutdinova and co-workers reported on an efficient semihydrogenation of terminal alkynes with H_2_, catalyzed by a modified, tetramethylated PNP pincer Fe hydride complex.^9^

With respect to iron-catalyzed transfer semihydrogenations of alkynes,^1a^ [Fe_2_(CO)_9_] in the presence of monodentate phosphines was able to reduce internal aryl-substituted alkynes with (EtO)_3_SiH with moderate to good *Z*/*E* selectivities.^10^ The group of Plietker showed that [Fe(PPh_3_)_2_(CO)(NO)(H)] catalyzes the transfer semihydrogenations of diaryl-alkynes where the *Z*/*E* ratio was dependent on the silane used.^11^ Beller and co-workers reported the semihydrogenation of terminal alkynes utilizing formic acid as a hydrogen source with an in situ generated cationic Fe(II) tetraphos catalyst (Scheme 1).^12^ Albrecht and co-workers used 1,2,3-triazolylidene and IMes iron piano-stool complexes for the catalytic semihydrogenation of alkynes by using silanes as reducing agents. Aromatic terminal alkynes were converted to styrenes without over-reduction to ethylbenzene derivatives. Internal aryl alkynes afford cis-alkenes with excellent *Z*-selectivity.^13^ The combination of boranes with amines or, alternatively, silanes such as PMHS (poly(methylhydrosiloxane)) and alcohols as hydrogen donors mediated by an Fe(II) β-diketiminate alkyl system was reported by the group of Webster.^14^ This system, depending on the stoichiometry, allowed for full reduction as well as semihydrogenation but limited to 50% conversion.

We recently described the application of a well-defined Mn(I)-alkyl complex^15^ as a catalyst for the hydrogenation of nitriles, ketones, CO_2_, alkenes, and alkynes as well as the dehydrogenative silylation of alkenes and hydroboration of alkynes.^16,17^ We took advantage of the fact that Mn(I)-alkyl carbonyl complexes undergo migratory insertion of the nucleophilic alkyl ligand into the polarized CO moiety, yielding a coordinatively unsaturated acyl complex, which is capable of activating weakly polar E–H bonds (E = –H, –C≡C-R, –BR_2_, –SiR_3_). The rate of alkyl migration follows the order *n*Pr > Et > Me as already shown by Moss and co-workers some years ago.^18^

**Scheme 2:**
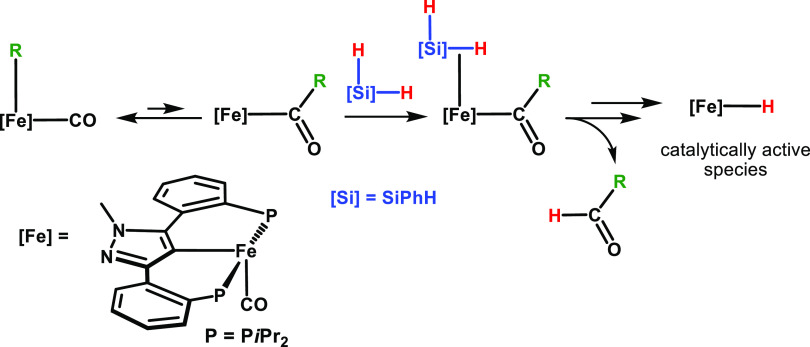
Formation of a Coordinatively Unsaturated Fe(II) Hydride Species via Alkyl Migration and Deprotonation of the Entering Ligand

In this article, we apply this concept to iron alkyl PCP complexes. Accordingly, the catalytic process is initiated by migratory insertion of a CO ligand into the Fe–alkyl bond to yield acyl intermediates, which react with silanes in the presence of alcohols to form the 16e^–^ Fe(II) hydride catalysts (Scheme 2). We describe here an iron-catalyzed selective transfer semihydrogenation of terminal alkynes and *Z*-selective transfer semihydrogenation of internal alkynes utilizing a combination of phenylsilane and isopropanol as the hydrogen source under mild conditions. It has to be noted that catalytic applications of iron PCP complexes are scarce.^19^ Rare examples are the hydrosilylation of ketones and aldehydes,^20^ the dehydrogenation of ammonia–borane,^21^ and the dehydrogenative borylation of styrene.^22^

## Results and Discussion

**Scheme 3:**
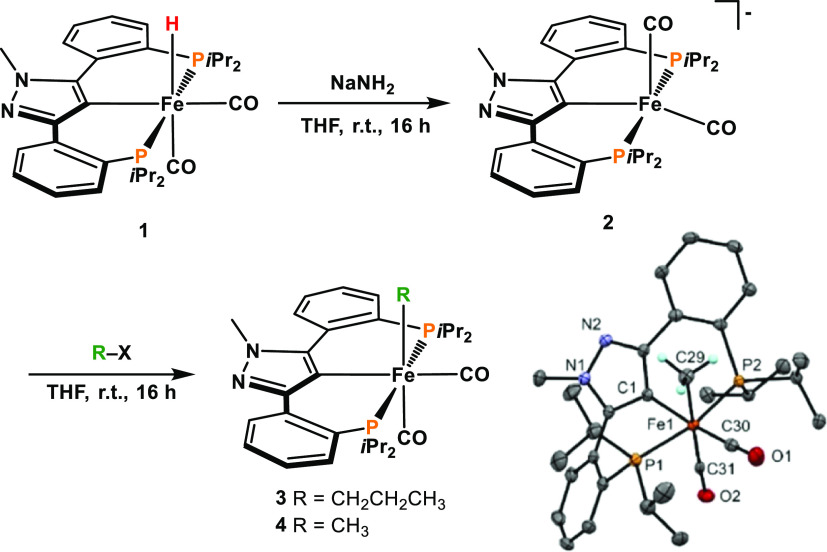
Synthesis of [Fe(κ^3^PCP-PCP-*i*Pr)(CO)_2_(R)] (3, 4) and Structural View of 4·1.5 C_6_H_6_ Showing 50% Ellipsoids (Most H Atoms and Solvent Omitted for Clarity) Selected bond lengths (Å) and bond angles (deg): Fe1–C1 2.020(2), Fe1–C29 2.110(3), Fe1–P1 2.2686(7), Fe1–P2 2.2487(7), Fe1–C30 1.759(2), Fe1–C31 1.779(4), C1–Fe1–C30 170.8(1), P1–Fe1–P2 166.81(3), and C29–Fe1–C31 177.6(2).

The new Fe(II) alkyl complexes [Fe(κ^3^PCP-PCP-*i*Pr)(CO)_2_(R)] (R = CH_2_CH_2_CH_3_ (**3**), CH_3_ (**4**)) were obtained by treatment of [Fe(κ^3^PCP-PCP-*i*Pr)(CO)_2_(H)] (**1**)^23^ with NaNH_2_ (3 equiv) and subsequent addition of CH_3_CH_2_CH_2_Br and BrCH_3_I, respectively, in 63 and 71% isolated yields (Scheme 3). The reaction proceeds *via* the anionic Fe(0) intermediate [Fe(κ^3^PCP-PCP-*i*Pr)(CO)_2_]^−^ (**2**) that could also be isolated in 91% yield. Complex **2** gives rise to ν_CO_ vibrations at 1774 and 1708 cm^–1^, which are significantly shifted to lower wavenumbers lower than the respective resonances in **1** being 1976 and 1918 cm^–1^.^23^ These values are comparable with the anionic pyrrole-based pincer complex [Fe(PN_pyrr_P)(CO)_2_]^−^ reported by Tonzetich and co-workers.^24^ Both alkyl complexes are bench-stable for several weeks in the presence of air. It has to be noted that **3** and **4** form two isomers depending on the position of the *N*-methyl group of the pyrazole moiety in relation to the alkyl ligands (see the Supporting Information, Figure S1). Due to the large six-membered metallacycles, the rotation around the axis C_ipso_-Fe-CO is slow on the NMR timescale, resulting in the two conformers being detectable in NMR spectra. A similar behavior was observed with the *N*-methylated congener [Fe(κ^3^PCP-PCP^Me^-*i*Pr)(CO)_2_(Cl)]BF_4_ reported previously.^23^ DFT calculations reveal that the energy difference between these isomers is merely 0.5 kcal/mol. The IR spectra of **3** and **4** exhibit two strong C–O vibrations at 1969 and 1907 and 1970 and 1908 cm^–1^, respectively, being characteristic of a *cis*-geometry of the carbonyl ligands. They were fully characterized by ^1^H, ^13^C{^1^H}, and ^31^P{^1^H} NMR and IR spectroscopy and high-resolution mass spectrometry. In addition, the molecular structure of **4** was determined by X-ray crystallography. A structural view is depicted in Scheme 3 with selected bond distances and angles given in the caption.

The catalytic performance of complexes **3** and **4** was investigated for the transfer hydrogenation of alkynes with silanes and *i*PrOH as a hydrogen source. In order to establish the best reaction conditions, phenylacetylene was chosen as a model substrate. Selected optimization experiments are depicted in Table 1.

**Table 1:**
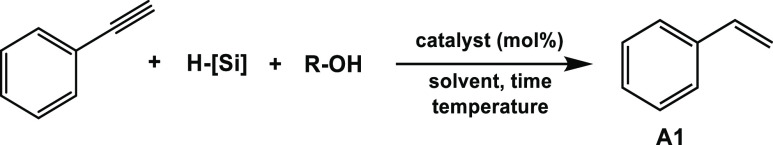
Optimization Reactions for the Catalytic Semihydrogenation of Phenylacetylene^a^

entry	catalyst (mol %)	H-[Si]	alcohol	solvent	*T* (°C)	conversion (%)^d^
1	**3** (1)	PhSiH_3_	*i*PrOH	benzene	80	>99
2	**3** (1)	Ph_2_SiH_2_	*i*PrOH	benzene	80	>99
3	**3** (1)	(MeO)_3_SiH	MeOH	benzene	80	55
4	**3** (1)	PMHS	*i*PrOH	neat	80	27
5	**3** (0.5)	PhSiH_3_	*i*PrOH	benzene	80	>99
6	**3** (0.25)	PhSiH_3_	*i*PrOH	benzene	80	54
7	**3** (0.5)	PhSiH_3_	*i*PrOH	benzene	60	46
8	**3** (0.5)	PhSiH_3_	*i*PrOH	THF	70	>99
9	**4** (0.5)	PhSiH_3_	*i*PrOH	THF	70	93
10	**3** (0.5)	PhSiH_3_	*i*PrOH	THF	60	>99
11	**3** (0.5)	PhSiH_3_	*i*PrOH	THF	20	75
**12**^b^	**3 (0.5)**	**PhSiH**_**3**_	***i*****PrOH**	**THF**	**20**	**> 99**
13^b^	**4** (0.5)	PhSiH_3_	*i*PrOH	THF	20	29
14^c^	**3** (0.5)	PhSiH_3_	*i*PrOH	THF	20	60

a Reaction conditions: 0.6 mL of anhydrous solvent, 0.161 mmol phenylacetylene, 1 equiv silane, and alcohol, 24 h.

b 1.25 equiv alcohol.

c 1.50 equiv alcohol.

d Conversion determined via GC-MS.

Various silanes were evaluated in order to optimize catalytic activity and also to investigate the influence of the silane on the product selectivity in the presence of *i*PrOH (1 equiv with respect to silane). In the absence of alcohol, mixtures of semihydrogenated and hydrosilylated products were obtained. Moreover, attempts to utilize hydrogen gas as a reducing agent were unsuccessful.

At 80 °C, in benzene with 1 mol % **3**, PhSiH_3_ and Ph_2_SiH_2_ afforded the highest activity (Table 1, entries 1 and 2), while (MeO)_3_SiH and PMHS (in MeOH) gave significantly poorer conversions (Table 1, entries 3 and 4). Cheap and easy-to-handle PMHS decreased the activity by about a factor of 4. In all cases, overhydrogenation did not take place. There was also no evidence of alkyne hydrosilylation under these conditions. Further lowering of the catalyst loading to 0.25 mol % resulted in 54% conversion (Table 1, entries 6). Decreasing the reaction temperature from 80 to 60 °C afforded **A1** in 46% conversion (Table 1, entry 7). The temperature could be significantly lowered when switching from benzene to THF as a solvent. With a catalyst loading of 0.5 mol % and PhSiH_3_, quantitative formation of **A1** was achieved at 60 °C (Table 1, entry 5), while at 20 °C, **A1** was still obtained in 75% yield (Table 1, entry 11). Quantitative formation of **A1** was observed at 20 °C when the amount of *i*PrOH was increased to 1.25 equiv. (Table 1, entry 12). Upon further increasing the amount of *i*PrOH to 1.5 equiv., a drop in conversion to 60% was observed (Table 1 entry 14). The catalytic activity of precatalyst **4** was also investigated, which turned out to be less efficient under similar reaction conditions (Table 1, entries 9 and 13) which is in agreement with previous findings that the rate of alkyl migration follows the order *n*Pr > Me.^15,18^

If the catalytic reaction is performed in the presence of PMe_3_, no transfer semihydrogenation reaction took place, indicating that PMe_3_ coordinates to the Fe(II) center, also blocking the vacant site for incoming substrates. Moreover, in the absence of the catalyst, no reaction took place. The homogeneity of the reaction was confirmed by addition of one drop of mercury, where no decrease of reactivity and selectivity was observed.

Having established the best reaction conditions, the applicability of catalyst **3** is demonstrated in the selective hydrogenation of various terminal and internal alkynes. These results are shown in Table 2. In general, the alkyne transfer semihydrogenation is accompanied by the formation of [(1-methylethoxy)silyl]benzene, [bis(1-methylethoxy)silyl]benzene, and dihydrogen. A range of 4-substituted ethynylbenzenes were hydrogenated under the optimized reaction conditions. Both electron-donating groups such as OMe, Me, *t*Bu, and NH_2_ and electron-withdrawing substituents such as F, Cl, CN, acyl, and NO_2_ are compatible with the semihydrogenation protocol (Table 2, **A1**–**A12**) and showed good selectivity to give substituted styrenes in 44–99% yields. 4-Ethynylbenzonitrile yielded only 44% of **A7**, while 3-ethynylphenol did not react to the desired alkyne **A11** at all. It must be mentioned that dehalogenation or nitrile reduction was not observed.

**Table 2:**
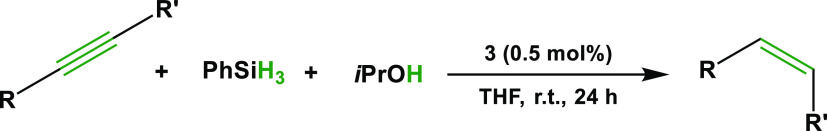

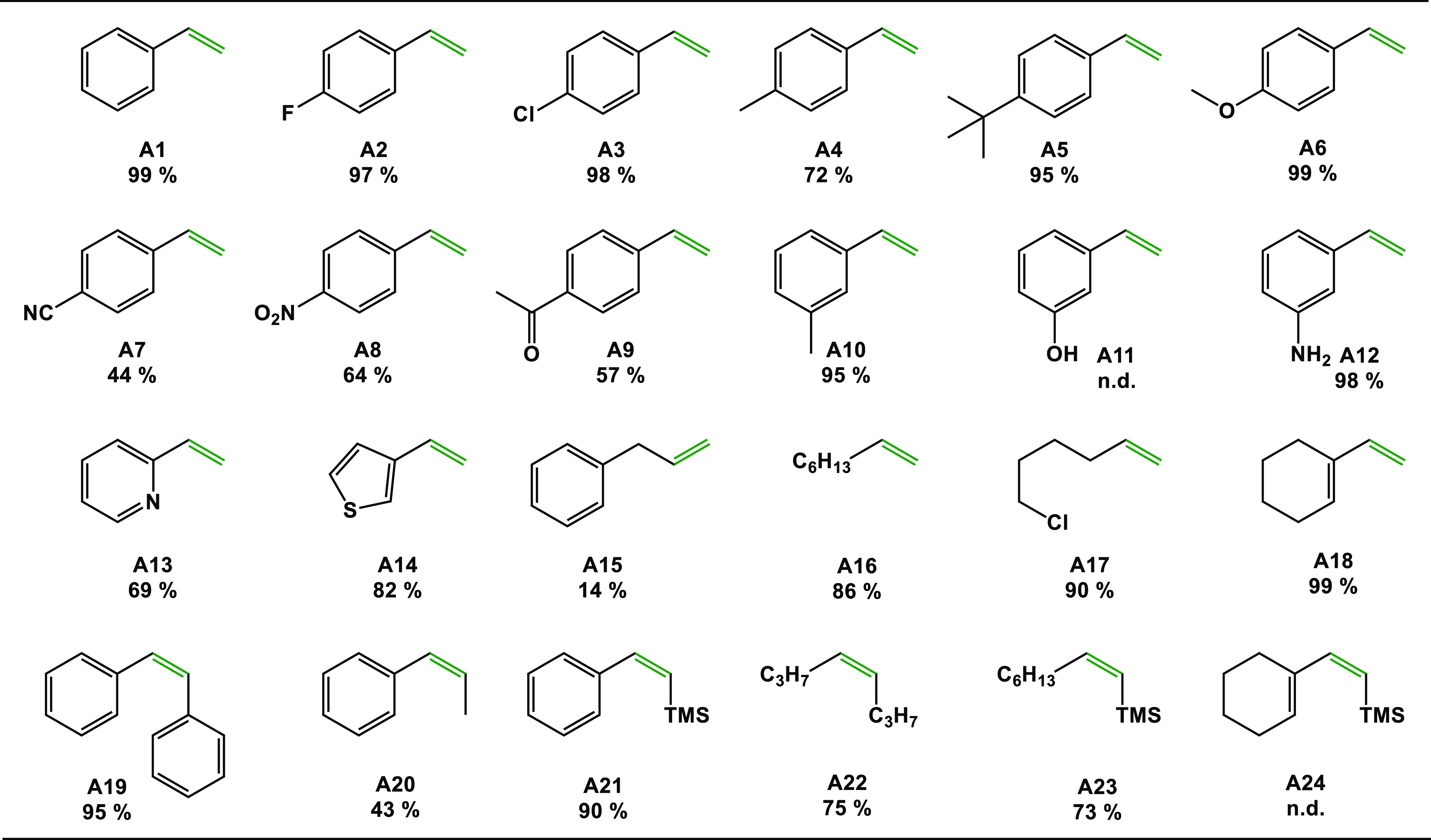
Semihydrogenation of Various Alkynes Catalyzed by [Fe(κ^3^PCP-PCP-*i*Pr)(CO)_2_(CH_2_CH_2_CH_3_)] (3) with PhSiH_3_ and *i*PrOH as a Hydrogen Source^a^,^b^,^c^

a Reaction conditions for terminal alkynes: alkyne (0.161 mmol, 1 equiv), PhSiH_3_ (0.161 mmol, 1 equiv), *i*PrOH (0.201 mmol, 1.25 equiv), **3** (0.5 mol %), THF-*d*_8_ (0.6 mL), 25 °C, and 24 h,

b Reaction conditions for internal alkynes: alkyne (0.161 mmol, 1 equiv), PhSiH_3_ (0.161 mmol, 1 equiv), *i*PrOH (0.242 mmol, 1.50 equiv), **3** (0.5 mol %), THF-*d*_8_ (0.6 mL), 25 °C, and 24 h.

c Yield determined by ^1^H NMR spectroscopy using mesitylene as an internal standard.

In addition, we tested the heteroaromatic alkynes 2-ethynylpyridine and 3-ethynylthiophene, which were converted to the corresponding alkenes **A13** and **A14**, respectively, in 69 and 82% yields. Several aliphatic terminal alkynes were also tested, giving, with the exception of 3-phenylprop-1-yne (**A15**), good to excellent yields (**A16**–**A18**). As a challenging example, 1-ethynylcyclohexene with its conjugated double and triple bonds was selectively transformed into its diene product **A18**.

Aryl–aryl, aryl–alkyl, and alky–alkyl substituted alkynes were also investigated. Substrates bearing alkyl groups tend to be more challenging in selective semihydrogenations due to over-reduction and isomerization. Under the given reaction conditions, diphenylacetylene and various nonactivated alkynes bearing alkyl substituents afforded the corresponding *Z*-alkenes in good to excellent yields (**A19**–**A23**). Unfortunately, 1-[2-(trimethylsilyl)ethynyl]cyclohexene was not reduced to the desired alkene 1-[(1*Z*)-2-(trimethylsilyl)ethenyl]cyclohexene (**A24**).

**Scheme 4:**
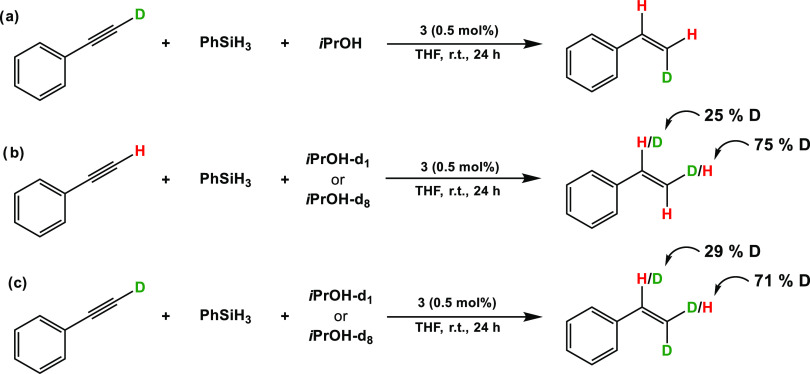
Deuterium Labeling Studies of the Transfer Semihydrogenation of Phenylacetylene with PhSiH_3_ and *i*PrOH

To gain further insights in the reaction mechanism, deuterium labeling experiments were carried out. The regio- and stereoselectivity of deuterium incorporation was established via ^2^H NMR spectroscopy. With phenylacetylene-d_1_ as a substrate, styrene is formed with the deuterium being at the terminal *cis*-position with respect to the phenyl ring (Scheme 4a). This clearly shows that the *syn*-addition of both hydrogen atoms took place. If *i*PrOH-*d*_1_ or *i*PrOH-*d*_8_ was used, 75% of deuterium ended up at the terminal carbon and 25% of deuterium was incorporated at the internal carbon of styrene (Scheme 4b). Performing the reaction with both phenylacetylene-d_1_ and *i*PrOH-*d*_1_ or *i*PrOH-*d*_8_ reveals again *syn*-H/D addition since the deuterium originating from phenylacetylene-d_1_ is again located in the *cis*-position to the phenyl ring (Scheme 4c).

**Figure 1:**
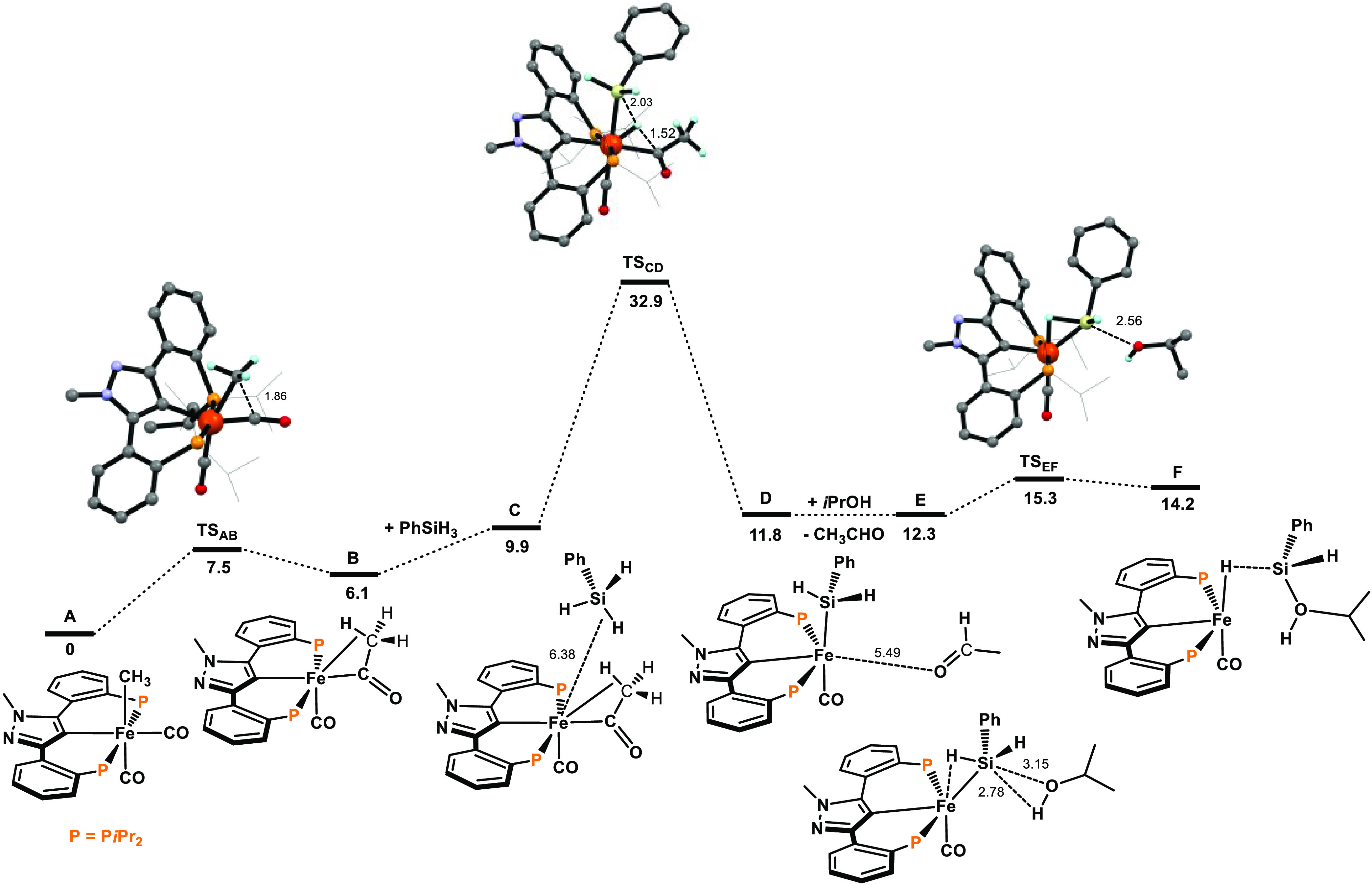
Free energy profile for the precatalyst activation with PhSiH_3_ and *i*PrOH. Free energies (kcal/mol) are referred to [Fe(κ^3^PCP-PCP-*i*Pr)(CO)_2_(CH_3_)] (A).

**Figure 2:**
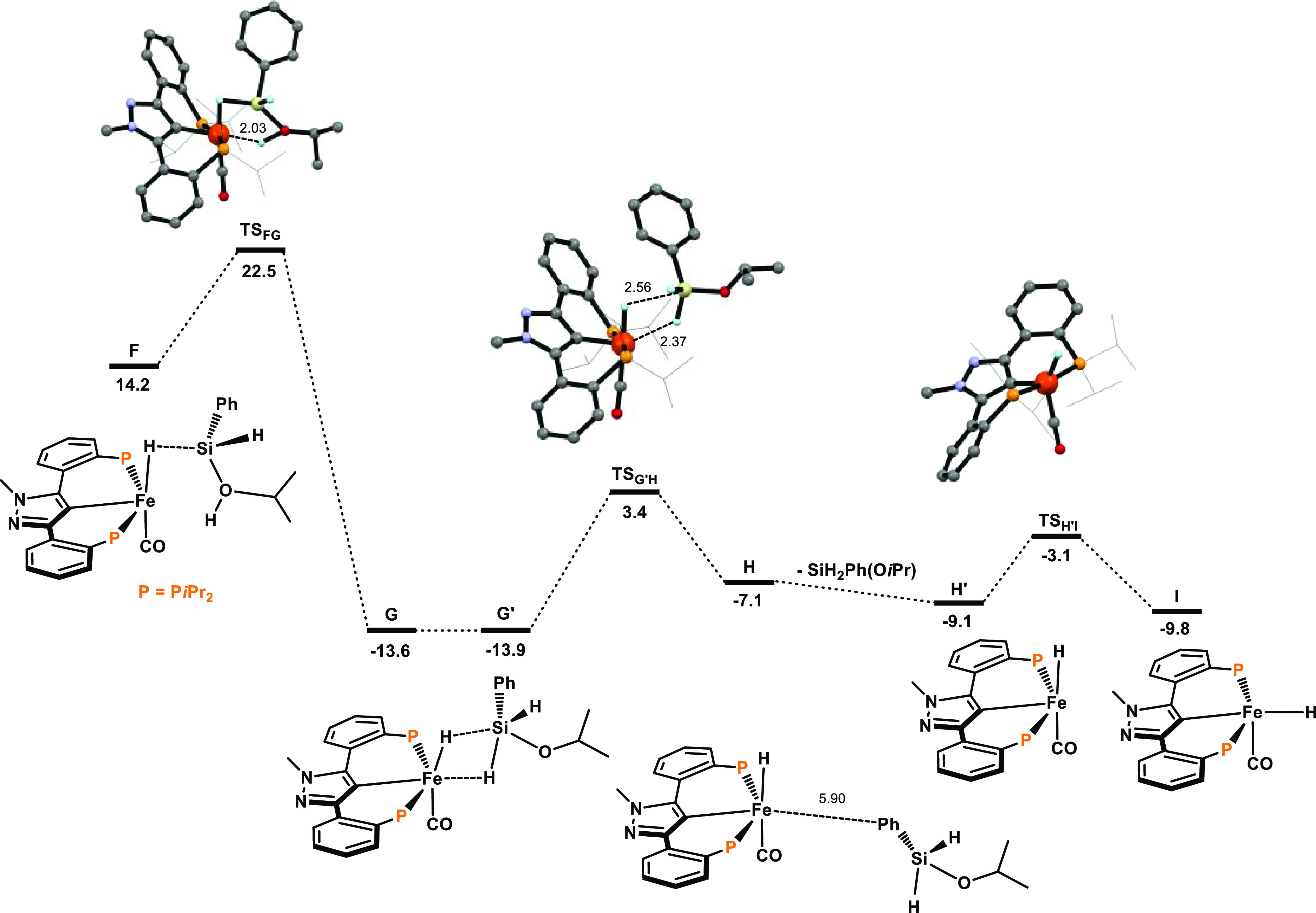
Free energy profile for the precatalyst activation with PhSiH_3_ and *i*PrOH. Free energies (kcal/mol) are referred to [Fe(κ^3^PCP-PCP-*i*Pr)(CO)_2_(CH_3_)] (A).

**Figure 3:**
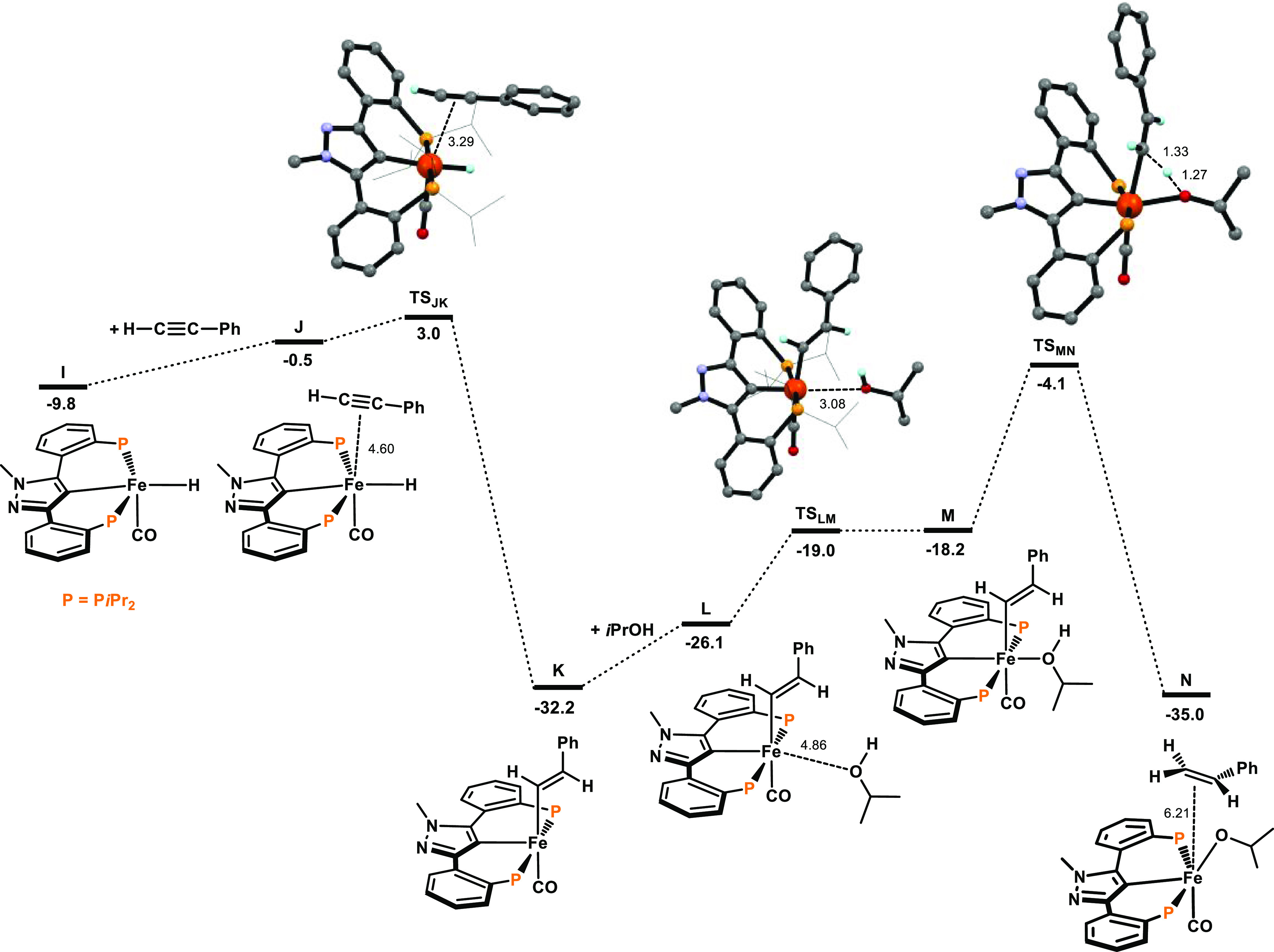
Free energy profile for the transfer semihydrogenation of phenylacetylene. Free energies (kcal/mol) are referred to [Fe(κ^3^PCP-PCP-*i*Pr)(CO)_2_(CH_3_)] (A).

**Figure 4:**
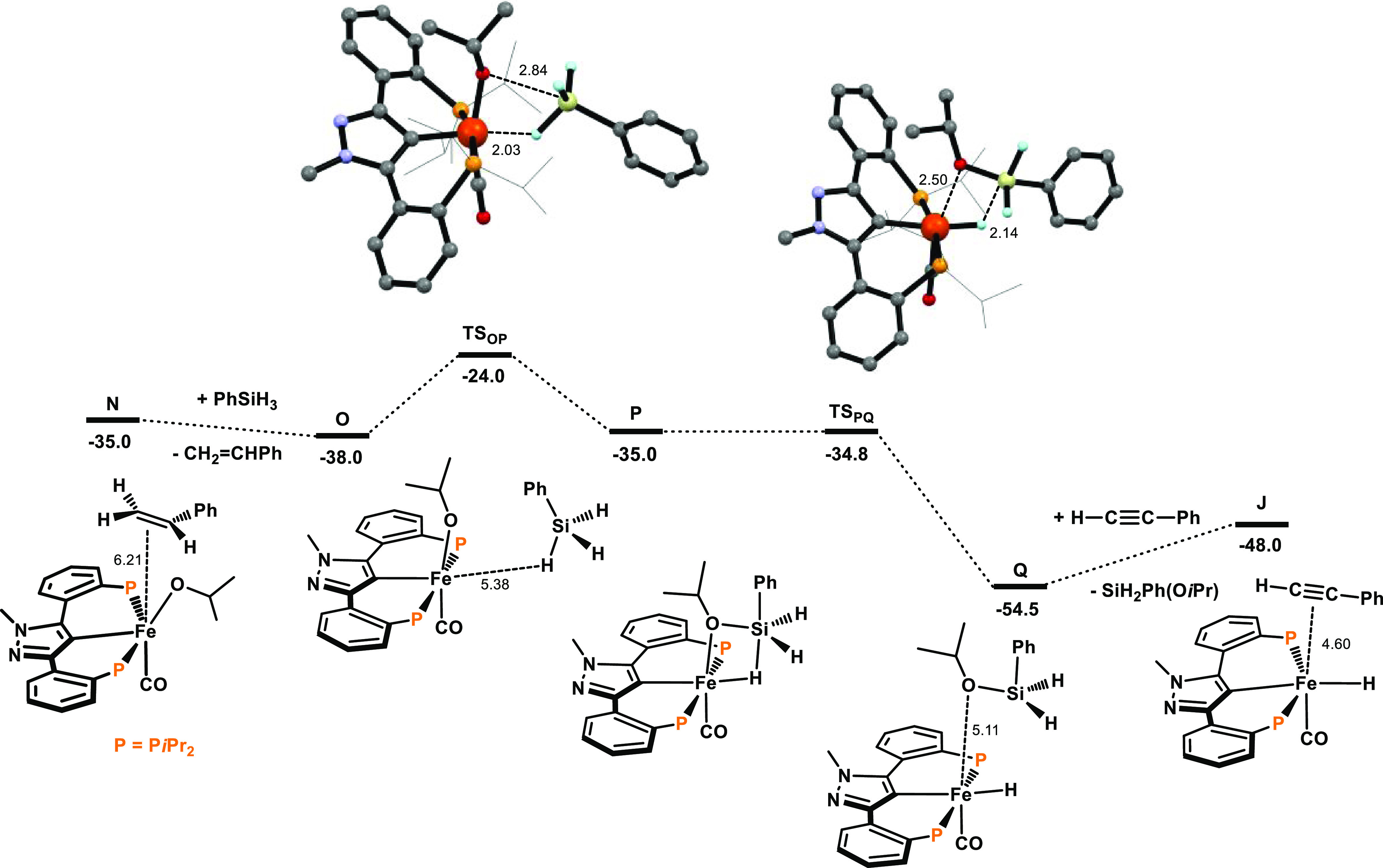
Free energy profile for the transfer semihydrogenation of phenylacetylene. Free energies (kcal/mol) are referred to [Fe(κ^3^PCP-PCP-*i*Pr)(CO)_2_(CH_3_)] (A).

**Scheme 5:**
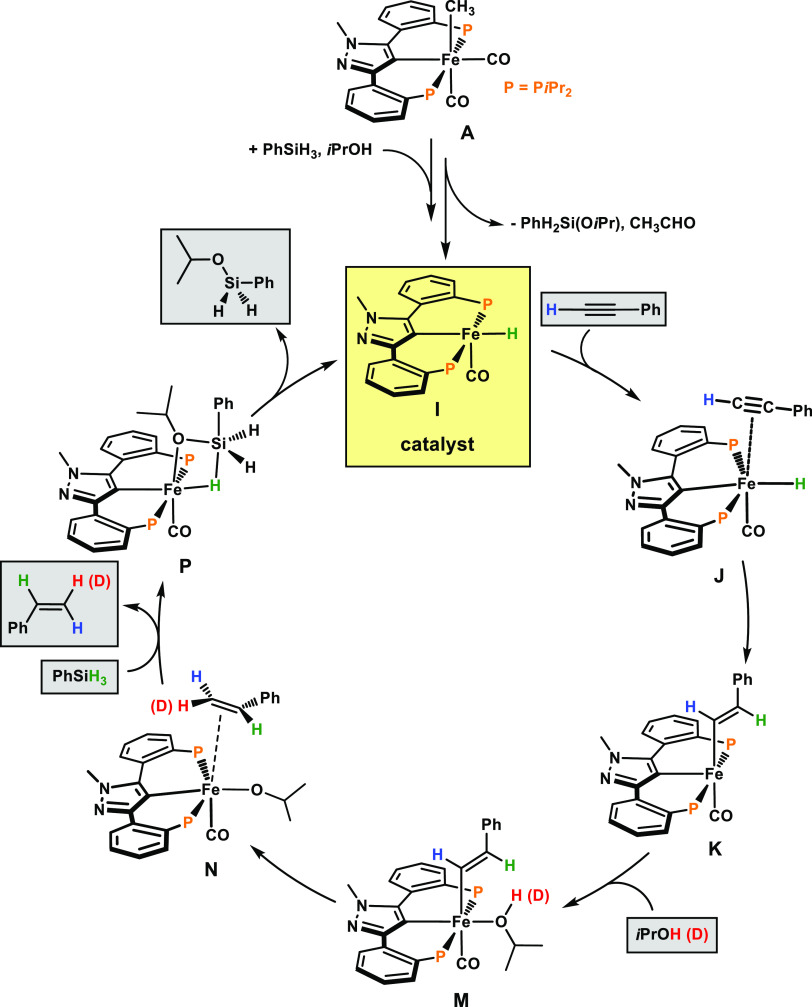
Simplified Catalytic Cycle for the Transfer Semihydrogenation of Phenylacetylene

A plausible catalytic cycle based on experimental data and DFT calculations^25^ with diphenylacetylene (**A1**) as a model substrate, *i*PrOH and PhSiH_3_ as a hydrogen source, and [Fe(κ^3^PCP-PCP-*i*Pr)(CO)_2_(CH_3_)] (**4**, **A** in the calculations) as a precatalyst could be established. The resulting free energy profiles for the activation of the precatalyst are represented in Figures 1 and 2. The free energy profiles for the transfer semihydrogenation are depicted in Figures 3 and 4. A simplified catalytic cycle (only key intermediates are shown) is depicted in Scheme 5.

Precatalyst activation is initiated by migration of the alkyl ligand in complex **A** to the carbonyl C-atom of an adjacent CO ligand (Figure 1). This occurs in an easy step with a barrier of only 8 kcal/mol, producing intermediate **B**, an acyl species stabilized by an agostic C–H bond. This transformation is slightly endergonic by 6.1 kcal/mol.

Upon coordination of PhSiH_3_, the first Si–H bond cleavage takes place with concomitant H-atom transfer from PhSiH_3_ to the C-atom of the acyl ligand to produce silyl complex **D** and acetaldehyde. This step has a rather high barrier of 23.0 kcal/mol and is slightly endergonic by 1.9 kcal/mol. In the following steps, from **E** to **G**, there will be H-transfer from the Si atom to the metal, forming a hydride and also O–H bond breaking in the incoming alcohol molecule, resulting in the formation of two new bonds: Si–O and a new Si–H bond. These two consecutive steps present small structural modifications and have a barrier of 10.2 kcal/mol and a free energy balance of −25.9 kcal/mol from **E** to **G**. Thus, in **E**, the presence of the neighbor alcohol molecule shifts the silyl ligand into an agostic Si–H--Fe interaction (d_Si–H_ = 1.62 Å and d_Fe–H_ = 1.70 Å). In **F**, the H-transfer from the Si atom to metal is almost accomplished (d_Si–H_ = 1.71 Å and d_Fe–H_ = 1.62 Å), while the new Si–O bond is almost formed (d_Si–O_ = 2.19 Å). This bond formation is completed in **G** (d_Si–O_ = 1.71 Å), while the H-transfer from the O- to the Si atom is halfway through, mediated by the metal.

It has to be mentioned that intermediate **B** alternatively may react first with *i*PrOH instead of PhSiH_3_ to form an alkoxide complex as a result of the cleavage of the O–H bond with concomitant release of acetaldehyde. This process, however, is very unfavorable by 51.3 kcal/mol and rather unlikely (see Figure S2 in the Supporting Information).

A slight rotation of the O*i*Pr moiety about the Si–O bond, from **G** to **G′**, allows the completion of the Si–H and Fe–H bond cleavage processes, yielding the hydride intermediate **H** in an accessible (Δ*G*‡ = 17.3 kcal/mol) and endergonic step (Δ*G* = 6.8 kcal/mol). Silane (H_2_SiPh(O*i*Pr)) loss in **H** affords **H’**, a 16e^–^ complex which adopts a square pyramidal structure and undergoes a facile isomerization moving the hydride ligand from the *cis* to the *trans* position of the pyrazole moiety to yield the active catalyst **I** (Δ*G*‡ = 6.0 kcal/mol, Δ*G* = −0.7 kcal/mol). This reaction completes the initiation process involving, overall, two Si–H bond activation steps and one O–H bond activation steps. The catalyst initiation process has a global barrier of 32.9 kcal/mol (measured from **A** to **TS**_**CD**_) and is favorable, from the thermodynamic point of view, with a free energy balance of Δ*G* = −9.8 kcal/mol. The value calculated for the overall barrier is high, taking into account the reaction conditions. However, it corresponds simply to the initiation process that only happens once. The barrier calculated for the catalytic cycle is lower (see below).

The catalytic cycle begins with the addition of an alkyne molecule to active species **I**. In the first step, from **J** to **K**, there is insertion of the alkyne molecule into the iron hydride bond, in a highly exergonic process (Δ*G* = −31.7 kcal/mol) with an activation barrier of 3.5 kcal/mol. In **J**, the alkyne is only weakly bound with an average Fe–C bond distance of 4.60 Å (Figure 3), while **K** is a vinyl complex, and the insertion process is finished. From **K**, addition of *i*PrOH leads to intermediate **L**, where *i*PrOH is only loosely bound and, then, to the formation of the isopropanol complex **M**. This is an endergonic process (Δ*G* = 14.0 kcal/mol) with a barrier of 14.8 kcal/mol (both measured from **K**). The reaction proceeds with H-atom transfer from the coordinated *i*PrOH ligand to the C-atom of the vinyl ligand, from **M** to **N**, releasing one molecule of styrene in a fairly easy and exergonic step (Δ*G* = −16.8 kcal/mol) with a barrier of 14.1 kcal/mol.

In the last steps, the addition of a new silane molecule to **N** yields **O**, where the silane molecule has only a weak interaction with the complex. In a concerted fashion, the silane forms one Si–O and one Fe–H bond via **TS**_**OP**_, leading to intermediate **P** (Figure 4). This process requires a free energy of activation of 14.0 kcal/mol and has free energy balance of 3.0 kcal/mol.

Subsequent Si–H bond cleavage and release of H_2_SiPh(O*i*Pr) afford complex **Q**. This is the last intermediate, actually being the active catalyst **I** containing the alkoxysilane weakly bound to the metal center with an Fe···O distance of 5.11 Å. From **Q**, exchange of the alkoxysilane SiH_2_Ph(O*i*Pr) with a new molecule of alkyne regenerates the initial species closing the cycle with a free energy balance of Δ*G* = 6.5 kcal/mol. The catalytic cycle presents an overall barrier of 28.1 kcal/mol, measured from **K** to **TS**_**MN**_, and a free energy balance of Δ*G* = −48.0 kcal/mol.

It is important to notice that the mechanism calculated and discussed above agrees reasonably well with the deuterium labeling studies described above (Scheme 4). On the one hand, the two entering H-atoms will both add *cis* to each other in the resulting olefin producing a *Z*-alkene and, on the other hand, of those two H-atoms, one comes from the alcohol, *i*PrO-H with the other coming from the silane, H-SiH_2_Ph (red and green H-atoms in the simplified cycle of Scheme 5).

**Scheme 6:**
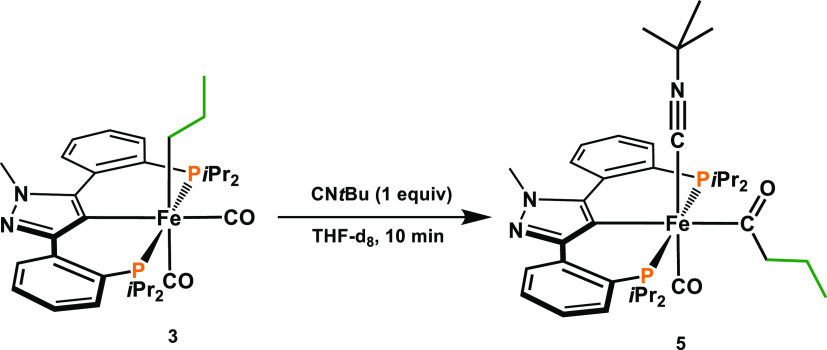
Trapping an Acyl Complex upon Treatment of **3** with CN*t*Bu

To prove whether the initiation via an acyl species is indeed taking place, complex **3** was treated with CN*t*Bu, which resulted in the formation of Fe(PCP-*i*Pr)(C(=O)CH_2_CH_2_CH_3_)(CO)(CN*t*Bu) (**5**) in 91% isolated yield (Scheme 6). Complex **5** was fully characterized by ^1^H, ^13^C{^1^H}, and ^31^P{^1^H} NMR and IR spectroscopy and high-resolution mass spectrometry. The IR spectrum contains strong stretching vibrations at 2113 (ν_CN*t*Bu_), 1897 (ν_CO_), and 1670 (ν_C=O_) which clearly indicates the coordination of one isocyanide, one CO, and an acyl ligand, respectively.

Complex **5** was tested as a catalyst for the semihydrogenation of phenylacetylene. However, under optimized conditions, styrene was obtained in only 23% yield. This may be due to the fact that in order to form the active species, *t*BuNC dissociation is required, which is apparently unfavorable.

## Conclusions

Carbonyl ligands are known to undergo migratory insertion into the metal carbon bond of an alkyl moiety, yielding coordinatively unsaturated acyl complexes, which are capable of activating weakly polar E–H bonds (E = –H, –C≡C-R, –BR_2_, –SiR_3_). The rate of alkyl migration typically follows the order *n*Pr > Et > Me. We successfully applied this concept to iron alkyl PCP pincer complexes. Two new bench-stable Fe(II) alkyl complexes [Fe(κ^3^PCP-PCP-*i*Pr)(CO)_2_(R)] (R = CH_2_CH_2_CH_3_, CH_3_) were obtained by treatment of [Fe(κ^3^PCP-PCP-*i*Pr)(CO)_2_(H)] with NaNH_2_ and subsequent addition of CH_3_CH_2_CH_2_Br and CH_3_I, respectively. The reaction proceeds via anionic Fe(0) intermediate Na[Fe(κ^3^PCP-PCP-*i*Pr)(CO)_2_]. The catalytic performance of both alkyl complexes was investigated for the transfer hydrogenation of terminal and internal alkynes utilizing PhSiH_3_ and *i*PrOH as a hydrogen source. Precatalyst activation is initiated by migration of the alkyl ligand to the carbonyl C atom of an adjacent CO ligand to form an acyl intermediate. In fact, the acyl complex Fe(PCP-*i*Pr)(C(=O)CH_2_CH_2_CH_3_)(CO)(CN*t*Bu) can be readily obtained by reacting CN*t*Bu with the propyl complex [Fe(κ^3^PCP-PCP-*i*Pr)(CO)_2_(CH_2_CH_2_CH_3_)].

In agreement with previous findings, the rate of alkyl migration follows the order *n*Pr > Me. Accordingly, [Fe(κ^3^PCP-PCP-*i*Pr)(CO)_2_(CH_2_CH_2_CH_3_)] is the more active catalyst. The reaction proceeds at room temperature with a catalyst loading as low as 0.5 mol %. There was no overhydrogenation observed, and in the case of internal alkynes, exclusively, *Z*-alkenes were formed. The implemented protocol tolerates a variety of electron-donating and electron-withdrawing functional groups including halides, nitriles, unprotected amines, and heterocycles. Mechanistic investigations including deuterium labeling studies and DFT calculations were undertaken to provide a reasonable reaction mechanism. After precatalyst activation, initiated by an incoming silane PhSiH_3_ and *i*PrOH addition, the active 16e hydride catalyst [Fe(κ^3^PCP-PCP-*i*Pr)(CO)(H)] is formed. This process involves the activation of two Si–H bonds and the O–H bond of the alcohol. The mechanism calculated for the catalytic cycle starts with alkyne insertion into the Fe–H bond of the hydride intermediate, forming a vinyl species that is further protonated by an incoming alcohol molecule. The cycle closes by means of product (olefin) liberation followed by addition of new silane molecule that will regenerate the hydride ligand and release the alkoxysilane byproduct. The calculated mechanism justifies the observed formation of *Z*-alkenes that both new H atoms are *cis* to each other, one coming from the alcohol O–H bond and the other coming from the silane Si–H bond, in reasonable agreement with the deuterium labeling studies presented.

## Supporting Information Available

Synthetic procedures, ^1^H, ^13^C{^1^H}, and ^31^P{H} NMR spectra of all compounds, crystallographic data, and complete computational details and corresponding references (PDF)
X-ray crystallographic data for **4**·**1.5****C_6_H_6_** (CCDC 2282394) (CIF)
Cartesian coordinates for DFT-optimized structures (XYZ) (XYZ)

## Supplementary Material

cs3c04156_si_001.pdf

cs3c04156_si_002.cif

cs3c04156_si_003.xyz
