# Supplementary material for: Selective Transfer Semihydrogenation of Alkynes Catalyzed by an Iron PCP Pincer Alkyl Complex
Source: ACS Catal. 2023 Oct 17;13(21):14012–22. doi: 10.1021/acscatal.3c04156 (PMC10629171; doi:10.1021/acscatal.3c04156)
Supplement: Supplementary file 1 — cs3c04156_si_001.pdf [file cs3c04156_si_001.pdf]

Selective Transfer-Semihydrogenation of Alkynes Catalyzed by an Iron PCP Pincer Alkyl Complex

Heiko Schratzberger,[†] Berthold Stöger,[‡] Luis F. Veiros,[$] Karl Kirchner*,[†]

[†] Institute of Applied Synthetic Chemistry, TU Wien, Getreidemarkt 9/163-AC, A-1060 Wien, Austria.
[‡] X-Ray Center, TU Wien, Getreidemarkt 9/163, A-1060 Wien, Austria.
[$] Centro de Química Estrutural, Institute of Molecular Sciences, Departamento de Engenharia Química, Instituto Superior Técnico, Universidade de Lisboa, Av. Rovisco Pais, 1049 001 Lisboa, Portugal

E-Mail Karl Kirchner: karl.kirchner@tuwien.ac.at

Supporting Information

TABLE OF CONTENTS

GENERAL INFORMATION	S2
SYNTHESES	S2-S5
X-RAY STRUCTURE DETERMINATION	S5
COMPUTATIONAL DETAILS	S5-S7
OPTIMIZATION REACTIONS FOR THE SEMIHYDROGENATION OF TERMINAL ALKYNES	S7
SYNTHETIC PROCEDURE FOR MERCURY DROP TEST	S7
SYNTHETIC PROCEDURE FOR POISONING WITH TRIMETHYLPHOSPHINE	S7
SYNTHETIC PROCEDURE FOR DETUTERATION STUDIES	S8
GENERAL PROCEDURE FOR SEMIHYDROGENATIONS	S8
SUBSTRATE SCOPE	S8-S12
NMR SPECTRA OF COMPLEXES	S13-S18
NMR SPECTRA OF SUBSTRATE SCOPE	S19-S29
^{2}H NMR spectra	S30-S32
REFERENCES	S33-S35

Experimental Section

General Information

All manipulations were performed under an inert atmosphere of argon by using Schlenk techniques or in an MBraun inert-gas glovebox. The solvents were purified according to standard procedures.[1] The deuterated solvents were purchased from Eurisotop SAS and dried over 4 Å molecular sieves. All starting materials are known compounds and were used as obtained from commercial resources. [Fe^{II}(κ^3*PCP*-PCP-*i*Pr)H($CO)_2$] (**1**) was synthesized according to literature.[2] ^{1}H, ^{13}C{^{1}H}, and ^{31}P{^{1}H} NMR spectra were recorded on Bruker AVANCE-250, AVANCE-400 and AVANCE-600 spectrometers. ^{1}H and ^{13}C{^{1}H} NMR spectra were referenced internally to residual protio-solvent and solvent resonances, respectively, and are reported relative to tetramethylsilane (δ = 0 ppm). ^{31}P{^{1}H} NMR spectra were referenced externally to H_3PO_4 (85 %) (δ = 0 ppm). Infrared spectra were recorded in attenuated total reflection (ATR) mode on a PerkinElmer Spectrum Two FT-IR spectrometer or on a Bruker Vertex 80 FT-IR spectrometer.

GC-MS analyses were conducted on a ISQ LT Single quadrupole MS (Thermo Fisher) directly interfaced to a TRACE 1300 Gas Chromatographic system (Thermo Fisher), using a Rxi-5Sil MS (30 m, 0.25 mm ID) cross-bonded dimethyl polysiloxane capillary column at a carrier flow of He 1.5 mL/min. The oven program was:

Method **A**: 40 °C (2.5 min) // 12 °C/min // 220 °C (2.5 min)

Method **B**: 100 °C (2 min) // 35 °C/min // 300 °C (4 min)

If not stated otherwise, Method **A** was used as default.

High resolution-accurate mass spectra were recorded on a hybrid Maxis Qq-aoTOF mass spectrometer (Bruker Daltonics, Bremen, Germany) fitted with an ESI source or an Agilent 6545 QTOF mass spectrometer equipped with an Agilent Dual AJS ESI ion source (Agilent Technologies, Santa Clara, CA, USA). Measured accurate mass data of the $[M]^+$ ions for confirming calculated elemental compositions were typically within ±5 ppm accuracy. The mass calibration was done with a commercial mixture of perfluorinated trialkyl-triazines (ES Tuning Mix, Agilent Technologies, Santa Clara, CA, USA).

Syntheses

Na[Fe(κ^3*PCP*-PCP-*i*Pr)($CO)_2$] (2)

1 (50 mg, 0.09 mmol) was put into a screw-cap vial and dissolved in THF (2 mL). Then 3 equiv. of $NaNH_2$ (10 mg, 0.26 mmol) was added. The suspension was stirred at room temperature for 16 h, whereupon the orange-brown solution turned dark violet. The mixture was filtered through a syringe filter and all volatiles were removed under reduced pressure. The residue was extracted with *n*-pentane (3 mL) and benzene (3 mL) to remove unreacted starting material and the dark violet solid was dried in *vacuo* affording 47 mg (91 %) of **2** as a violet solid. ^{1}H NMR (600 MHz, THF-d_8, 20 °C, ppm): δ = 8.06 (ddd, *J* = 7.8, 2.8, 1.4 Hz, 1H, C-H), 7.45 (ddd, *J* = 7.8, 4.7, 1.3 Hz, 1H, C-H), 7.41 (ddd, *J* = 7.9, 4.9, 1.3 Hz, 1H, C-H), 7.34 (dd, *J* = 7.7, 1.5 Hz, 1H, C-H), 7.18 (td, *J* = 7.5, 1.3 Hz, 1H, C-H), 7.11 (td, *J* = 7.6, 1.3 Hz, 1H, C-H), 7.08 (td, *J* = 7.4, 1.2 Hz, 1H, C-H), 7.02 (td, *J* = 7.4, 1.4 Hz, 1H, C-H), 3.92 (s, 3H, N-CH_3), 2.34 (ddt, *J* = 38.2, 14.8, 7.2 Hz, 4H, C*H*($CH_3)_2$), 1.13 (m, 12H, CH(C*H*$_3)_2$), 1.01 (dd, *J* = 11.5, 7.0 Hz, 6H, CH(C*H*$_3)_2$), 0.95 (td, *J* = 7.3, 3.3 Hz, 6H, CH(C*H*$_3)_2$). ^{13}C{^{1}H} NMR (151 MHz, THF-d_8, 20 °C, ppm): δ = 228.9 (t, *J* = 23.7 Hz, CO), 156.5 (d, *J* = 14.5 Hz, C_q), 147.9 (d, *J* = 16.6 Hz, C_q), 143.4 (d, *J* = 9.8 Hz, C_q), 139.2 (d, *J* = 10.1 Hz, C_q), 134.7 (dd, *J* = 42.3, 39.0 Hz, C_{ipso}), 133.4 (d, *J* = 24.4 Hz, C_q), 131.6 (dd, *J* = 24.2, 2.7 Hz, C_q), 128.7 (d, *J* = 24.2 Hz, C-H), 128.3 (d, *J* = 34.3 Hz, C-H), 127.6 (s, C-H), 127.0 (br, 2C, C-H), 126.6 (d, *J* = 5.6 Hz,

C-H), 124.0 (d, *J* = 4.1 Hz, C-H), 123.8 (d, *J* = 4.1 Hz, C-H), 39.8 (s, N-CH_3), 29.3 (d, *J* = 17.8 Hz, *C*H(CH_3)$_2$), 28.5 (d, *J* = 16.8 Hz, *C*H(CH_3)$_2$), 25.9 (d, *J* = 20.2 Hz, *C*H(CH_3)$_2$), 25.7 (br, *C*H(CH_3)$_2$), 20.7 (br, CH(*C*H$_3$)$_2$), 20.6 (d, *J* = 2.5 Hz, *C*H(CH_3)$_2$), 19.6 (br, *C*H(CH_3)$_2$), 19.2 (br, *C*H(CH_3)$_2$). ^{31}P{^{1}H} NMR (243 MHz, THF-d$_8$, 20 °C, ppm): δ = AB spin system, δ_A = 93.1 (1P), δ_B = 92.4 (1P), J_{PP} = 92.4 Hz. IR (ATR, cm^{-1}): 1774 (ν_{CO}), 1708 (ν_{CO})

[Fe(κ^3*PCP*-PCP-*i*Pr)($CH_2CH_2CH_3$)(CO)$_2$] (3)

1 (50 mg, 0.09 mmol) was put into a screw-cap vial and dissolved in THF (2 mL). Then 3 equiv. of $NaNH_2$ (10 mg, 0.26 mmol) was added. The suspension was stirred at room temperature for 16 h, whereupon the orange-brown solution turned dark violet. Then excess 1-bromopropane (500 µL, 5.4 mmol) was added and the suspension was stirred at RT for 16 h. All volatiles of the yellow suspension were removed in vacuo and the residue was extracted with benzene (5 mL) and the supernatant was filtered through a syringe filter. Again all volatiles were removed in vacuo and the residue was treated with *n*-pentane, whereupon a beige-brown precipitate formed. The supernatant was removed and the solid was dried in vacuo affording **3** as a beige solid with a yield of 34 mg (63 %). ^{1}H NMR (600 MHz, C_6D_6, 20 °C, ppm): δ = 8.77 (m, 1H, C-H), 7.28 (m, 2H, C-H), 7.06 (m, 5H), 3.71 (s, 3H, N-CH_3, isomer A), 3.70 (s, 3H, N-CH_3, isomer B), 2.60 (m, 1H, C*H*(CH_3)$_2$), 2.48 (dd, *J* = 15.1, 7.6 Hz, 1H, C*H*(CH_3)$_2$), 2.13 (m, 3H, C*H*(CH_3)$_2$ + Fe-CH_2-C*H*$_2$-CH_3 isomer B), 1.70 (d, *J* = 15.1 Hz, 1H, Fe-CH_2-C*H*$_2$-CH_3 isomer A), 1.56 (dd, *J* = 14.6, 6.8 Hz, 1H, CH(C*H*$_3$)$_2$), 1.50 (dd, *J* = 14.6, 6.8 Hz, 2H, CH(C*H*$_3$)$_2$), 1.40 (dd, *J* = 13.8, 7.0 Hz, 2H, CH(C*H*$_3$)$_2$), 1.29 (m, 6H, CH(C*H*$_3$)$_2$ + Fe-CH_2-CH_2-C*H*$_3$), 1.10 (m, 2H, Fe-C*H*$_2$-CH_2-CH_3), 1.00 (q, *J* = 6.8 Hz, 3H, CH(C*H*$_3$)$_2$), 0.84 (dd, *J* = 13.7, 7.0 Hz, 2H, CH(C*H*$_3$)$_2$), 0.74 (dd, *J* = 13.7, 7.1 Hz, 2H, CH(C*H*$_3$)$_2$), 0.62 (dd, *J* = 11.3, 6.8 Hz, 6H, CH(C*H*$_3$)$_2$), 0.50 (dd, *J* = 13.1, 7.0 Hz, 2H, CH(C*H*$_3$)$_2$), 0.46 (dd, *J* = 13.2, 6.4 Hz, 1H, CH(C*H*$_3$)$_2$). ^{13}C{^{1}H} NMR (151 MHz, C_6D_6, 20 °C, ppm): δ = 220.3 (t, *J* = 16.9 Hz, CO), 211.5 (t, *J* = 17.8 Hz, CO), 210.9 (t, *J* = 19.0 Hz, CO), 155.7 (d, *J* = 13.9 Hz, C_q, isomer B), 154.6 (d, *J* = 13.9 Hz, C_q, isomer A), 147.2 (d, *J* = 16.6 Hz, C_q, isomer A), 146.3 (d, *J* = 16.5 Hz, C_q, isomer B), 143.6 (d, *J* = 10.2 Hz, C_q, isomer B), 143.2 (d, *J* = 9.3 Hz, C_q, isomer A), 139.8 (d, *J* = 10.6 Hz, C_q, isomer A), 139.5 (d, *J* = 9.9 Hz, C_q, isomer B), 132.4 (s, C-H), 131.6 (s, C-H), 130.3 (m, C-H), 130.3 (d, *J* = 2.2 Hz, C-H), 130.2 (s, C-H), 129.6 (s, C-H), 129.5 (s, C-H), 128.6 (s, C-H), 128.5 (s, C-H), 127.7 (s, C-H), 127.6 (s, C_q, isomer A) 127.6 (s, C-H), 126.8 (d, *J* = 7.3 Hz, C-H), 125.6 (d, *J* = 5.8 Hz, C-H), 125.6 (d, *J* = 5.2 Hz, C-H), 125.5 (d, *J* = 5.2 Hz, C-H), 125.3 (d, *J* = 5.7 Hz, C-H), 124.6 (d, *J* = 36.3 Hz, C_q, isomer B), 123.9 (d, *J* = 35.1 Hz, C_q, isomer B) , 120.3 (d, *J* = 34.9 Hz, C_q, isomer A), 117.8 (dd, *J* = 39.6, 30.1 Hz, C_{ipso}, isomer A), 117.2 (t, *J* = 35.5 Hz, C_{ipso}, isomer B), 39.6 (s, N-CH_3, isomer B), 39.1 (s, N-CH_3, isomer A), 31.2 (br, Fe-CH_2-*C*H$_2$-CH_3, isomer A), 31.0 (br, Fe-CH_2-*C*H$_2$-CH_3, isomer B), 29.6 (d, *J* = 23.8 Hz, *C*H(CH_3)$_2$,isomer A), 28.5 (d, *J* = 23.6 Hz, *C*H(CH_3)$_2$, isomer B), 25.3 (dd, *J* = 12.3, 3.4 Hz, *C*H(CH_3)$_2$, isomer B), 24.8 (d, *J* = 19.5 Hz, *C*H(CH_3)$_2$, isomer A), 24.5 (d, *J* = 20.5 Hz, *C*H(CH_3)$_2$, isomer B), 24.2 (d, *J* = 9.1 Hz, *C*H(CH_3)$_2$, isomer B), 24.2 (d, *J* = 16.4 Hz, *C*H(CH_3)$_2$, isomer A), 23.8 (d, *J* = 19.1 Hz, *C*H(CH_3)$_2$, isomer A), 21.1 (br, CH(*C*H$_3$)$_2$), 21.1, (br, CH(*C*H$_3$)$_2$), 20.6 (br, CH(*C*H$_3$)$_2$), 19.7 (t, *J* = 17.6 Hz, Fe-*C*H$_2$- CH_2-CH_3, isomer A), 19.2 (br, CH(*C*H$_3$)$_2$), 18.9 (vt, *J* = 18.1 Hz, Fe-*C*H$_2$-CH_2-CH_3, isomer B), 18.8 (br, CH(*C*H$_3$)$_2$), 18.5 (d, *J* = 3.2 Hz, Fe-CH_2-CH_2-*C*H$_3$, isomer A), 18.5 (d, *J* = 3.2 Hz, Fe-CH_2-CH_2-*C*H$_3$, isomer B), 17.8 (d, *J* = 7.9 Hz, CH(*C*H$_3$)$_2$), 17.6 (d, *J* = 7.2 Hz, CH(*C*H$_3$)$_2$), 17.4 (d, *J* = 8.4 Hz, CH(*C*H$_3$)$_2$), 16.9 (d, *J* = 10.8 Hz, CH(*C*H$_3$)$_2$), 16.6 (t, *J* = 5.9 Hz, CH(*C*H$_3$)$_2$), 16.1 (m, CH(*C*H$_3$)$_2$). ^{31}P{^{1}H} NMR (162 MHz, C_6D_6, 20 °C, ppm): δ = AB spin system isomer A (main) , δ_A = 68.4 (1P), δ_B = 56.9 (1P), J_{PP} = 148.2 Hz, AB spin system isomer B (minor) , δ_A = 67.7 (1P), δ_B = 57.5 (1P), J_{PP} = 148.3 Hz. IR (ATR, cm^{-1}): 1969 (ν_{CO}), 1907 (ν_{CO}). HRMS (ESI$^+$-MS/MS, MeOH): m/z calc for $C_{33}H_{47}FeN_2NaO_2P_2$ [M+H]$^+$ 621.2457, found 621.2461.

[Fe(κ^3*PCP*-PCP-*i*Pr)(CH$_3$)(CO)$_2$] (4)

1 (50 mg, 0.09 mmol) was put into a screw-cap vial and dissolved in THF (2 mL). Then 3 equiv. of $NaNH_2$ (10 mg, 0.26 mmol) was added. The suspension was stirred at room temperature for 16 h, whereupon the orange-brown solution turned dark violet. Then excess methyl iodide (84 µL, 1.3 mmol) was added and the suspension was stirred at room temperature for 16 h. All volatiles of the suspension were removed in vacuo and the residue was extracted with benzene (5 mL) and the supernatant was filtered through a syringe filter. Again all volatiles were removed in vacuo and the residue was treated with *n*-pentane, whereupon a yellow precipitate formed. The supernatant was removed and the solid was dried in vacuo affording **4** as a beige-yellow solid with a yield of 36 mg (71 %). ^{1}H NMR (600 MHz, C_6D_6, 20 °C, ppm): δ = 8.79 (ddd, *J* = 7.9, 3.5, 1.4 Hz, 1H, isomer A, C-H), 8.72 (ddd, *J* = 7.9, 3.5, 1.4 Hz, 1H, isomer B, C-H), 7.30 (m, 1H), 7.24 (ddd, *J* = 7.7, 3.4, 1.2 Hz, 1H, C-H), 7.15-6.97 (m, 5H, C-H), 3.71 (s, 3H, isomer B, N-CH$_3$), 3.70 (s, 3H, isomer A, N-CH$_3$), 2.59 (m, 1H, C*H*(CH$_3$)$_2$), 2.47 (m, 1H, C*H*(CH$_3$)$_2$), 2.22 (m, 1H, C*H*(CH$_3$)$_2$), 2.04 (m, 1H, C*H*(CH$_3$)$_2$), 1.47 (m, 6H, CH(C*H$_3$*)$_2$), 1.23 (m, 6H, CH(C*H$_3$*)$_2$), 0.78 (dd, *J* = 13.6, 7.1 Hz, 2H, CH(C*H$_3$*)$_2$), 0.72 (dd, *J* = 13.6, 7.0 Hz, 1H, CH(C*H$_3$*)$_2$), 0.61 (m, 6H, CH(C*H$_3$*)$_2$), 0.50 (dd, *J* = 13.4, 7.1 Hz, 1H, CH(C*H$_3$*)$_2$), 0.38 (dd, *J* = 13.2, 7.0 Hz, 2H, CH(C*H$_3$*)$_2$), 0.18 (t, *J* = 8.4 Hz, 3H, Fe-CH$_3$), 0.10 (t, *J* = 8.1 Hz, 2H, Fe-CH$_3$). ^{13}C{^{1}H} NMR (151 MHz, C_6D_6, 20 °C, ppm): δ = 221.1 (q, *J* = 15.8 Hz, CO), 212.1 (t, *J* = 17.7 Hz, CO), 211.5 (t, *J* = 18.6 Hz, CO), 155.9 (d, *J* = 13.4 Hz, C$_q$, isomer B), 154.7 (d, *J* = 14.0 Hz, C$_q$, isomer A), 147.0 (d, *J* = 16.4 Hz, C$_q$, isomer A), 146.4 (d, *J* = 16.7 Hz, C$_q$, isomer B), 143.4 (d, *J* = 10.2 Hz, C$_q$, isomer B), 143.1 (d, *J* = 10.0 Hz, C$_q$, isomer A), 139.7 (d, *J* = 10.8 Hz, C$_q$, isomer A), 139.4 (d, *J* = 10.2 Hz, C$_q$, isomer B), 132.2 (s, C-H), 131.5 (s, C-H), 130.4 (s, C-H), 130.3 (d, *J* = 1.6 Hz, C-H), 130.2 (s, C-H), 129.7 (s, C-H), 129.5 (d, *J* = 1.6 Hz, C-H), 129.4 (s, C-H), 128.5 (s, C-H), 128.4 (s, 2C, C-H), 127.7 (d, *J* = 6.1 Hz, C$_q$, isomer A), 127.6 (d, *J* = 6.7 Hz, C-H), 126.8 (d, *J* = 7.4 Hz, C-H), 125.6 (vt, *J* = 5.8 Hz, 2C, C-H), 125.4 (d, *J* = 5.5 Hz, C-H), 124.9 (d, *J* = 35.9 Hz, C$_q$, isomer B), 124.0 (d, *J* = 34.4 Hz, C$_q$ isomer B), 120.7 (d, *J* = 34.5 Hz, C$_q$, isomer A), 119.3 (dd, *J* = 33.6, 3.2 Hz, C$_{ipso}$, isomer A), 119.2 (dd, *J* = 33.6, 3.8 Hz, C$_{ipso}$, isomer B), 39.5 (s, N-CH$_3$, isomer B), 39.1 (s, N-CH$_3$, isomer A), 29.9 (d, *J* = 22.8 Hz, *C*H(CH$_3$)$_2$, isomer A), 28.7 (d, *J* = 22.8 Hz, *C*H(CH$_3$)$_2$, isomer B), 24.4 (d, *J* = 18.4 Hz, *C*H(CH$_3$)$_2$, isomer B), 24.2 (d, *J* = 19.0 H, *C*H(CH$_3$)$_2$, isomer A z), 24.0 (d, *J* = 19.7 Hz, *C*H(CH$_3$)$_2$, isomer A), 23.9 (d, *J* = 19.9 Hz, *C*H(CH$_3$)$_2$, isomer B), 23.6 (d, *J* = 16.6 Hz, *C*H(CH$_3$)$_2$, isomer B), 22.4 (d, *J* = 16.9 Hz, *C*H(CH$_3$)$_2$, isomer A), 19.6 (br, CH(*C*H$_3$)$_2$), 19.6 (br, CH(*C*H$_3$)$_2$), 19.4 (br, CH(*C*H$_3$)$_2$), 19.1 (br, CH(*C*H$_3$)$_2$), 18.4 (d, *J* = 3.7 Hz, CH(*C*H$_3$)$_2$), 18.3 (d, *J* = 3.4 Hz, CH(*C*H$_3$)$_2$), 18.1 (br, CH(*C*H$_3$)$_2$), 18.0 (br, CH(*C*H$_3$)$_2$), 17.6 (d, *J* = 7.1 Hz, br, CH(*C*H$_3$)$_2$), 17.5 (br, CH(*C*H$_3$)$_2$), 17.4 (br, CH(*C*H$_3$)$_2$), 17.2 (d, *J* = 8.1 Hz, CH(*C*H$_3$)$_2$), 16.3 (d, *J* = 5.2 Hz, CH(*C*H$_3$)$_2$), 15.4 (d, *J* = 5.0 Hz, CH(*C*H$_3$)$_2$), 14.3 (br, CH(*C*H$_3$)$_2$), -3.9 (vt, *J* = 19.9 Hz, Fe-CH$_3$, isomer A), -5.1 (vt, *J* = 19.4 Hz, Fe-CH$_3$, isomer B). ^{31}P{^{1}H} NMR (162 MHz, C_6D_6, 20 °C, ppm): δ = AB spin system isomer A (main) , δ_A = 66.1 (1P), δ_B = 60.3 (1P), J_{PP} = 149.1 Hz, AB spin system isomer B (minor) , δ_A = 65.4 (1P), δ_B = 60.3 (1P), J_{PP} = 163.8 Hz. IR (ATR, cm^{-1}): 1970 (ν_{CO}), 1908 (ν_{CO}). HRMS (ESI$^+$-MS/MS, MeOH): m/z calc for $C_{31}H_{43}FeN_2NaO_2P_2$ [M+H]$^+$ 593.2144, found 593.2144.

[Fe(κ^3*PCP*-PCP-*i*Pr)(C(=O)CH$_2$CH$_2$CH$_3$)(CO)(CN*t*Bu) (5)

To **3** (15 mg, 0.024 mmol) dissolved in THF-d$_8$ was added *t*BuNC (1 M in THF-d$_8$, 0.024 mmol, 24 µL). After 10 minutes the orange solution turned yellow-orange. All volatiles were removed affording 16 mg (94 %) of **5** as an orange solid. ^{1}H NMR (600 MHz, THF-d$_8$, 20 °C, ppm): δ = 8.29 (m, 1H, C-H), 7.66(m, 1H, C-H), 7.52 (m, 1H, C-H), 7.44 (m, 2H, C-H), 7.31 (m, 1H, C-H), 7.23 (m, 1H, C-H), 7.15 (m, 1H, C-H), 3.99 (s, 3H, N-CH$_3$, isomer A), 3.90 (s, 3H, N-CH$_3$, isomer B), 3.08 (m, 1H, C*H*(CH$_3$)$_2$), 2.96 (m, 2H, C*H*(CH$_3$)$_2$ + Fe-(CO)C*H$_2$*-CH$_2$-CH$_3$, isomer A), 2.48 (m, 0.5H, Fe-(CO)C*H$_2$*-CH$_2$-CH$_3$, isomer B), 2.20 (m, 0.5H, Fe-(CO)C*H$_2$*-CH$_2$-CH$_3$, isomer B), 1.98 (m, 1H, C*H*(CH$_3$)$_2$), 1.79 (m, 1H, C*H*(CH$_3$)$_2$), 1.56 (m, 6H, CH(C*H$_3$*)$_2$), 1.38 (m, 3H, CH(C*H$_3$*)$_2$), 1.28 (m, 5H, CH(C*H$_3$*)$_2$), 0.92 (s, 3H, CN-C(C*H$_3$*)$_3$, isomer B), 0.89 (s,

6H, CN-C(CH_3)$_3$, isomer A), 0.87 (m, 2H, Fe-(CO)CH_2-CH_2-CH_3), 0.81 (m, 4H, CH(CH_3)$_2$), 0.44 (m, 4H, CH(CH_3)$_2$ + Fe-(CO)CH_2-CH_2-CH_3), 0.37 (m, 2H, CH(CH_3)$_2$), 0.25 (m, 3H, CH(CH_3)$_2$). $^{13}C\{^1H\}$ NMR (151 MHz, THF-d_8, 20 °C, ppm): δ = 269.3 (t, *J* = 23.2 Hz, Fe-C(=O)Pr, isomer A), 268.1 (t, *J* = 24.7 Hz, Fe-C(=O)Pr, isomer B), 220.7 (t, *J* = 16.4 Hz, CO, isomer B), 220.5 (t, *J* = 16.8 Hz, CO, isomer A), 164.6 (bt, *J* = 8.4 Hz, Fe-*C*N*t*Bu), 157.5 (d, *J* = 14.9 Hz, C_q, isomer B), 155.3 (d, *J* = 12.5 Hz, C_q, isomer A), 149.3 (d, *J* = 18.0 Hz, C_q, isomer A), 147.5 (d, *J* = 16.0 Hz, C_q, isomer B), 146.7 (d, *J* = 9.7 Hz, C_q, isomer B), 145.3 (d, *J* = 10.2 Hz, C_q, isomer A), 143.0 (d, *J* = 10.3 Hz, C_q isomer A), 141.8 (d, *J* = 10.7 Hz, C_q, isomer A), 131.8 (s, C-H, isomer A), 130.9 (d, *J* = 6.8 Hz, C-H, isomer A), 130.1 (s, C-H, isomer B), 129.9 (d, *J* = 2.0 Hz, C-H, isomer A), 129.9 (d, *J* = 1.9 Hz, C-H, isomer A), 129.7 (s, C-H, isomer B), 129.7 (d, *J* = 2.0 Hz, C-H, isomer B), 128.8 (d, *J* = 6.1 Hz, C-H, isomer B), 128.6 (d, *J* = 6.3 Hz, C-H, isomer B), 128.4 (d, *J* = 32.5 Hz, C_q, isomer B), 127.6 (d, *J* = 7.2 Hz, C-H, isomer A), 126.7 (d, *J* = 7.3 Hz, C-H, isomer B), 126.1 (d, *J* = 5.4 Hz, C-H, isomer A), 125.5 (d, *J* = 5.7 Hz, C-H, isomer B), 125.4 (d, *J* = 4.9 Hz, C-H, isomer B), 125.1 (d, *J* = 31.5 Hz, C_q, isomer A), 123.4 (d, *J* = 32.6 Hz, C-H, isomer A), 122.2 (dd, *J* = 37.1, 32.2 Hz, Fe-C_{ipso}, isomer B), 122.1 (dd, *J* = 36.1, 32.1 Hz, Fe-C_{ipso}, isomer A), 120.2 (d, *J* = 32.6 Hz, C_q, isomer B), 64.7 (s, Fe-C(=O)*C*$H_2CH_2CH_3$, isomer B), 63.8 (s, Fe-C(=O)*C*$H_2CH_2CH_3$, isomer A), 56.5 (s, CN*C*(CH_3)$_3$, isomer A), 56.4 (s, CN*C*(CH_3)$_3$, isomer B), 40.2 (s, N-CH_3, isomer A), 39.8 (s, N-CH_3, isomer B), 31.6 (s, Fe-C(=O)CH_2*C*H_2CH_3, isomer B), 31.1 (s, Fe-C(=O)CH_2*C*H_2CH_3, isomer A), 30.5 (s, CNC(CH_3)$_3$, isomer B), 30.3 (s, CNC(*C*H_3)$_3$, isomer A), 29.6 (d, *J* = 22.0 Hz, *C*H(CH_3)$_2$, isomer B), 29.1 (dd, *J* = 17.6, 2.0 Hz, *C*H(CH_3)$_2$, isomer A), 28.7 (d, *J* = 21.9 Hz, *C*H(CH_3)$_2$, isomer A), 28.2 (d, *J* = 17.0 Hz, , *C*H(CH_3)$_2$, isomer B), 26.2 (d, *J* = 18.8 Hz, *C*H(CH_3)$_2$, isomer B), 25.9 (d, *J* = 20.2 Hz, *C*H(CH_3)$_2$, isomer A), 24.9 (d, *J* = 19.8 Hz, *C*H(CH_3)$_2$, isomer A), 24.4 (d, *J* = 20.5 Hz, *C*H(CH_3)$_2$, isomer B), 20.5 (d, *J* = 3.5 Hz, CH(*C*H_3)$_2$), 20.2 (br, CH(*C*H_3)$_2$), 19.4 (s, Fe-C(=O)CH_2*C*H_2CH_3, isomer B), 19.4 (s, Fe-C(=O)CH_2*C*H_2CH_3, isomer A), 19.1 (d, *J* = 3.0 Hz, CH(*C*H_3)$_2$), 19.0 (m, CH(*C*H_3)$_2$), 18.3 (m, CH(*C*H_3)$_2$), 18.0 (m, CH(*C*H_3)$_2$), 17.6 (d, *J* = 5.1 Hz, CH(*C*H_3)$_2$), 17.4 (d, *J* = 5.0 Hz, CH(*C*H_3)$_2$), 16.5 (m, CH(*C*H_3)$_2$), 14.6 (s, Fe-C(=O)CH_2CH_2*C*H_3, isomer A), 14.6 (s, Fe-C(=O)CH_2CH_2*C*H_3, isomer B). $^{31}P\{^1H\}$ NMR (243 MHz, THF-d_8, 20 °C, ppm): δ = isomer A: 63.6 (d, *J* = 104.1 Hz), 57.7(d, *J* = 104.1 Hz); isomer B: 63.9 (d, *J* = 104.1 Hz), 56.6 (d, *J* = 104.1 Hz), IR (ATR, cm^{-1}): 2113 (ν_{CNtBu}), 1897 (ν_{CO}), 1670 ($\nu_{C=O}$). HRMS (ESI$^+$-MS/MS, THF): m/z calc for $C_{38}H_{56}FeN_3O_2P_2$ $[M+H]^+$ 704.3191, found 704.3195.

X-ray Structure Determination

X-ray diffraction data of **3**·1.5C_6H_6 (CCDC 2282394) were collected at *T* = 100 K in a dry stream of nitrogen on a Bruker Kappa APEX II diffractometer system using graphite-monochromatized Mo-*K*α radiation (λ = 0.71073 Å) and fine sliced *φ*- and *ω*-scans. Data were reduced to intensity values with SAINT and an absorption correction was applied with the multi-scan approach implemented in SADABS.[3] The structures were solved by the dual-space approach implemented in SHELXT[4] and refined against F^2 with SHELXL.[5] Non-hydrogen atoms were refined with anisotropic displacement parameters. H atoms were placed in calculated positions and thereafter refined as riding on the parent atoms. A methyl and a CO ligand were refined as occupationally disordered, with the overall occupancy fixed to 1. The C—O distance of the minor CO position was restrained to 1.16 Å. Molecular graphics were generated with the program MERCURY.[6]

Computational Details

Calculations were performed using the Gaussian 09 software package[7] and the PBE0 functional, without symmetry constraints. That functional uses a hybrid generalized gradient approximation

(GGA), including 25 % mixture of Hartree-Fock[8] exchange with DFT[9] exchange-correlation, given by Perdew, Burke and Ernzerhof functional (PBE).[10] The optimized geometries were obtained with the Stuttgart Effective Core Potentials and associated basis set (SDD)[11] for Fe, and a standard 6-31G(d,p)[12] for the remaining elements (basis b1). Transition state optimizations were performed with the Synchronous Transit-Guided Quasi-Newton Method (STQN) developed by Schlegel *et al*,[13] following extensive searches of the Potential Energy Surface. Frequency calculations were performed to confirm the nature of the stationary points, yielding one imaginary frequency for the transition states and none for the minima. Each transition state was further confirmed by following its vibrational mode downhill on both sides and obtaining the minima presented on the energy profile. The electronic energies (E_{b1}) obtained at the PBE0/b1 level of theory were converted to free energy at 298.15 K and 1 atm (G_{b1}) by using zero point energy and thermal energy corrections based on structural and vibration frequency data calculated at the same level.

Single point energy calculations were performed on the geometries obtained at the PBE0/b1 level using the same functional and a 6-311++G(d,p) basis set.[14] Solvent effects (THF) were accounted for in all calculations (including geometry optimizations) by means of the Polarisable Continuum Model (PCM) initially devised by Tomasi and coworkers[15] with radii and non-electrostatic terms of the SMD solvation model, developed by Truhler *et al.*[16] The free energy values presented (G_{b2}-D3) were corrected for dispersion by means of Grimme DFT-D3 method[17] with Becke and Johnson short distance damping,[18] being derived from the electronic energy values obtained at the PBE0-D3/6-311++G(d,p)//PBE0/b1 level (E_{b2}-D3) according to the following expression:

$$(G_{b2}\text{-D3}) = (E_{b2}\text{-D3}) + G_{b1} - E_{b1}$$

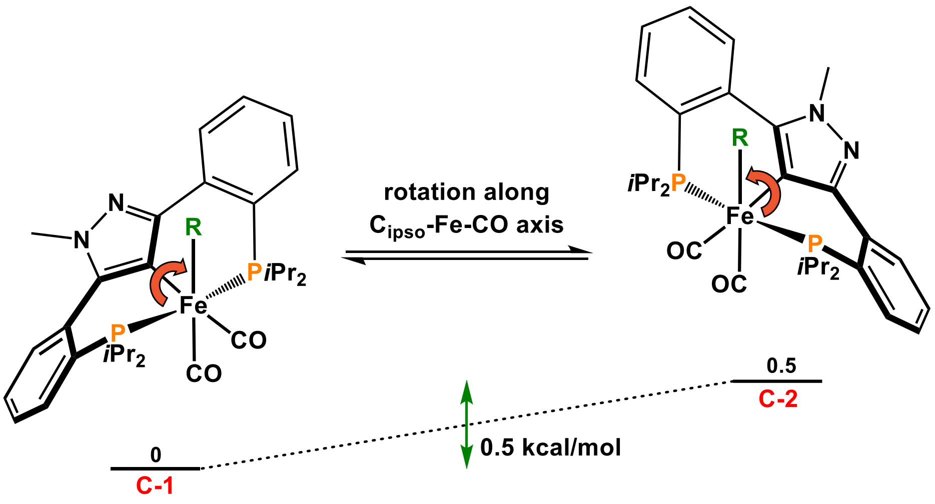

Figure S1. Conformational isomers (C-1 and C-2) of complexes **3** and **4** through rotation along the axis formed by C_{ipso}-Fe-CO. Rotation of the six-membered rings is slow on NMR timescale resulting in two observable conformers in NMR spectra.

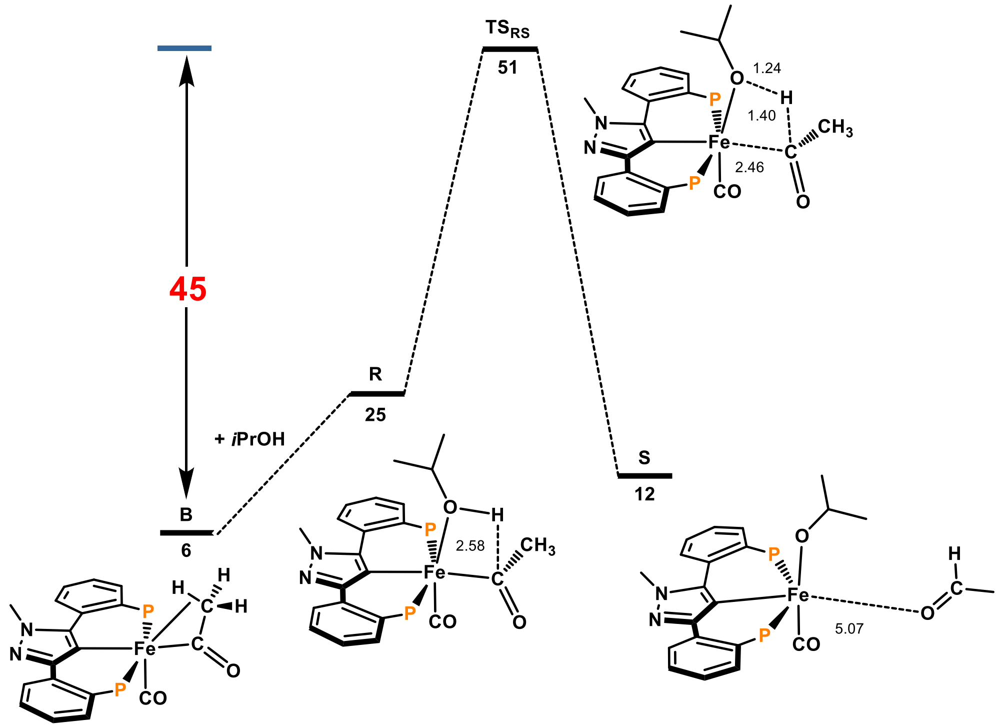

Figure S2. Free energy profile for the pre-catalyst activation with *i*PrOH. Free energies (kcal/mol) are referred to [Fe(κ^3*PCP*-PCP-*i*Pr)(CO)$_2$(CH$_3$)] (**A**).

Optimization reactions for the semihydrogenation of terminal alkynes

Inside an argon flushed glovebox, a screw-cap vial (8 mL) was charged with phenylacetylene (18-35 µL, 0.16-0.32 mmol, 1 equiv.), phenylsilane (96 %, 21-41 µL, 0.16-0.32 mmol) and 2-propanol (12-25 µL, 0.16-0.32 mmol), **3** or **4** (0.25-1 mol%) and solvent (0.6 mL) were added. The sealed vial was transferred out of the glovebox and stirred at room temperature for 24 h. The samples were exposed to air and analyzed *via* GC-MS. Conversions were determined by the ratios of the respective peak-areas of alkene to alkyne without addition of an internal standard (in no case could fully hydrogenated product or hydrosilylation products be detected).

Synthetic procedure for mercury drop test

Inside an argon flushed glovebox, a screw-cap vial (8 mL) was charged with phenylacetylene (18 µL, 0.161 mmol, 1 equiv.), phenylsilane (96 %, 21 µL, 0.161 mmol), 2-propanol (15 µL, 0.201 mmol), **3** (0.5 mol%), THF (0.6 mL) and 1 drop of mercury. The sealed vial was transferred out of the glovebox and stirred at room temperature for 24 h. The samples were exposed to air and analyzed *via* GC-MS. No significant decrease in reactivity could be observed.

Synthetic procedure for poisoning with trimethylphosphine

Inside an argon flushed glovebox, a screw-cap vial (8 mL) was charged with phenylacetylene (18 µL, 0.161 mmol, 1 equiv.), phenylsilane (96 %, 21 µL, 0.161 mmol), 2-propanol (15 µL, 0.201 mmol), **3** (0.5 mol%), THF (0.6 mL) and PMe$_3$ (1 M in THF, 8 µL, 10 equiv). The sealed vial was transferred out of

the glovebox and stirred at room temperature for 24 h. The samples were exposed to air and analyzed *via* GC-MS.
A drop of conversion to 2 % was detected.

Synthetic procedure for deuteration studies

Inside an argon flushed glovebox, a screw-cap vial (8 mL) was charged with phenylacetylene or phenylacetylene-d$_1$, respectively (0.161 mmol, 1 equiv.), phenylsilane (96 %, 21 µL, 0.161 mmol), 2-propanol/2-proanol-d$_1$/2-propanol-d$_8$ (0.201 mmol), **3** (0.5 mol%) and THF (0.6 mL). Mesitylene (11.1 µL, 0.081 mmol) was added as an internal standard. The sealed vial was transferred out of the glovebox and stirred at room temperature for 24 h.
The degree of deuteration was determined *via* ^{2}H NMR measurements.

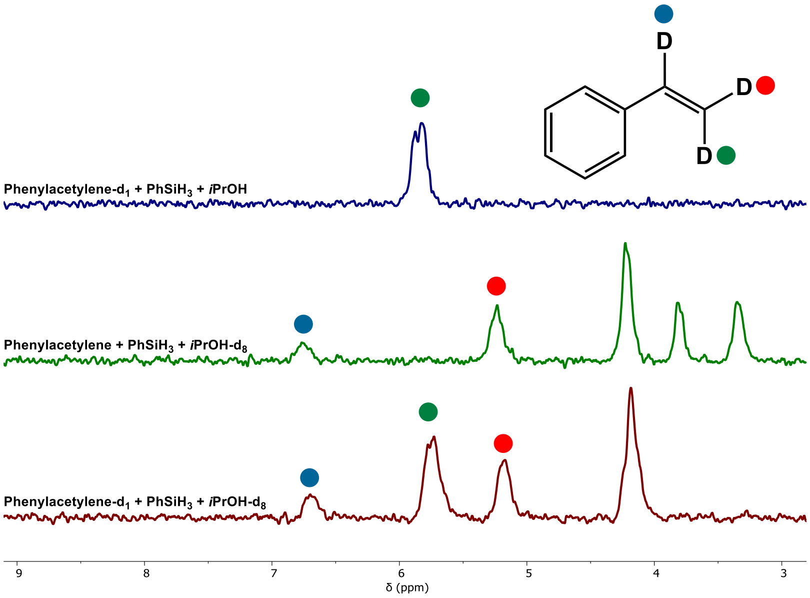

Figure S3. Deuterium distribution in styrene determined *via* ^{2}H NMR (38 MHz, THF, 20 °C)

General Procedure for Semihydrogenations

Inside an argon flushed glovebox, a screw-cap vial (8 mL) was charged with alkyne (0.161 mmol, 1 equiv.), phenylsilane (96 %, 21 µL, 0.161 mmol), 2-propanol (15 µL, 0.201 mmol) for terminal alkynes (19 µL, 0.242 mmol) for internal alkynes, **3** (0.5 mol%) and THF (0.6 mL). Mesitylene (11.1 µL, 0.081 mmol) was added as an internal standard. The sealed vial was transferred out of the glovebox and stirred at room temperature for 24 h. The samples were exposed to air and analyzed *via* GC-MS and NMR.
Conversions and yields were determined *via* NMR measurements of the reaction mixture.

Substrate Scope

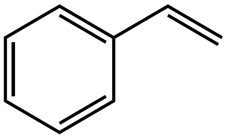

Styrene (A1):
Phenylacetylene (18 µL, 0.161 mmol), yield: 99 % (according to ^{1}H-NMR)
RT (GC): 5.817 min, MS: 104.151 m/z [M]$^+$

^{1}H-NMR (400 MHz, THF-d$_8$, 20 °C, ppm): δ = 6.72 (dd, *J* = 17.7, 10.9 Hz, 1H, C*H*=CH$_2$), 5.76 (dd, *J* = 17.7, 1.1 Hz, 1H, CH=C*H*$_2$), 5.20 (dd, *J* = 10.9, 1.1 Hz, 1H, CH=C*H*$_2$).

These spectroscopic data correspond to reported data.[19]

4-Fluorostyrene (A2):

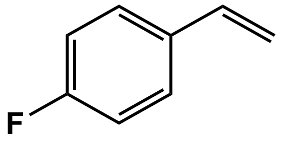

4-Fluorophenylacetylene (20 mg, 0.161 mmol), yield: 97 % (according to ^{1}H-NMR)

RT (GC): 5.936 min, MS: 122.135 m/z [M]$^+$

^{1}H-NMR (600 MHz, THF-d$_8$, 20 °C, ppm): δ = 6.70 (dd, *J* = 17.6, 10.9 Hz, 1H, C*H*=CH$_2$), 5.70 (d, *J* = 17.6 Hz, 1H, CH=C*H*$_2$), 5.18 (d, *J* = 10.9 Hz, 1H, CH=C*H*$_2$).

These spectroscopic data correspond to reported data.[19]

4-Chlorostyrene (A3):

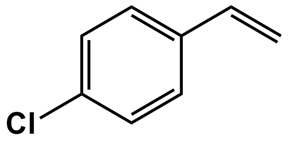

4-Chlorophenylacetylene (22 mg, 0.161 mmol), yield: 98 % (according to ^{1}H-NMR)

RT (GC): 8.667 min, MS: 138.127 m/z [M]$^+$

^{1}H-NMR (400 MHz, THF-d8, 20 °C, ppm): δ = 6.69 (dd, *J* = 17.6, 10.9 Hz, 1H, C*H*=CH$_2$), 5.77 (dd, *J* = 17.6, 0.9 Hz, 1H, CH=C*H*$_2$), 5.24 (dd, *J* = 10.9, 0.9 Hz, 1H, CH=C*H*$_2$).

These spectroscopic data correspond to reported data.[19]

4-Methylstyrene (A4)

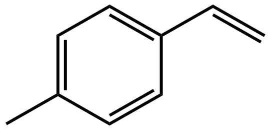

4-Methylphenylacetylene (19 mg, 0.161 mmol), yield: 72 % (according to ^{1}H-NMR)

RT (GC): 7.558 min, MS: 118.136 m/z [M]$^+$

^{1}H-NMR (600 MHz, THF-d$_8$, 20 °C, ppm): δ = 6.67 (dd, *J* = 17.6, 10.9 Hz, 1H, C*H*=CH$_2$), 5.70 (dd, *J* = 17.6, 1.1 Hz, 1H, CH=C*H*$_2$), 5.13 (dd, *J* = 10.9, 1.1 Hz, 1H, CH=C*H*$_2$), 2.30 (s, 3H, -CH$_3$)

These spectroscopic data correspond to reported data.[19]

4-(*tert*-Butyl)styrene (A5):

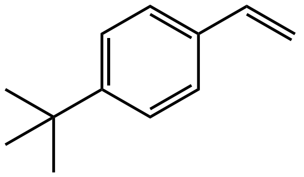

4-(*tert*-Butyl)phenylacetylene (29 µL, 0.161 mmol), yield: 95 % (according to ^{1}H-NMR)

RT (GC): 10.616 min, MS: 160.211 m/z [M]$^+$

^{1}H-NMR (600 MHz, THF-d$_8$, 20 °C, ppm): δ = 6.69 (dd, *J* = 17.6, 10.9 Hz, 1H, C*H*=CH$_2$), 5.71 (dd, *J* = 17.6, 1.3 Hz, 1H, CH=C*H*$_2$), 5.14 (dd, *J* = 10.9, 1.2 Hz, 1H, CH=C*H*$_2$), 1.32 (s, 9H, -C(CH$_3$)$_3$).

These spectroscopic data correspond to reported data.[20]

4-Vinylanisole (A6):

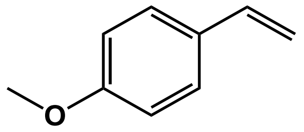

4-Ethynylanisole (21 µL, 0.161 mmol), yield: 99 % (according to ^{1}H-NMR)

RT (GC): 9.725 min, 134.161 m/z [M]$^+$

^{1}H-NMR (600 MHz, THF-d$_8$, 20 °C, ppm): δ = 6.65 (dd, *J* = 17.6, 10.9 Hz, 1H, C*H*=CH$_2$), 5.60 (dd, *J* = 17.6, 1.1 Hz, 1H, CH=C*H*$_2$), 5.06 (dd, *J* = 10.9, 1.1 Hz, 1H, CH=C*H*$_2$), 3.75 (s, 3H, OCH$_3$).

These spectroscopic data correspond to reported data.[19]

4-Vinylbenzonitrile (A7):

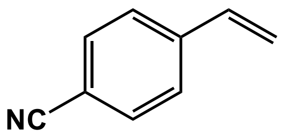

4-Ethynylbenzonitrile (20 mg, 0.161 mmol), yield: 44 % (according to ^{1}H-NMR)

RT (GC): 10.534 min, MS: 129.154 m/z $[M]^+$

^{1}H-NMR (600 MHz, THF-d_8, 20 °C, ppm): δ = 6.77 (dd, *J* = 17.6, 10.9 Hz, 1H, C*H*=CH_2), 5.95 (d, *J* = 17.6 Hz, 1H, CH=CH_2), 5.41 (d, *J* = 10.9 Hz, 1H, CH=CH_2).

These spectroscopic data correspond to reported data.[21]

4-Nitrostyrene (A8):

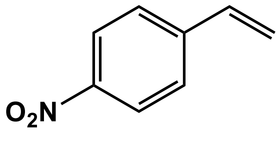

4-Nitrophenylacetylene (24 mg, 0.161 mmol), yield: 64 % (according to ^{1}H-NMR)

RT (GC): 11.922 min, MS: 149.148 m/z $[M]^+$

^{1}H-NMR (600 MHz, THF-d_8, 20 °C, ppm): δ = 6.84 (dd, *J* = 17.6, 10.9 Hz, 1H, C*H*=CH_2), 6.01 (d, *J* = 17.6 Hz, 1H, CH=CH_2), 5.47 (d, *J* = 11.0 Hz, 1H, CH=CH_2).

These spectroscopic data correspond to reported data.[21]

4-Vinylacetophenone (A9):

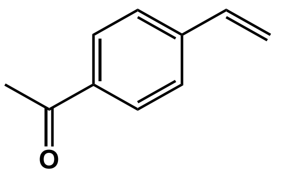

4-Ethynylacetophenone (23 mg, 0.161 mmol), yield: 57 % (according to ^{1}H-NMR)

RT (GC): 11.624 min, MS: 146.172 m/z $[M]^+$

^{1}H-NMR (600 MHz, THF-d_8, 20 °C, ppm): δ = 6.79 (dd, *J* = 17.6, 11.0 Hz, 1H, C*H*=CH_2), 5.92 (d, *J* = 17.6 Hz, 1H, CH=CH_2), 5.35 (d, *J* = 11.0 Hz, 1H, CH=CH_2), 2.52 (s, 3H, C(=O)CH_3).

These spectroscopic data correspond to reported data.[22]

3-Methylstyrene (A10):

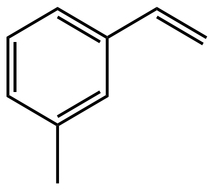

3-Methylphenylacetylene (20 µL, 0.161 mmol), yield: 95 % (according to ^{1}H-NMR)

RT (GC) 7.483 min, MS: 118.148 m/z $[M]^+$

^{1}H NMR (600 MHz, THF-d_8, 20 °C, ppm): δ = 6.68 (dd, *J* = 17.6, 10.9 Hz, 1H, C*H*=CH_2), 5.74 (dd, *J* = 17.6, 1.1 Hz, 1H, CH=CH_2), 5.17 (dd, *J* = 10.9, 1.2 Hz, 1H, CH=CH_2), 2.32 (s, 3H, -CH_3).

These spectroscopic data correspond to reported data.[19]

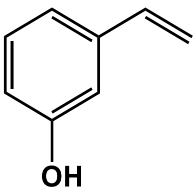

3-Vinylphenol (A11):

3-Ethynylphenol (19 mg, 0.161 mmol), yield: n.d .

no product formation observed

3-Vinylaniline (A12):

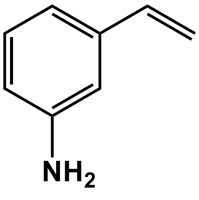

3-Ethynylaniline (17 µL, 0.161 mmol), yield: 98 % (according to ^{1}H-NMR)

RT (GC): 10.531 min, MS: 119.149 m/z $[M]^+$

^{1}H-NMR (400 MHz, THF-d_8, 20 °C, ppm): δ = 6.97 (t, *J* = 7.7 Hz, 1H, C-H), 6.66 (m, 1H, C-H), 6.64 (d, *J* = 7.5 Hz, 1H, C-H), 6.59 (dd, *J* = 17.6, 10.9 Hz, 1H, C*H*=CH_2), 6.48 (ddd, *J* = 7.9, 2.3, 1.0 Hz, 1H, C-H), 5.64 (dd, *J* = 17.6, 1.3 Hz, 1H, CH=CH_2), 5.09 (dd, *J* = 10.9, 1.2 Hz, 1H, CH=CH_2), 4.41 (br, 2H, -NH_2)

These spectroscopic data correspond to reported data.[23]

2-Vinylpyridine (A13):

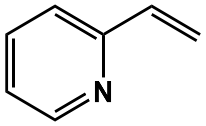

2-Ethynylpyridine (16 µL, 0.161 mmol), yield: 69 % (according to ^{1}H-NMR)

RT (GC): 6.490 min, MS: 105.138 m/z $[M]^+$

^{1}H-NMR (600 MHz, THF-d_8, 20 °C, ppm): δ = 6.79 (dd, *J* = 17.4, 10.7 Hz, 1H, C*H*=CH_2), 6.27 (dd, *J* = 17.4, 1.8 Hz, 1H, CH=CH_2), 5.38 (dd, *J* = 10.7, 1.8 Hz, 1H, CH=CH_2).

These spectroscopic data correspond to reported data.[24]

3-Vinylthiophen (A14):

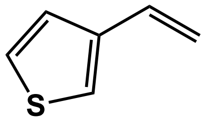

3-Ethynylthiophen (16 µL, 0.161 mmol), yield: 82 % (according to ^{1}H-NMR)

RT (GC): 6.011 min, MS: 110.108 m/z $[M]^+$

^{1}H-NMR (600 MHz, THF-d_8, 20 °C, ppm): δ = 6.72 (dd, *J* = 17.6, 10.9 Hz, 1H, C*H*=CH_2), 5.58 (dd, *J* = 17.5, 1.3 Hz, 1H, CH=CH_2), 5.13 (dd, *J* = 10.9, 1.4 Hz, 1H, CH=CH_2).

These spectroscopic data correspond to reported data.[22]

3-Phenyl-1-propene (A15):

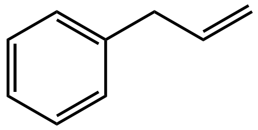

3-Phenyl-1-propyne (20 µL, 0.161 mmol),yield: 14 % (according to ^{1}H-NMR)

RT (GC): 6.715 min, MS: 118.178 m/z $[M]^+$

^{1}H-NMR (600 MHz, THF-d_8, 20 °C, ppm): δ = 5.96 (ddt, *J* = 16.8, 10.0, 6.7 Hz, 1H, C*H*=CH_2), 5.06 (dq, *J* = 17.0, 1.7 Hz, 1H, CH=CH_2), 5.03 (ddt, *J* = 10.1, 2.3, 1.3 Hz, 1H, CH=CH_2).

These spectroscopic data correspond to reported data.[25]

1-Octene (A16)

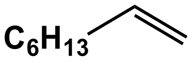

1-Octyne (24 µL, 0.161 mmol), yield: 86 % (according to ^{1}H-NMR)

RT (GC): 4.103, MS: 112.215 m/z $[M]^+$

^{1}H-NMR (400 MHz, THF) δ 5.80 (ddt, *J* = 16.9, 10.2, 6.7 Hz, 1H, C*H*=CH_2), 5.01 – 4.96 (m, 1H, CH=CH_2), 4.91 (ddt, *J* = 10.2, 2.3, 1.2 Hz, 1H, CH=CH_2).

These spectroscopic data correspond to reported data.[26]

6-Chloro-1-hexene (A17):

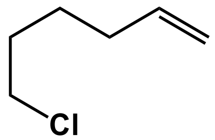

6-Chloro-1-hexyne (20 µL, 0.161 mmol), yield: 90 % (according to ^{1}H-NMR)

RT (GC): 5.079 min, MS: 118.174 m/z $[M]^+$

^{1}H NMR (600 MHz, THF-d_8, 20 °C, ppm): δ = 5.79 (ddt, *J* = 17.0, 10.2, 6.7 Hz, 1H, C*H*=CH_2), 5.00 (dq, *J* = 17.1, 1.7 Hz, 1H, CH=CH_2), 4.93 (m, 1H, CH=CH_2).

These spectroscopic data correspond to reported data.[27]

1-Vinyl-1-cyclohexene (A18):

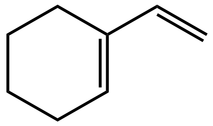

1-Ethynyl-1-cyclohexene (19 µL, 0.161 mmol), yield: 99 % (according to ^{1}H-NMR)

RT (GC): 6.099 min, MS: 108.172 m/z $[M]^+$

^{1}H-NMR (600 MHz, THF-d_8, 20 °C, ppm): δ = 6.31 (dd, *J* = 17.5, 10.7 Hz, 1H, C*H*=CH_2), 5.72 (m, 1H, -CH_2-C*H*=CR_2), 5.03 (ddq, *J* = 17.5, 1.6, 0.8 Hz, 1H, CH=C*H*$_2$), 4.83 (m, 1H, CH=C*H*$_2$).

These spectroscopic data correspond to reported data.[22]

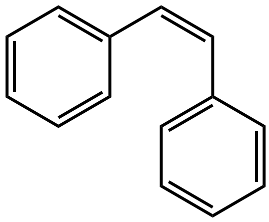

(*Z*)-Stilbene (A19)

Diphenylacetylene (29 mg, 0.161 mmol), yield: 99 % (according to ^{1}H-NMR)

RT (GC): 14.048 min, MS: 180.232 m/z $[M]^+$

^{1}H-NMR (600 MHz, THF-d_8, 20 °C, ppm): δ = 6.60 (s, 2H, -HC=CH-).

These spectroscopic data correspond to reported data.[28]

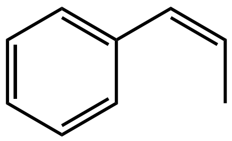

(*Z*)-1-Methylstyrene (A20):

1-Phenyl-1-propyne (20 µL, 0.161 mmol), yield: 47 % (according to ^{1}H-NMR)

RT(GC): 7.443 min, MS: 118.172 m/z $[M]^+$

^{1}H-NMR (600 MHz, THF-d_8, 20 °C, ppm): δ = 6.46 (dd, *J* = 11.6, 1.9 Hz, 1H, C*H*=CH(CH_3)), 5.79 (dq, *J* = 11.8, 7.2 Hz, 1H, CH=C*H*(CH_3)), 1.91 (dd, *J* = 7.2, 1.9 Hz, 3H, CH=CH(C*H*$_3$)).

These spectroscopic data correspond to reported data.[28]

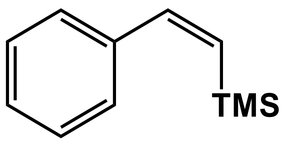

(*Z*)-β-(Trimethylsilyl)styrene (A21):

1-(Trimethylsilyl)phenylacetylene (31 µL, 0.161 mmol), yield: 90 % (according to ^{1}H-NMR)

RT(GC): 10.075 min, MS: 176.160 m/z $[M]^+$

^{1}H-NMR (600 MHz, THF-d_8, 20 °C, ppm): δ = 7.38 (d, *J* = 15.3 Hz, 1H, Ph*H*C=CH-TMS), 5.83 (d, *J* = 15.1 Hz, 1H, PhHC=C*H*-TMS), 0.07 (s, 9H, TMS).

These spectroscopic data correspond to reported data.[28]

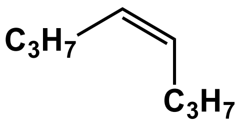

(*Z*)-4-Octene (A22):

4-Octyne (24 µL, 0.161 mmol), yield: 75 % (according to ^{1}H-NMR)

RT (GC): 4.256 min, MS: 112.216 m/z $[M]^+$

^{1}H-NMR (600 MHz, THF-d_8, 20 °C, ppm): δ = 5.36 (ddd, *J* = 5.7, 4.4, 1.2 Hz, 2H, -HC=CH-).

These spectroscopic data correspond to reported data.[28]

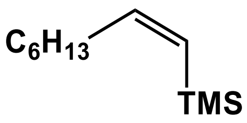

(*Z*)-1-(Trimethylsilyl)-1-octene (A23):

1-(Trimethylsilyl)-1-octyne (29 mg, 0.161 mmol), yield: 73 % (according to ^{1}H-NMR)

RT (GC): 9.419 min, MS: 184.259 m/z $[M]^+$

^{1}H-NMR (600 MHz, THF-d_8, 20 °C, ppm): δ = 6.30 (dt, *J* = 14.0, 7.4 Hz, 1H), 5.47 (d, *J* = 14.0 Hz, 1H), 0.12 (s, 9H).

These spectroscopic data correspond to reported data.[28]

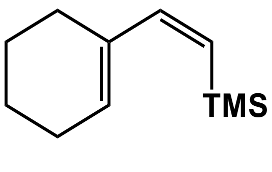

(*Z*)-1-[2-(Trimethylsilyl)ethenyl]-1-cyclohexene (A24):

1-(Trimethylsilylethynyl)-1-cyclohexene (29 mg, 0.161 mmol), yield: n.d.

no product formation observed

NMR spectra of complexes

^{1}H NMR (600 MHz, THF-d_8, 20 °C) of Na[Fe(κ^3*PCP*-PCP-*i*Pr)(CO)$_2$] (**2**)

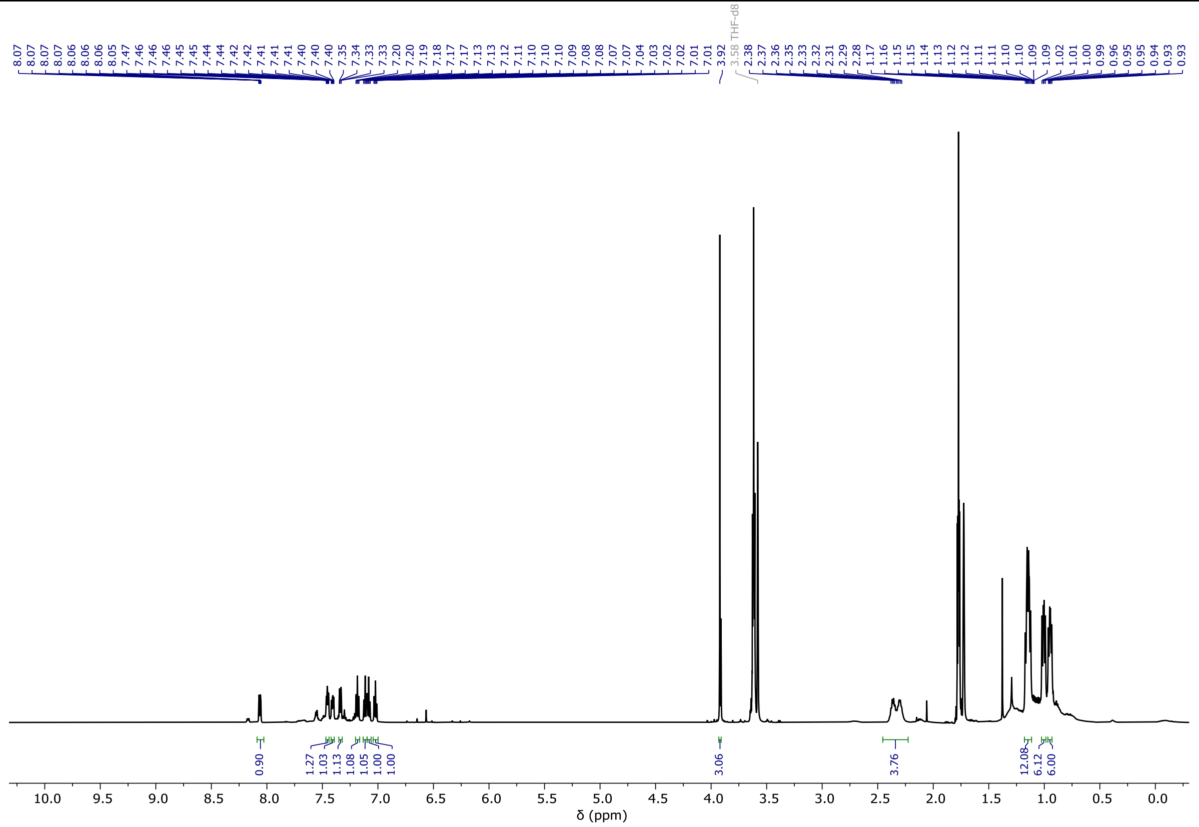

^{13}C{^{1}H} NMR (151 MHz, THF-d_8, 20 °C) of Na[Fe(κ^3*PCP*-PCP-*i*Pr)(CO)$_2$] (**2**)

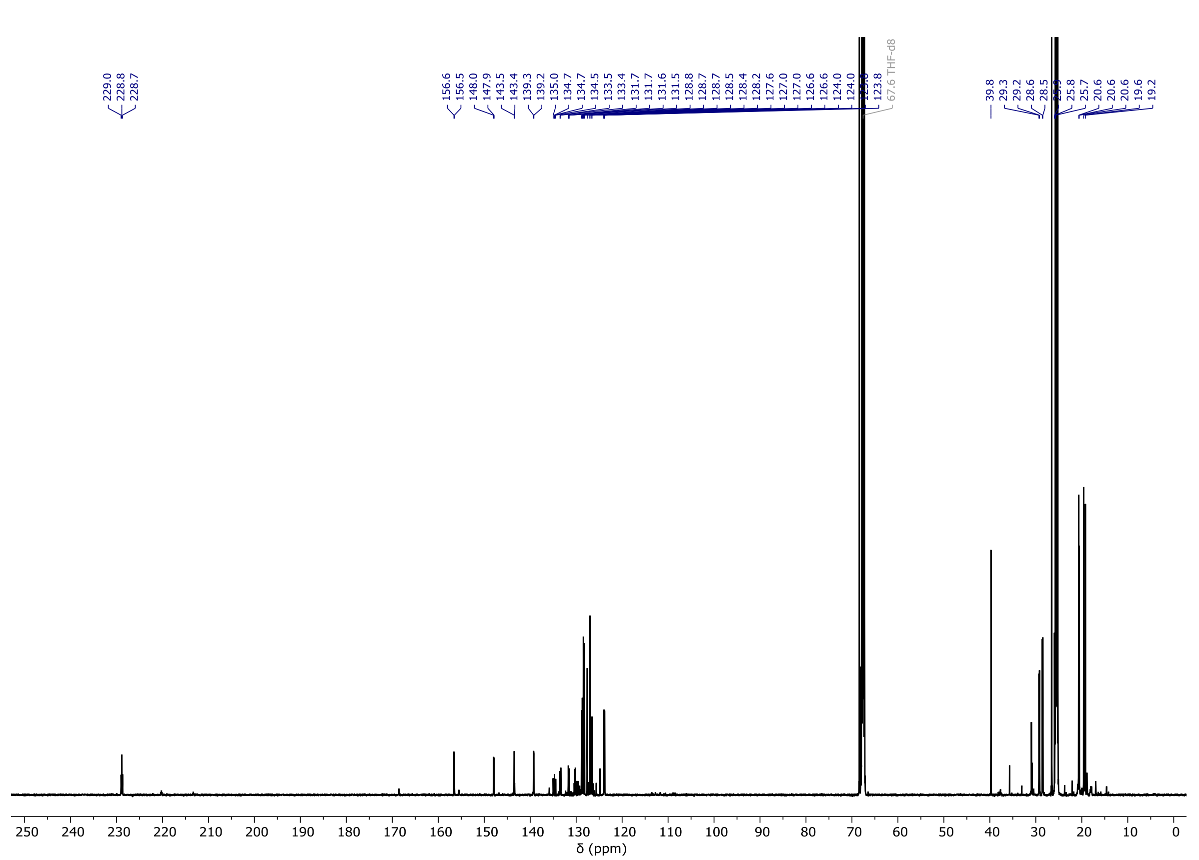

$^{31}P\{^1H\}$ NMR (243 MHz, THF-d_8, 20 °C) of Na[Fe(κ^3*PCP*-PCP-*i*Pr)$(CO)_2$] (**2**)

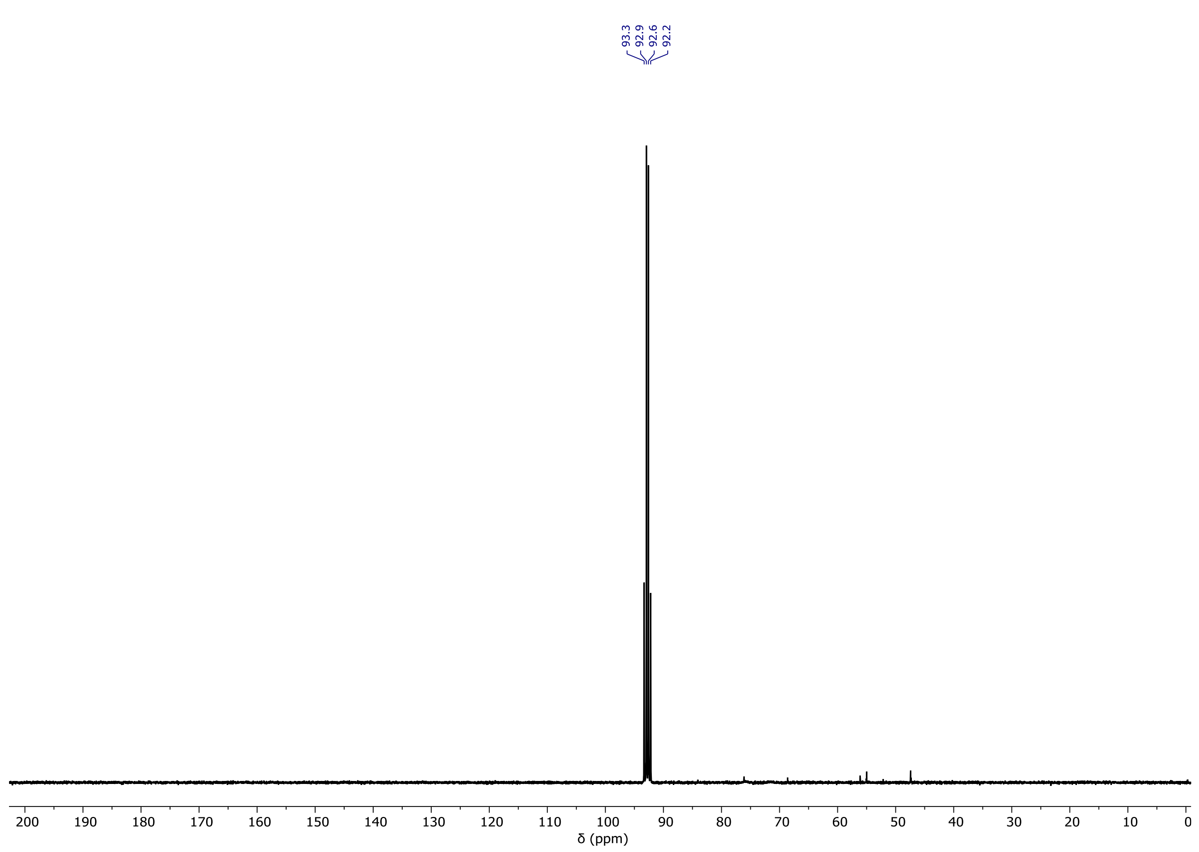

^1H NMR (600 MHz, C_6D_6, 20 °C) of Fe(κ^3*PCP*-PCP-*i*Pr)$(CH_2CH_2CH_3)(CO)_2$ (**3**)

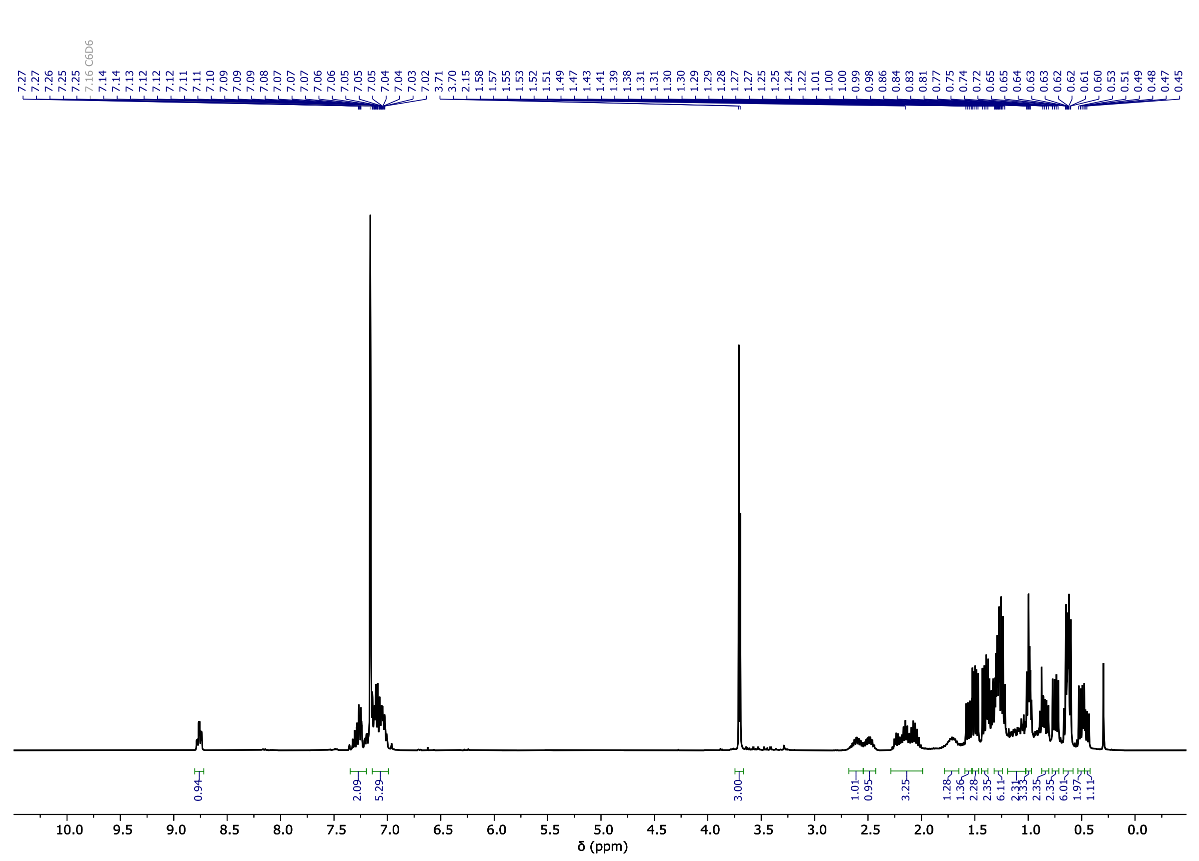

$^{13}C\{^1H\}$-APT NMR (151 MHz, C_6D_6, 20 °C) of Fe(κ^3*PCP*-PCP-*i*Pr)($CH_2CH_2CH_3$)$(CO)_2$ (**3**)

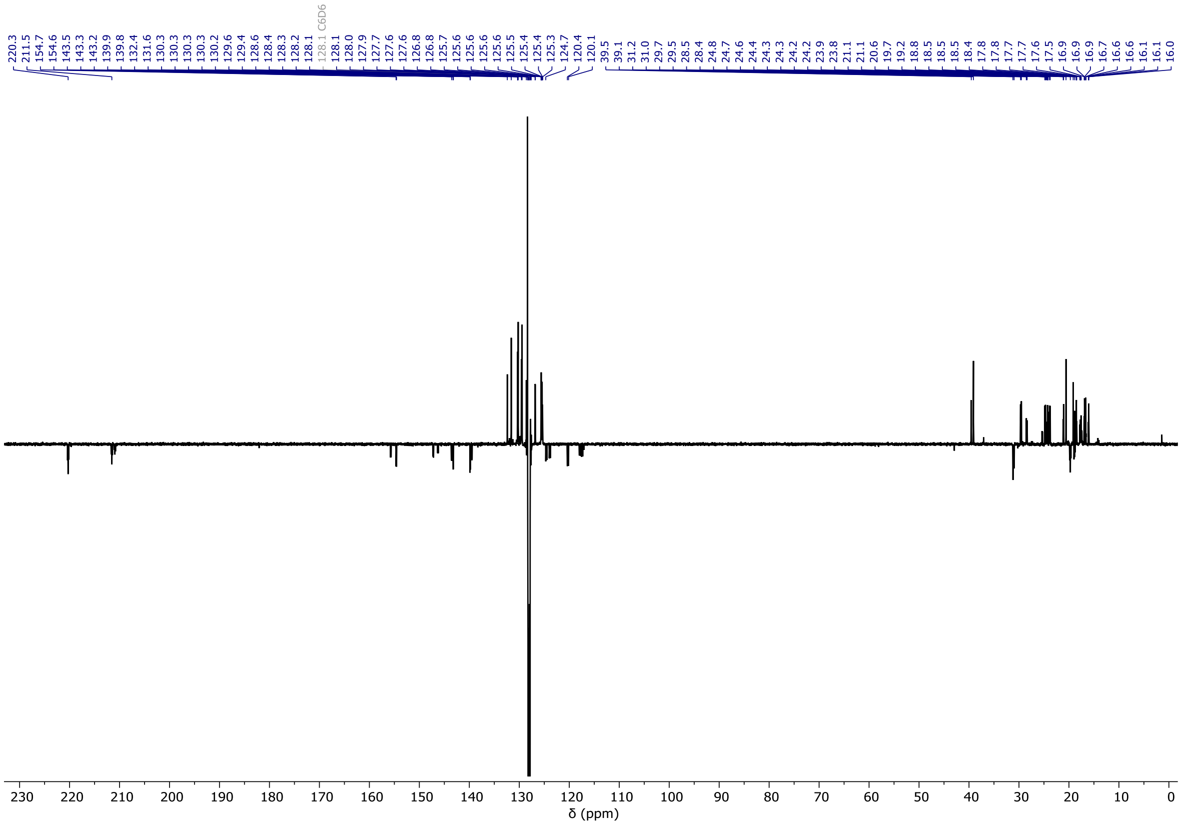

$^{31}P\{^1H\}$ NMR (162 MHz, C_6D_6, 20 °C) of Fe(κ^3*PCP*-PCP-*i*Pr)($CH_2CH_2CH_3$)$(CO)_2$ (**3**)

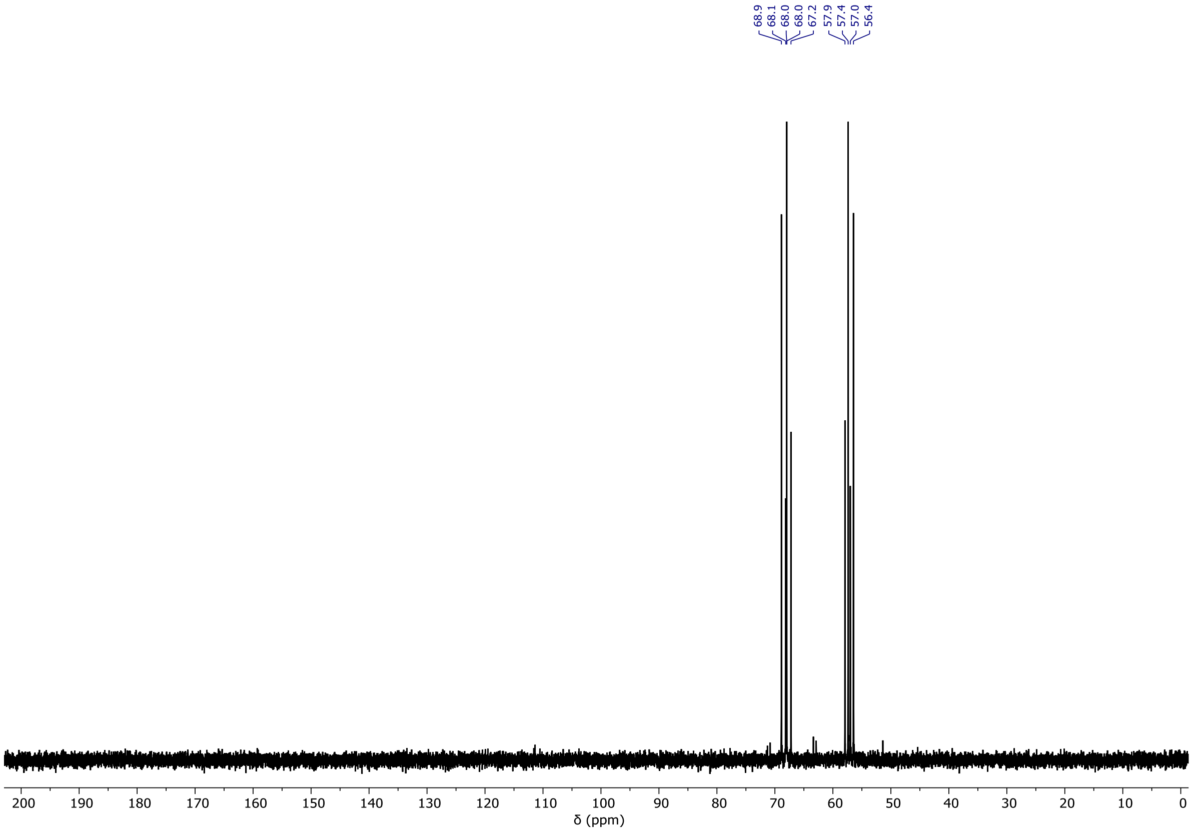

^{1}H NMR (600 MHz, C_6D_6, 20 °C) of Fe(κ^3*PCP*-PCP-*i*Pr)(CH_3)$(CO)_2$ (**4**)

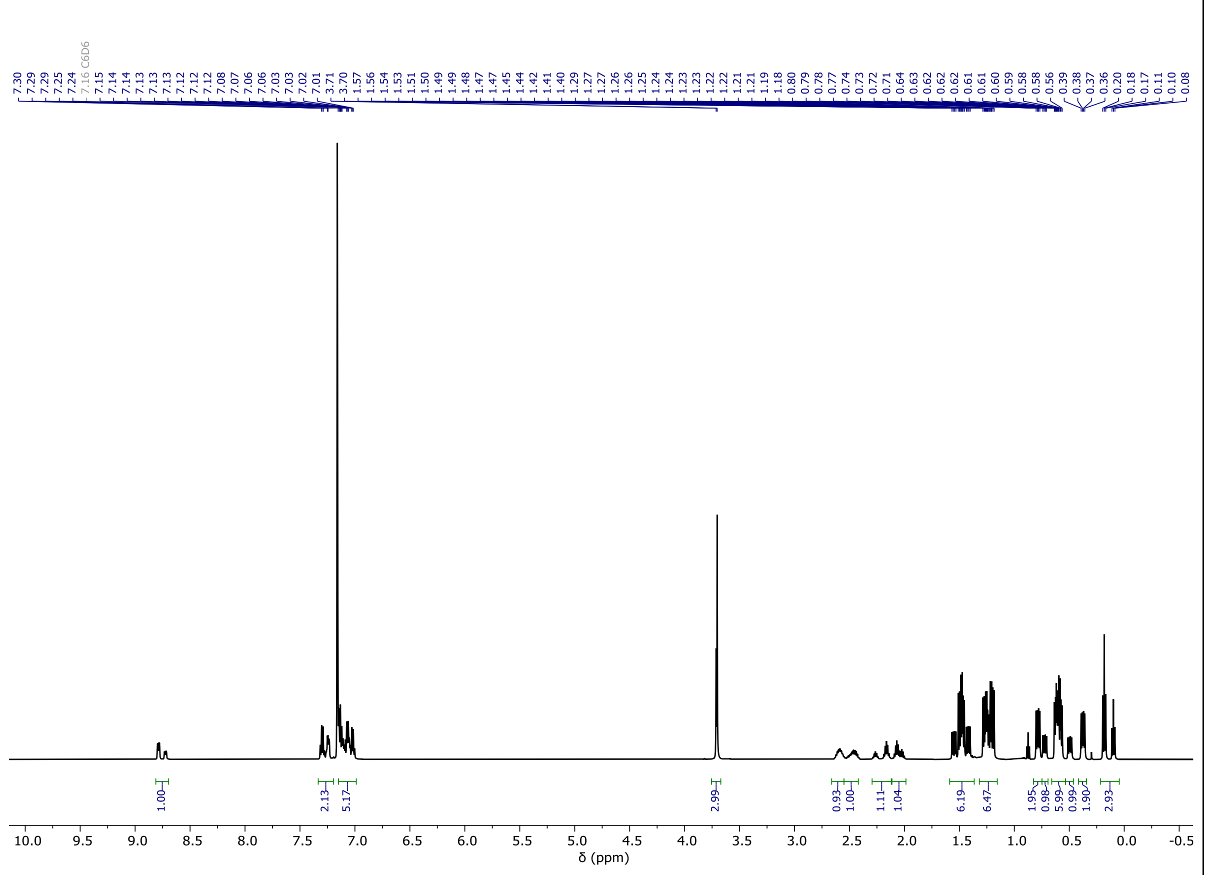

^{13}C{^{1}H} NMR (151 MHz, C_6D_6, 20 °C) of Fe(κ^3*PCP*-PCP-*i*Pr)(CH_3)$(CO)_2$ (**4**)

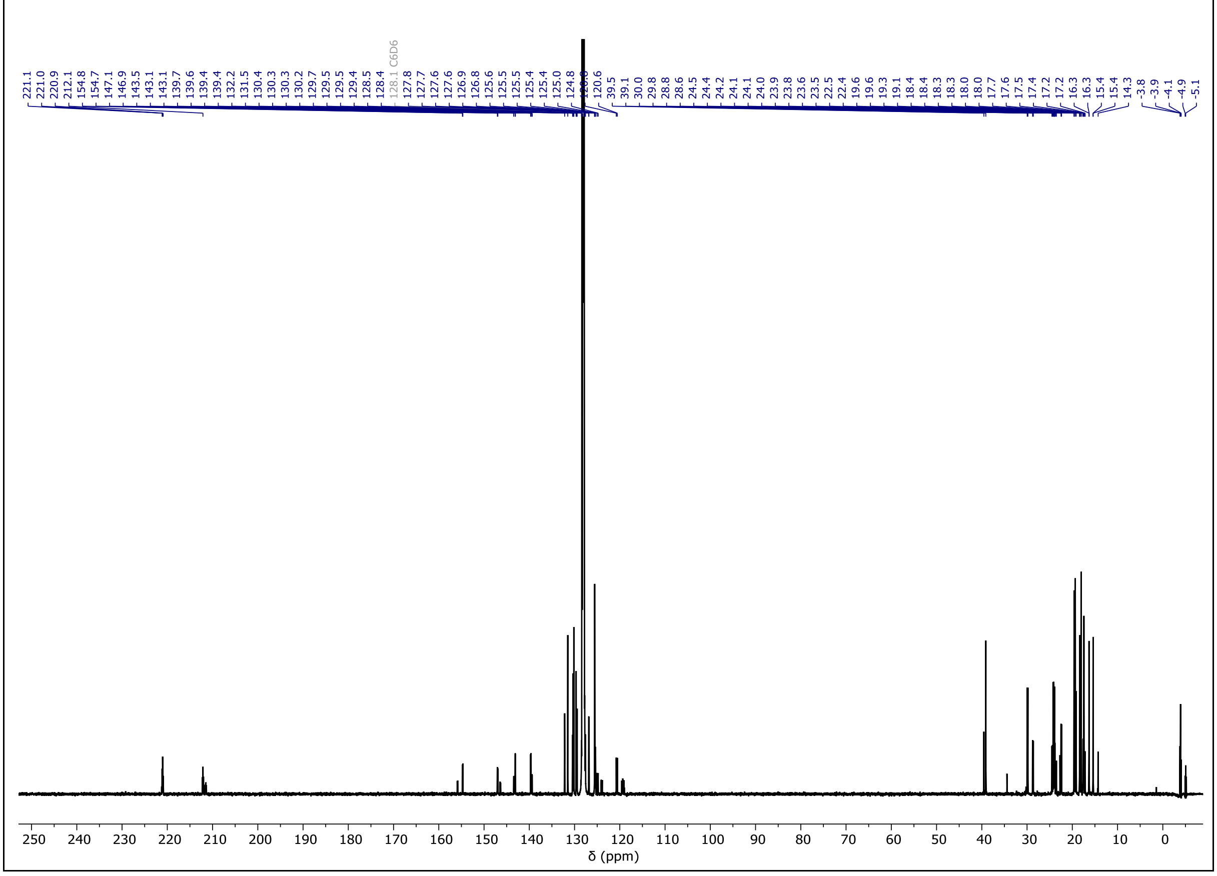

$^{31}P\{^{1}H\}$ NMR (162 MHz, C_6D_6, 20 °C) of Fe(κ[3]*PCP*-PCP-*i*Pr)(CH_3)$(CO)_2$ (**4**)

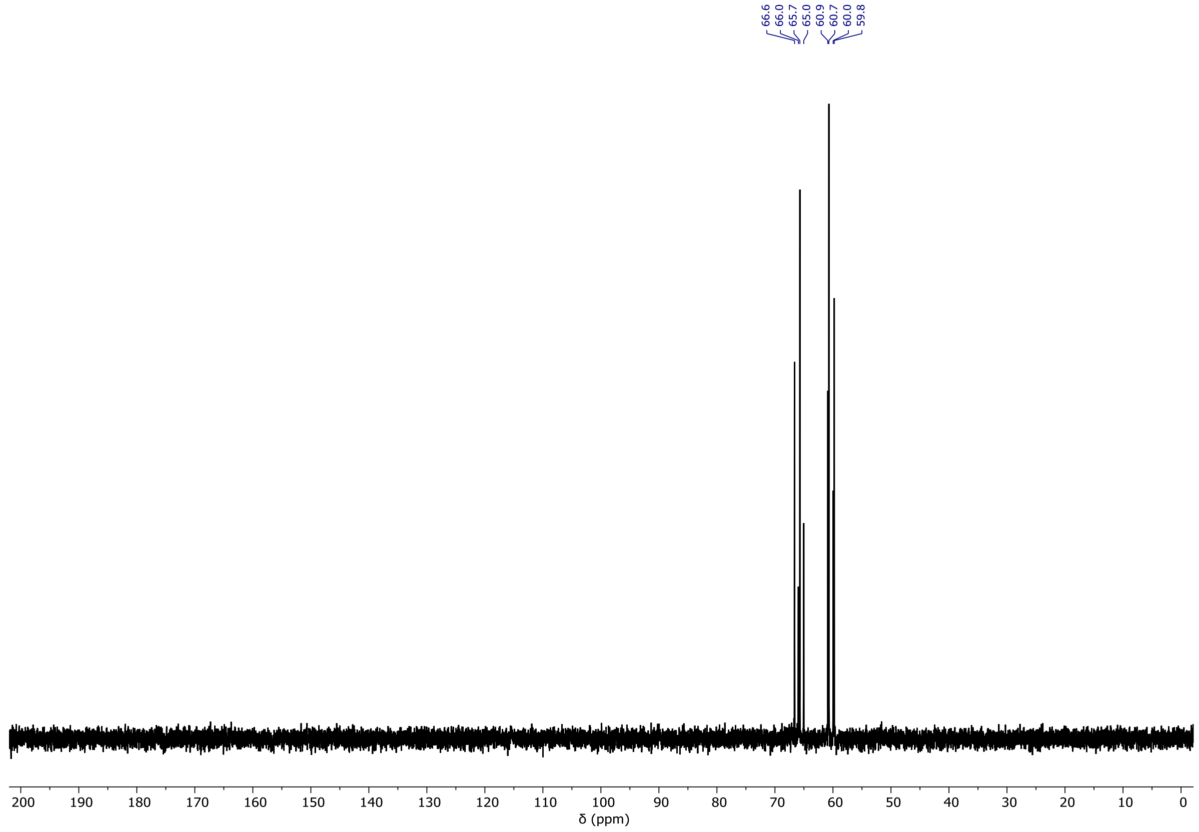

^{1}H NMR (600 MHz, THF-d_8, 20 °C) of Fe(κ[3]*PCP*-PCP-*i*Pr)(C(=O)$CH_2CH_2CH_3$)(CO)(CN*t*Bu) (**5**)

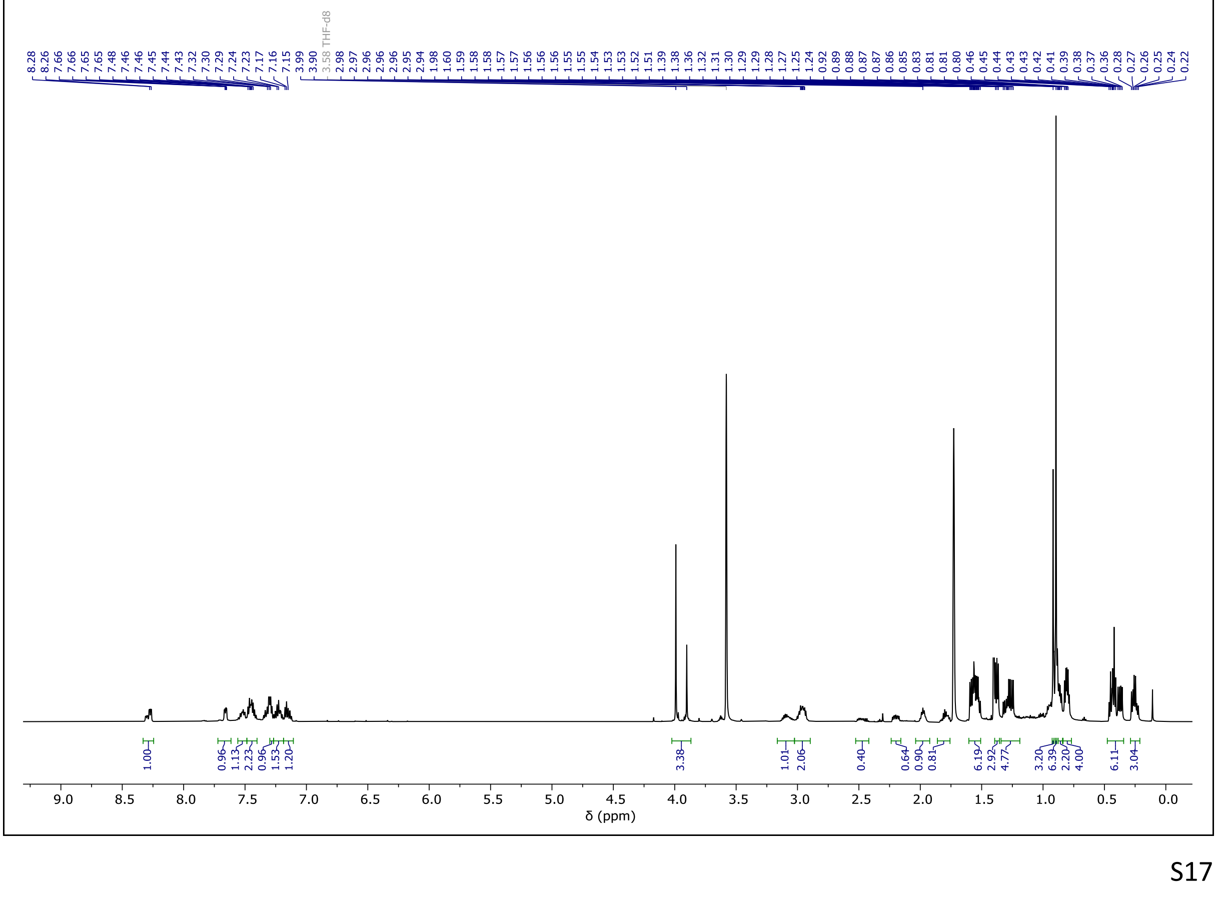

$^{13}C\{^1H\}$ NMR (151 MHz, THF-d$_8$, 20 °C) of Fe(κ^3*PCP*-PCP-*i*Pr)(C(=O)CH$_2$CH$_2$CH$_3$)(CO)(CN*t*Bu) (**5**)

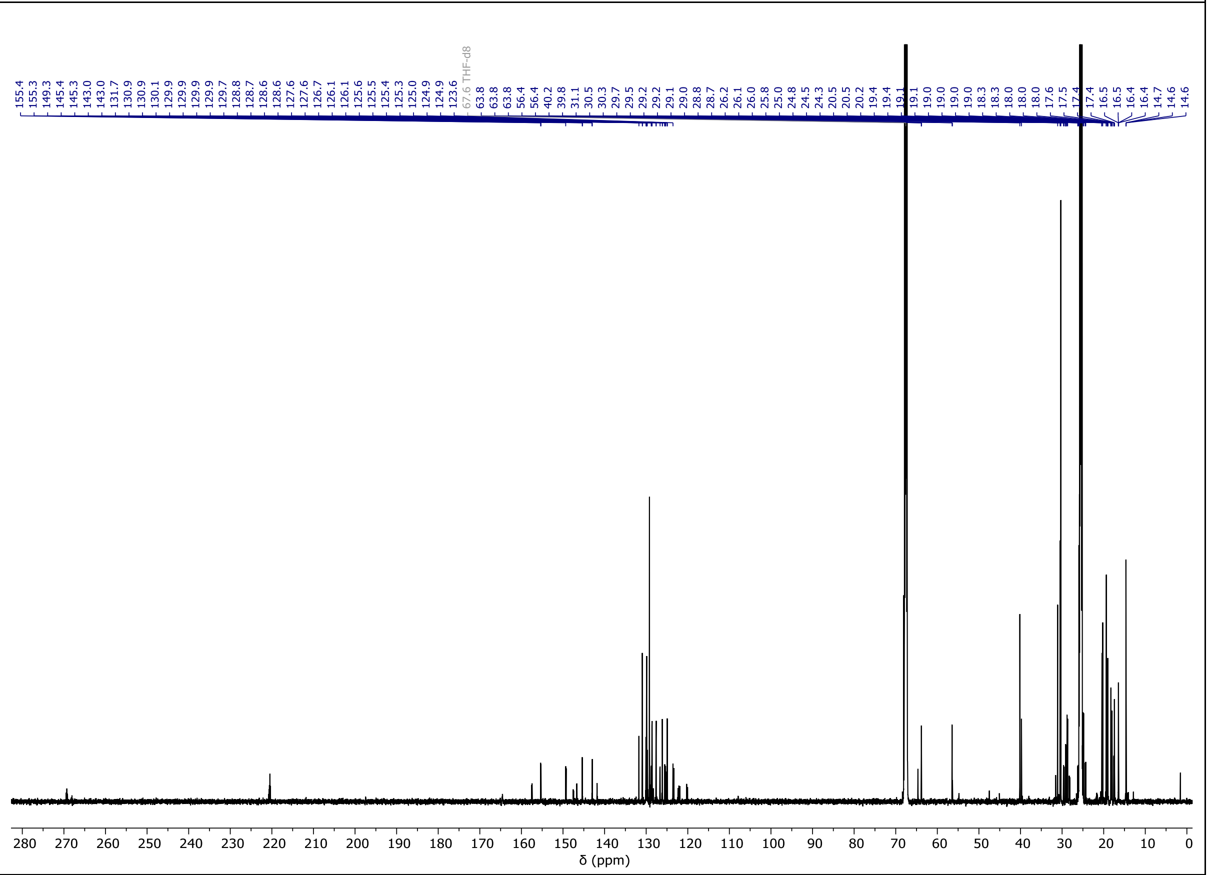

$^{31}P\{^1H\}$ NMR (243 MHz, THF-d$_8$, 20 °C) of Fe(κ^3*PCP*-PCP-*i*Pr)(C(=O)CH$_2$CH$_2$CH$_3$)(CO)(CN*t*Bu) (**5**)

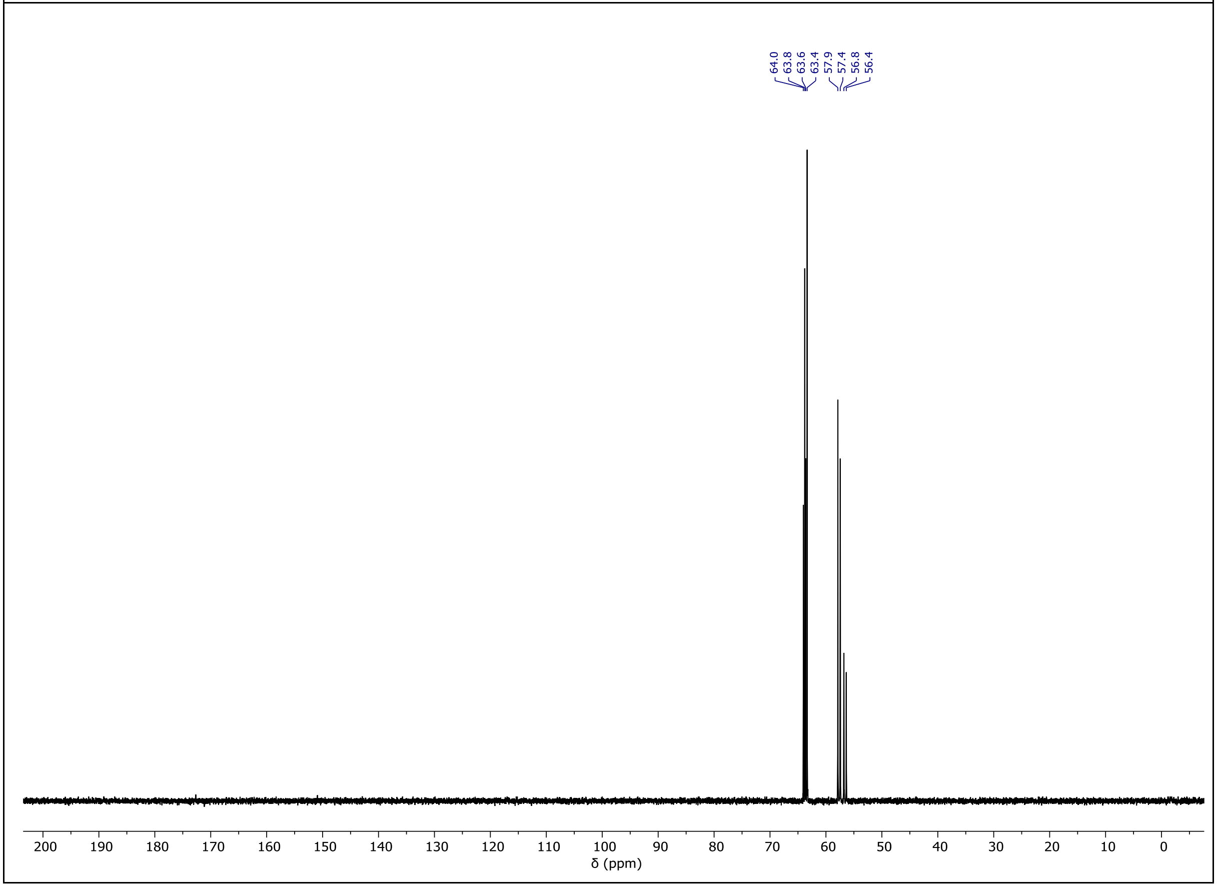

NMR spectra of substrate scope

^{1}H NMR (400 MHz, THF-d_8, 20 °C, ppm): Styrene (**A1**)

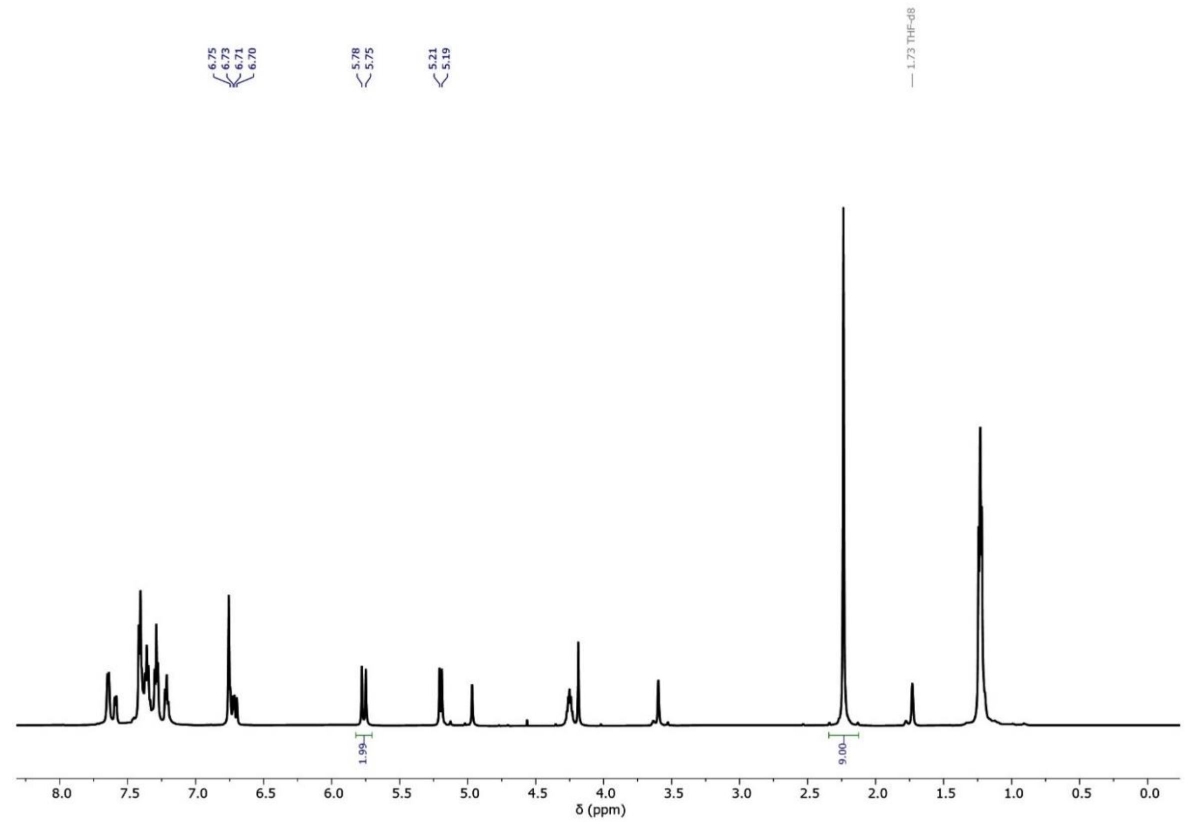

^{1}H NMR (600 MHz, THF-d_8, 20 °C, ppm): 4-Fluorostyrene (**A2**)

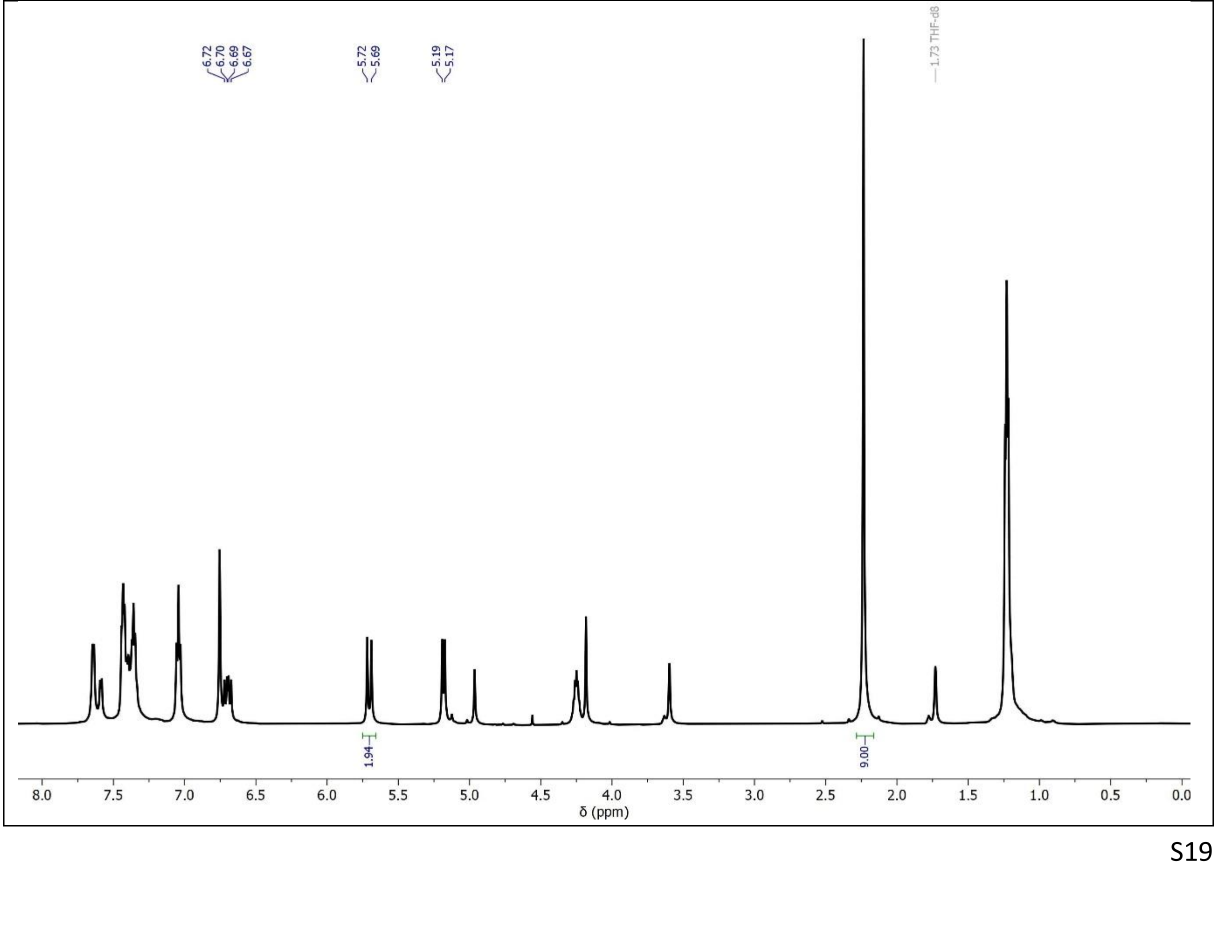

^{1}H NMR (400 MHz, THF-d_8, 20 °C, ppm): 4-Chlorostyrene (**A3**)

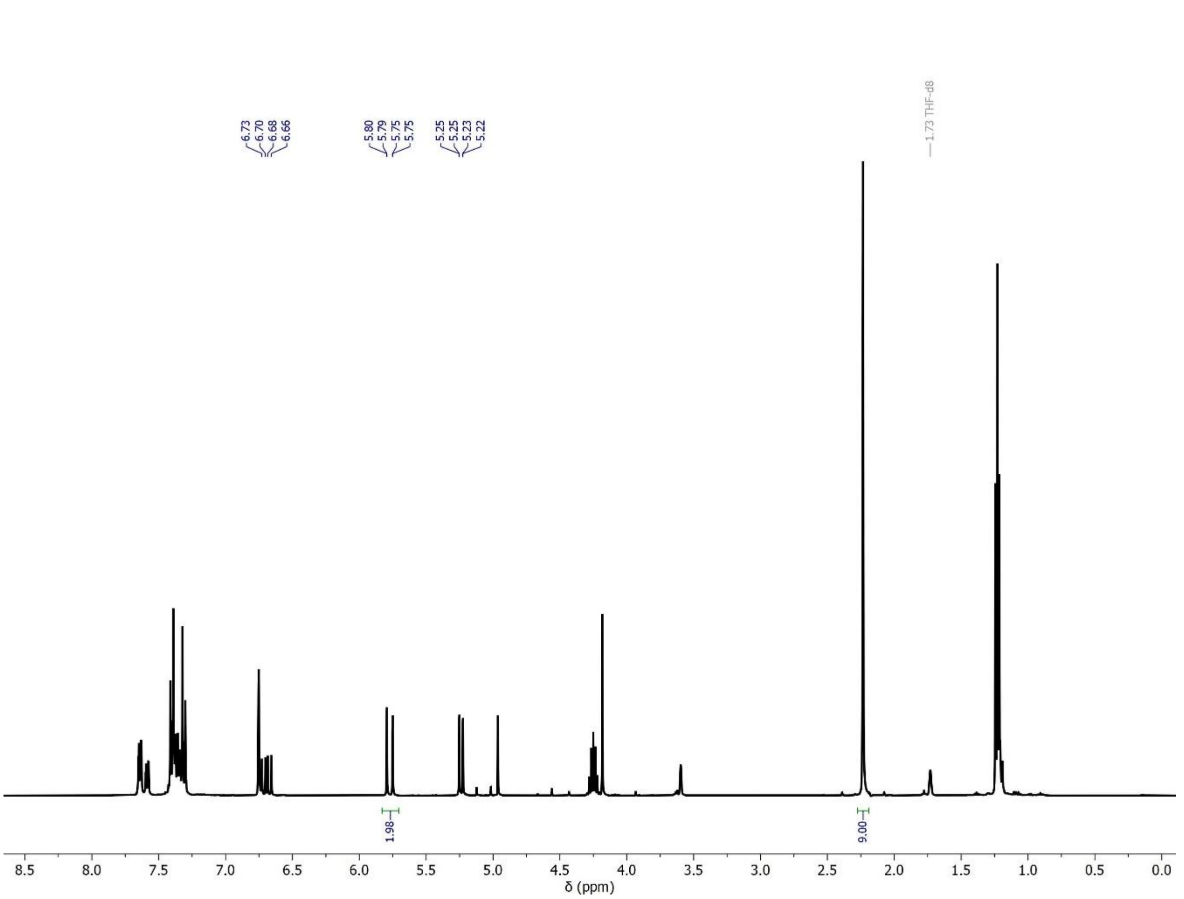

^{1}H NMR (600 MHz, THF-d_8, 20 °C, ppm): 4-Methylstyrene (**A4**)

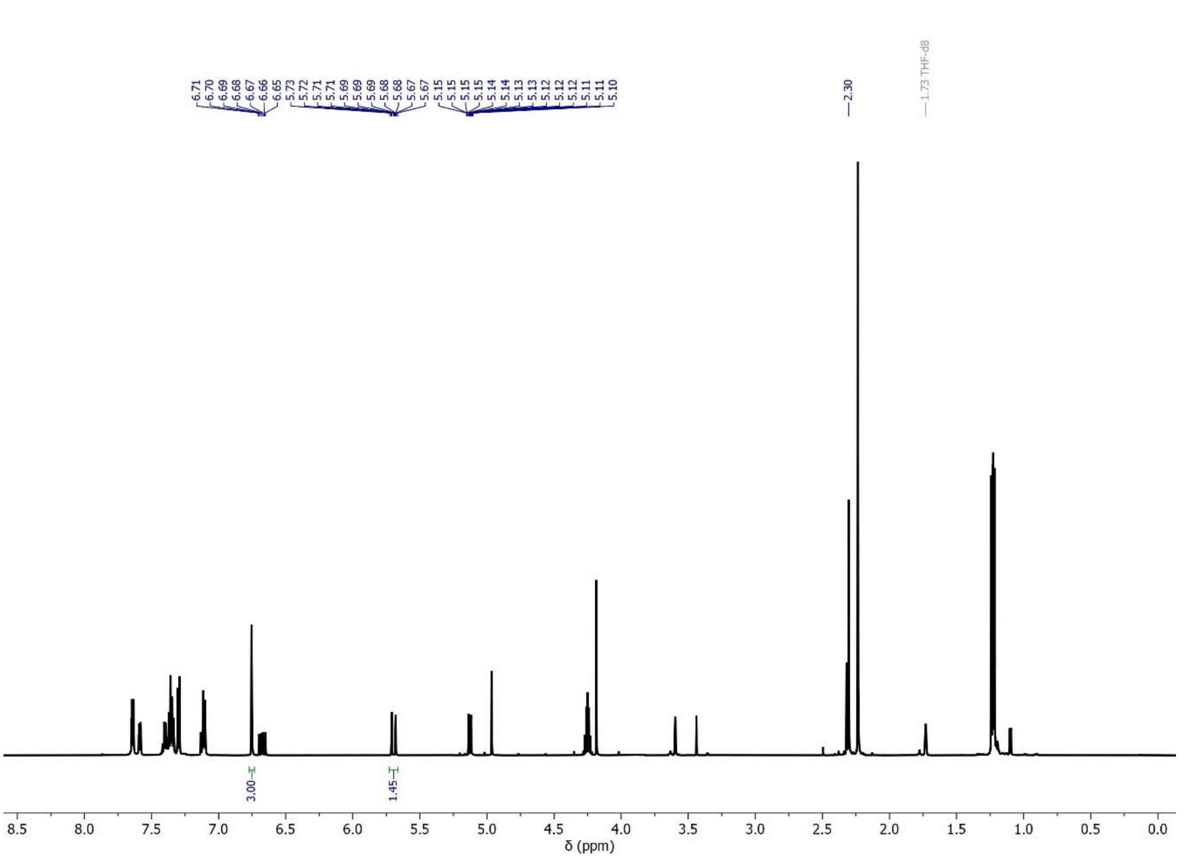

^{1}H NMR (600 MHz, THF-d_8, 20 °C, ppm): 4-(*tert*-Butyl)styrene (**A5**)

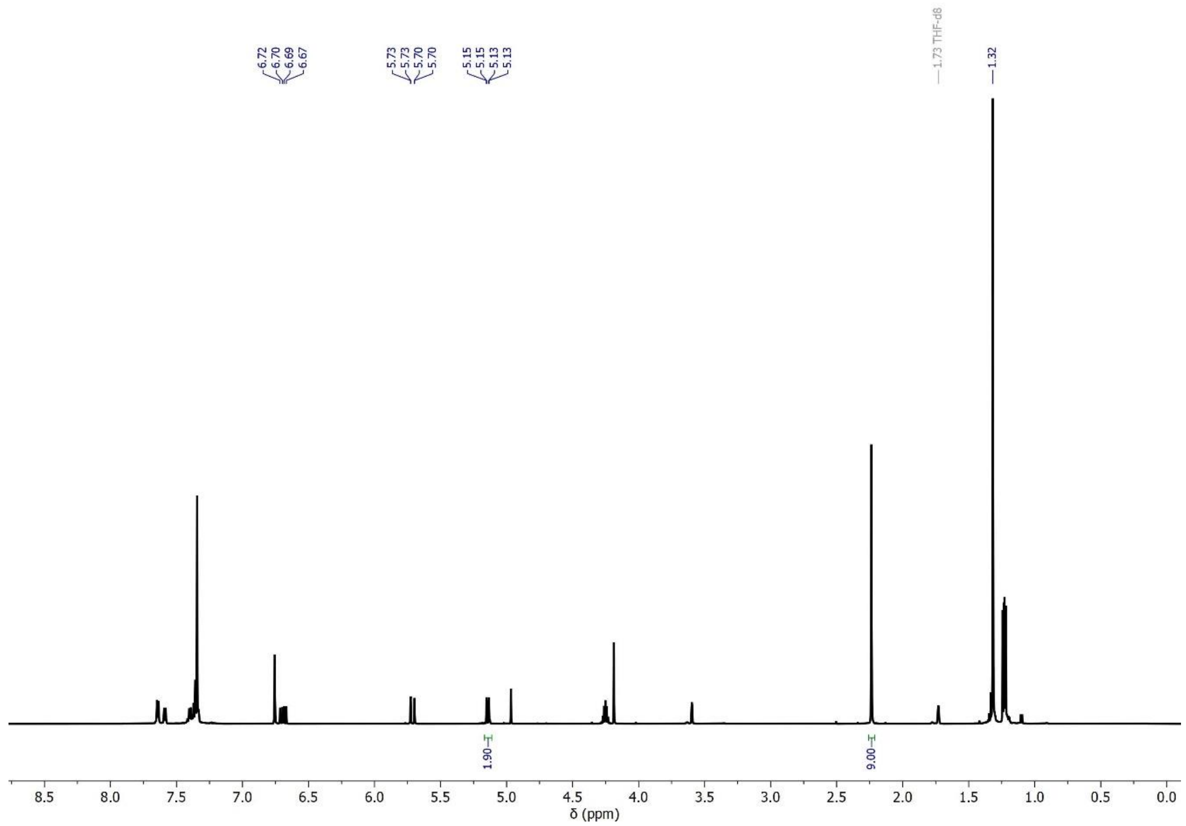

^{1}H NMR (600 MHz, THF-d_8, 20 °C, ppm): 4-Vinylanisole (**A6**)

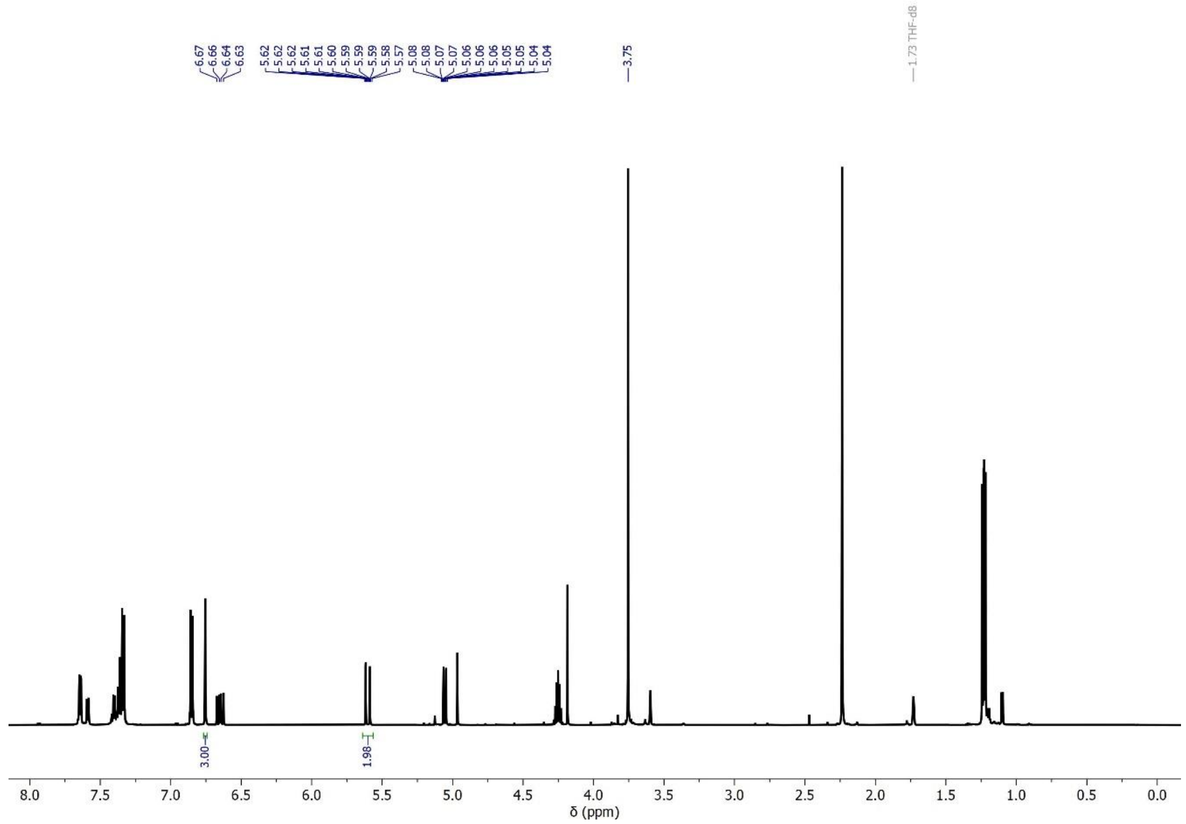

^{1}H NMR (600 MHz, THF-d_8, 20 °C, ppm): 4-Vinylbenzonitrile (**A7**)

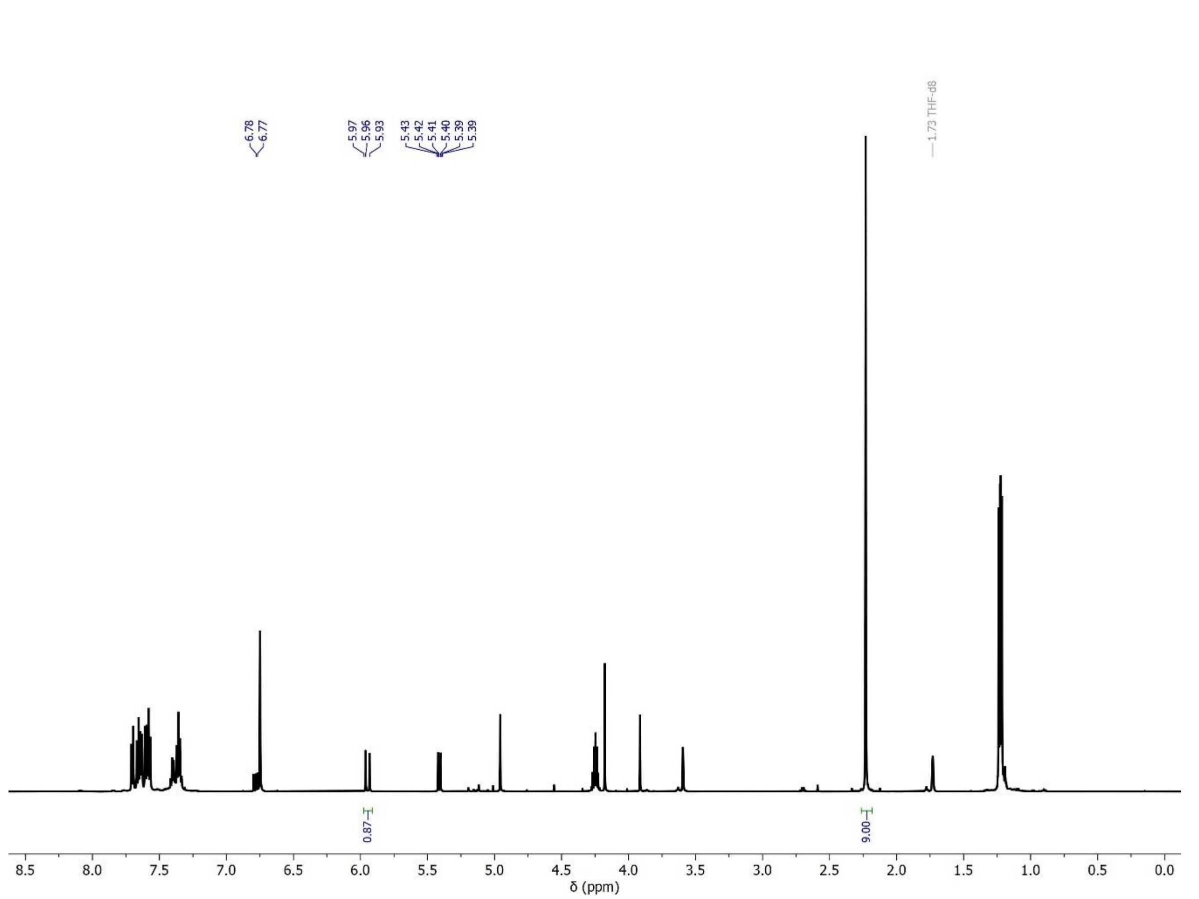

^{1}H NMR (600 MHz, THF-d_8, 20 °C, ppm): 4-Nitrostyrene (**A8**)

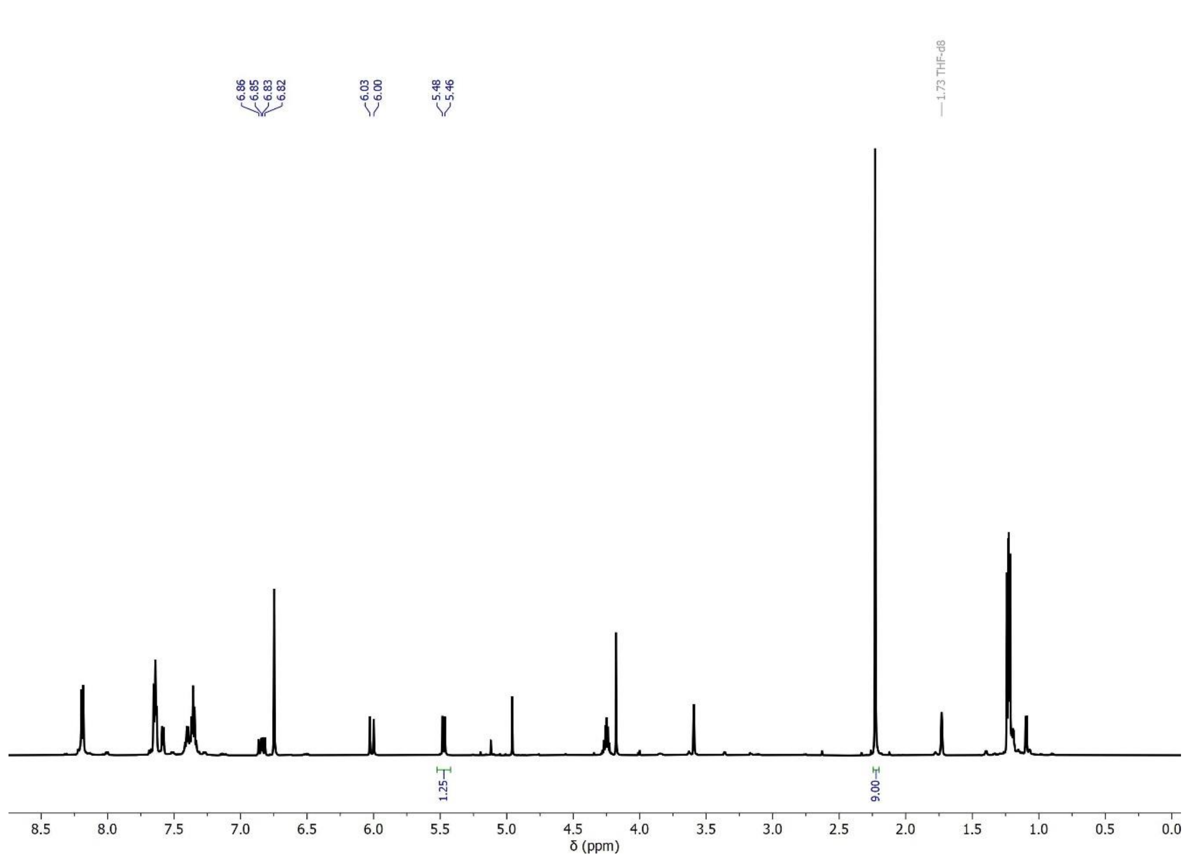

^{1}H NMR (600 MHz, THF-d$_8$, 20 °C, ppm): 4-Vinylacetophenone (**A9**)

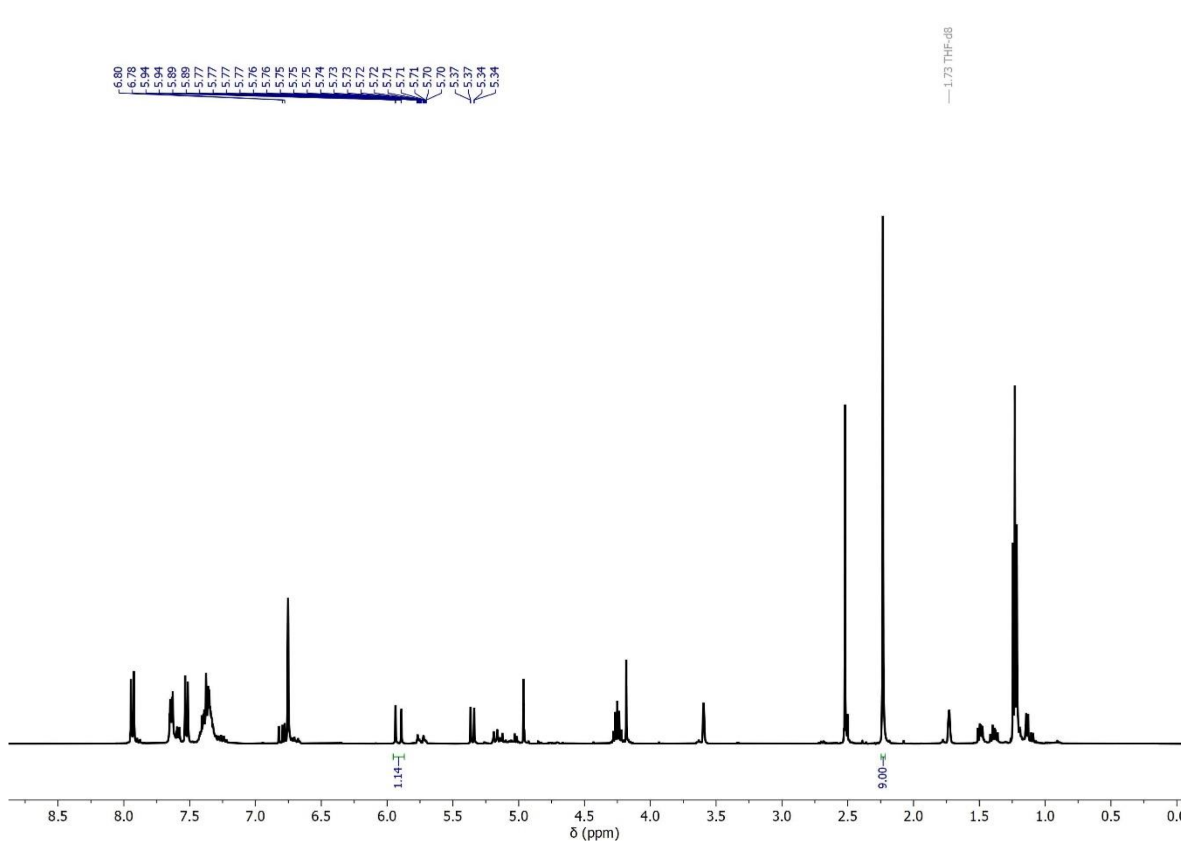

^{1}H NMR (600 MHz, THF-d$_8$, 20 °C, ppm): 3-Methylstyrene (**A10**)

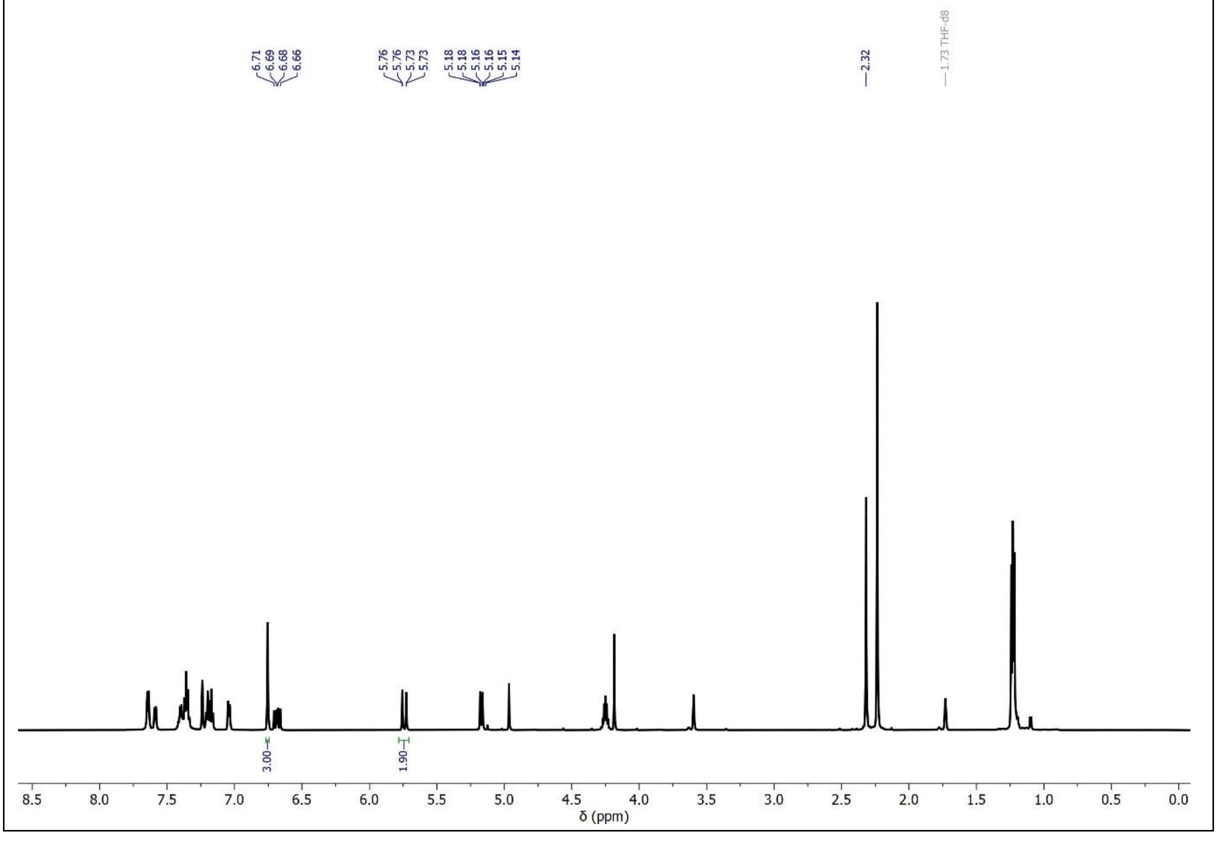

^{1}H NMR (400 MHz, THF-d_8, 20 °C, ppm): 3-Vinylaniline (**A12**)

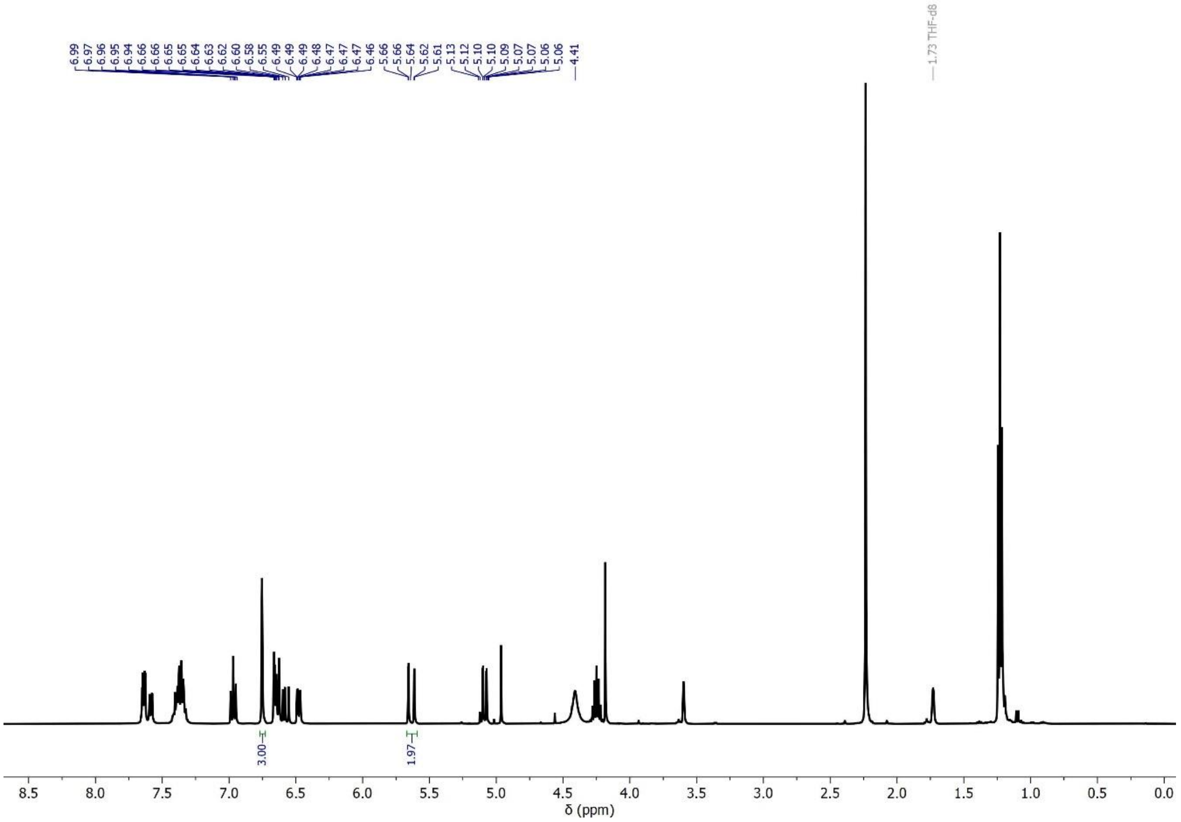

^{1}H NMR (600 MHz, THF-d_8, 20 °C, ppm): 2-Vinylpyridine (**A13**)

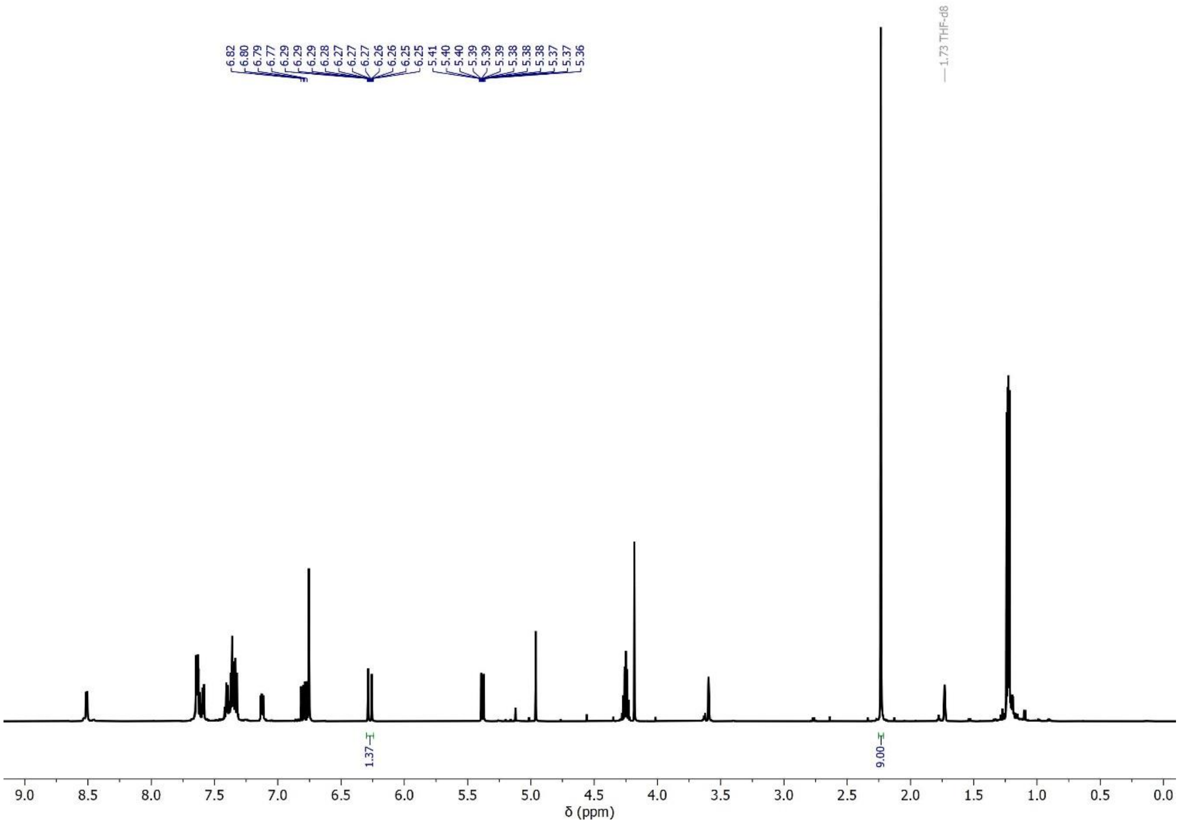

^{1}H NMR (600 MHz, THF-d_8, 20 °C, ppm): 3-Vinylthiophene (**A14**)

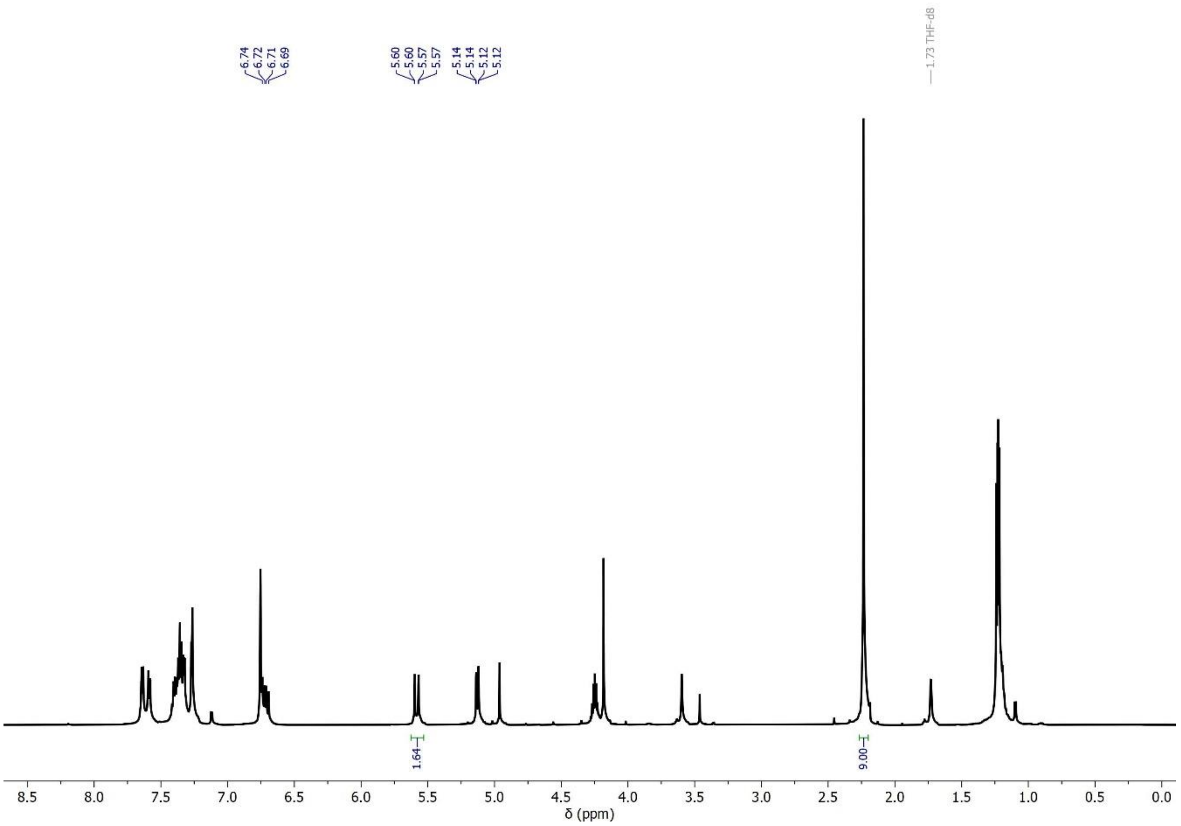

^{1}H NMR (600 MHz, THF-d_8, 20 °C, ppm): 3-Phenyl-1-propene (**A15**)

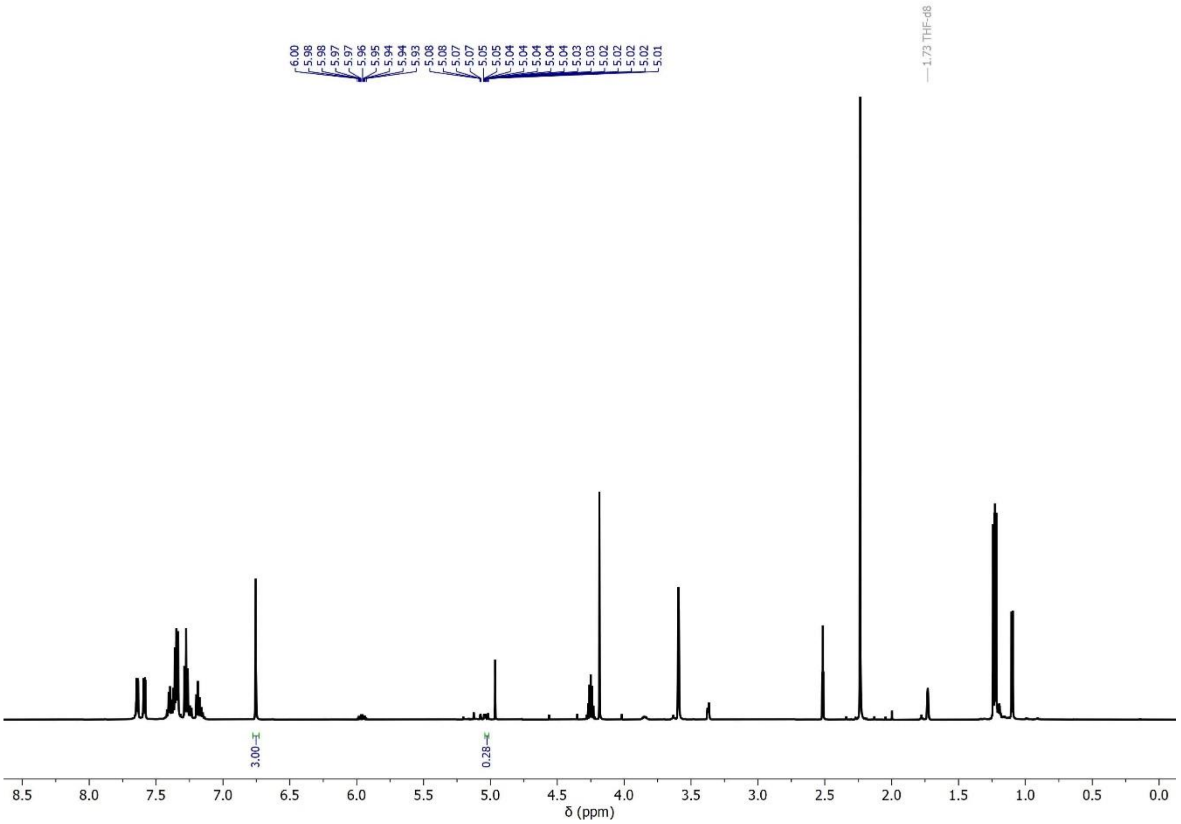

^{1}H NMR (400 MHz, THF-d_8, 20 °C, ppm): 1-Octene (**A16**)

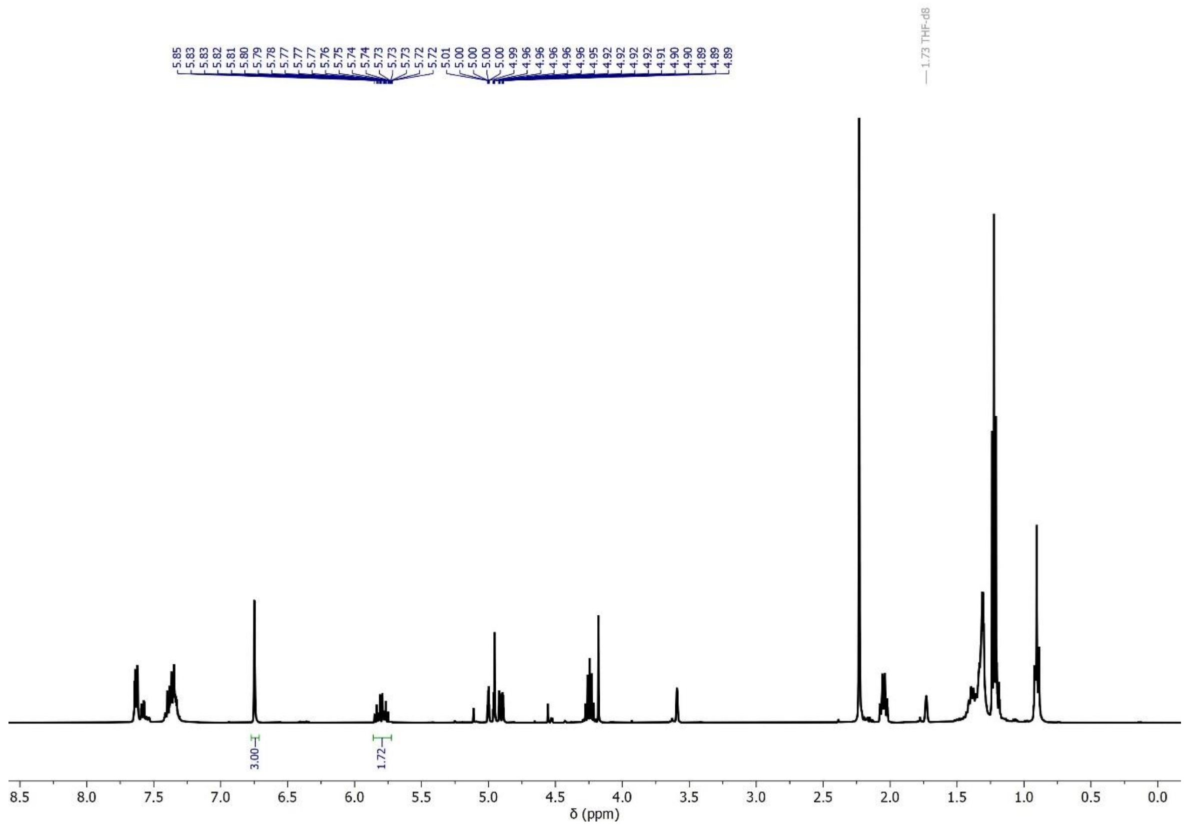

^{1}H NMR (600 MHz, THF-d_8, 20 °C, ppm): 6-Chloro-1-hexene (**A17**)

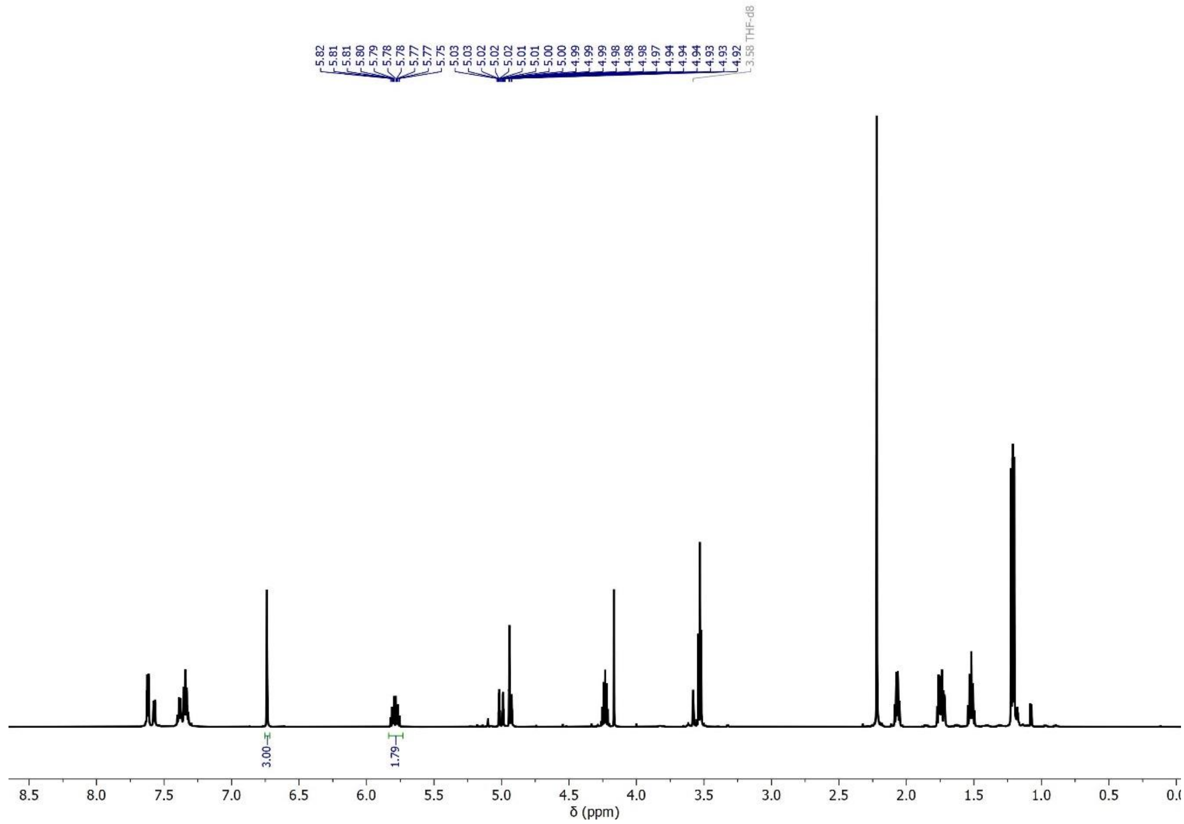

^{1}H NMR (600 MHz, THF-d_8, 20 °C, ppm): 1-Vinyl-1-cyclohexene (**A18**)

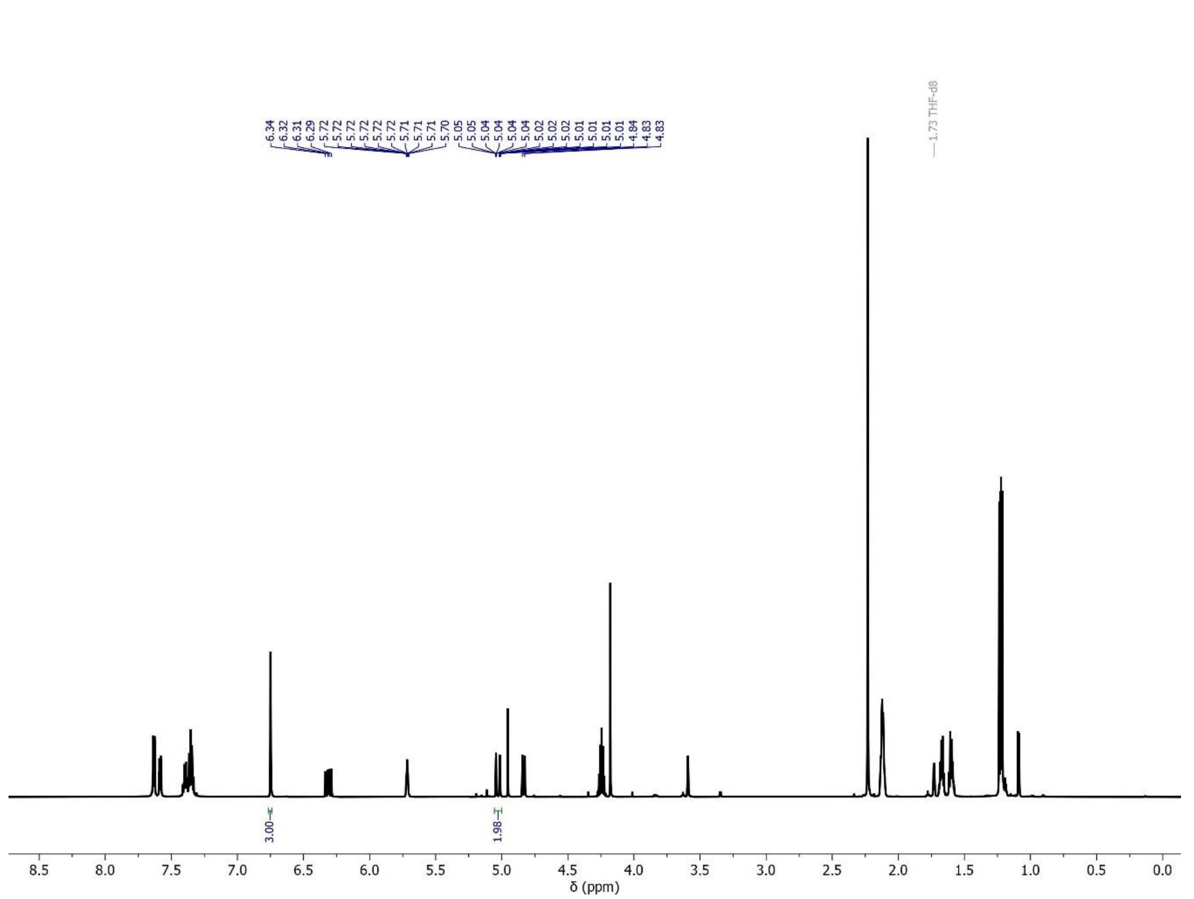

^{1}H NMR (600 MHz, THF-d_8, 20 °C, ppm): (*Z*)-Stilbene (**A19**)

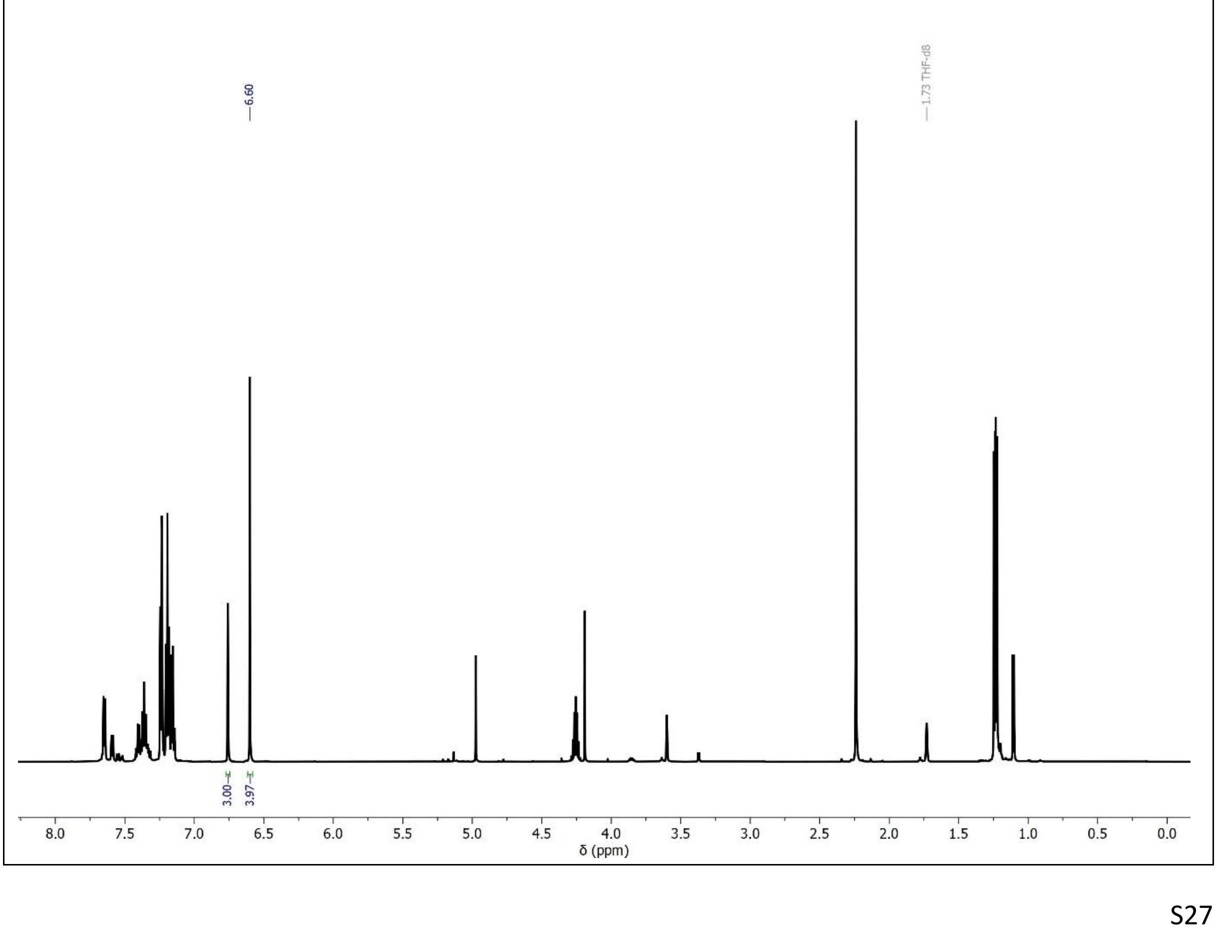

^{1}H NMR (600 MHz, THF-d$_8$, 20 °C, ppm): (*Z*)-1-Methylstyrene (**A20**)

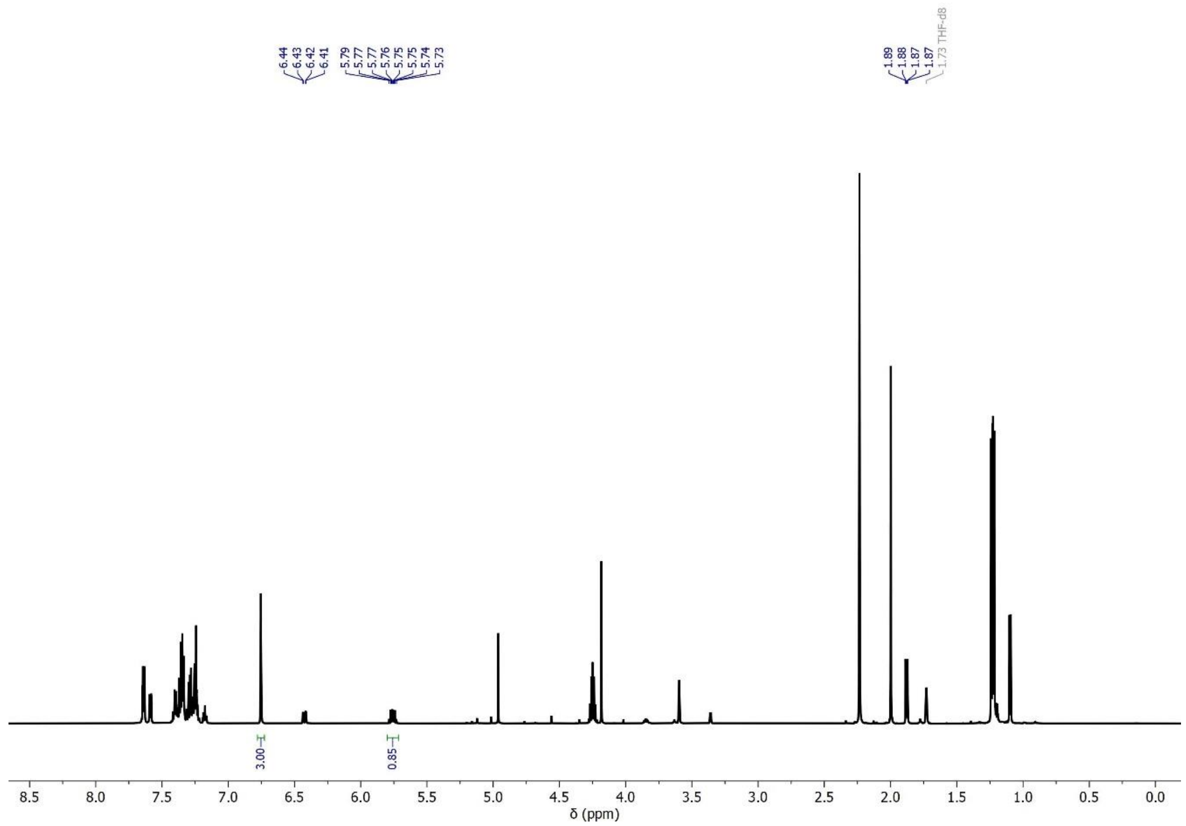

^{1}H NMR (600 MHz, THF-d$_8$, 20 °C, ppm): (*Z*)-β-(Trimethylsilyl)styrene (**A21**)

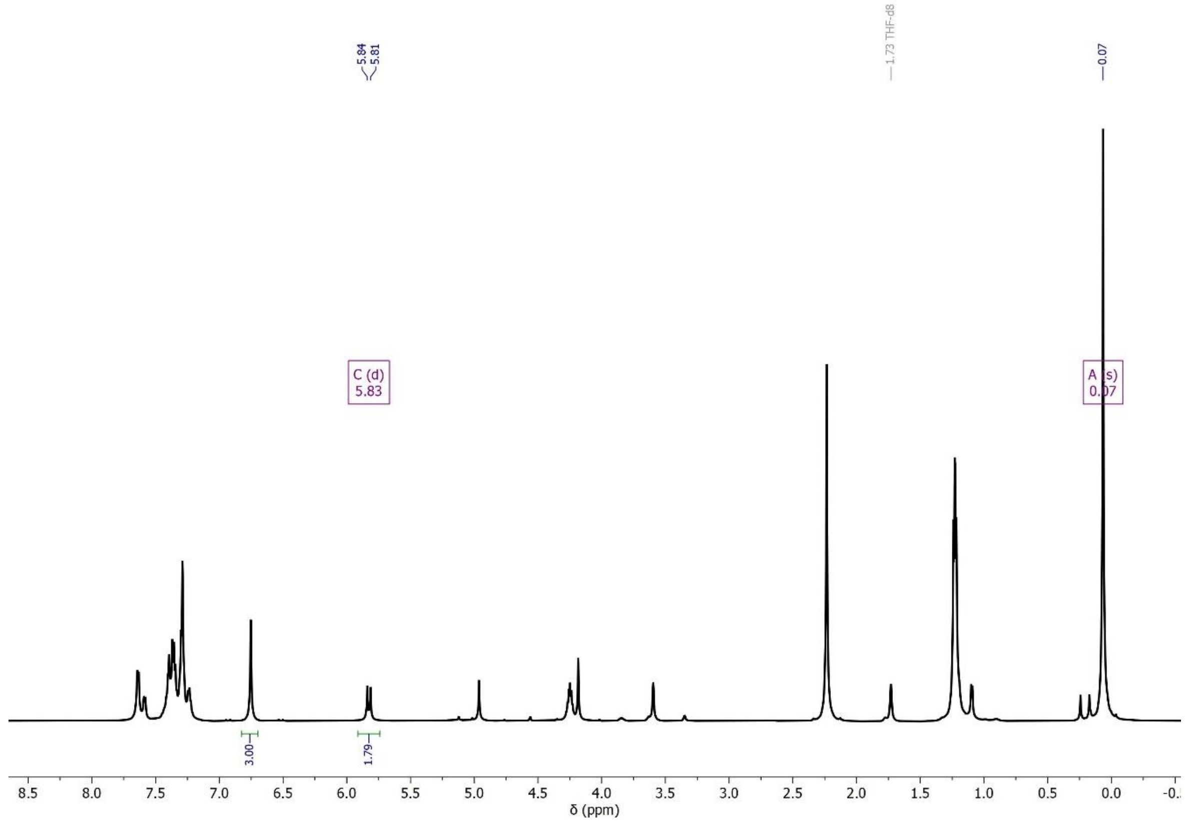

^{1}H NMR (600 MHz, THF-d_8, 20 °C, ppm): (*Z*)-4-Octyne (**A22**)

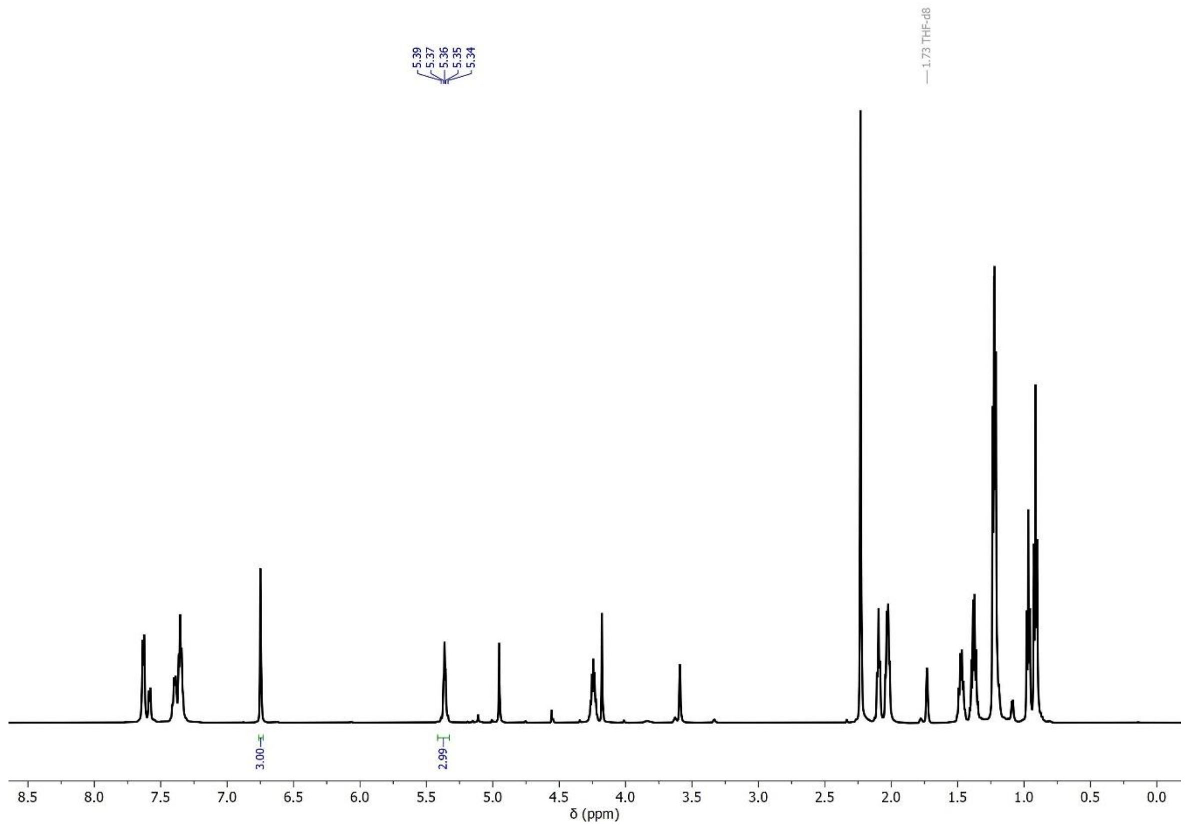

^{1}H NMR (600 MHz, THF-d_8, 20 °C, ppm): (Z)-1-(Trimethylsilyl)octene (**A23**)

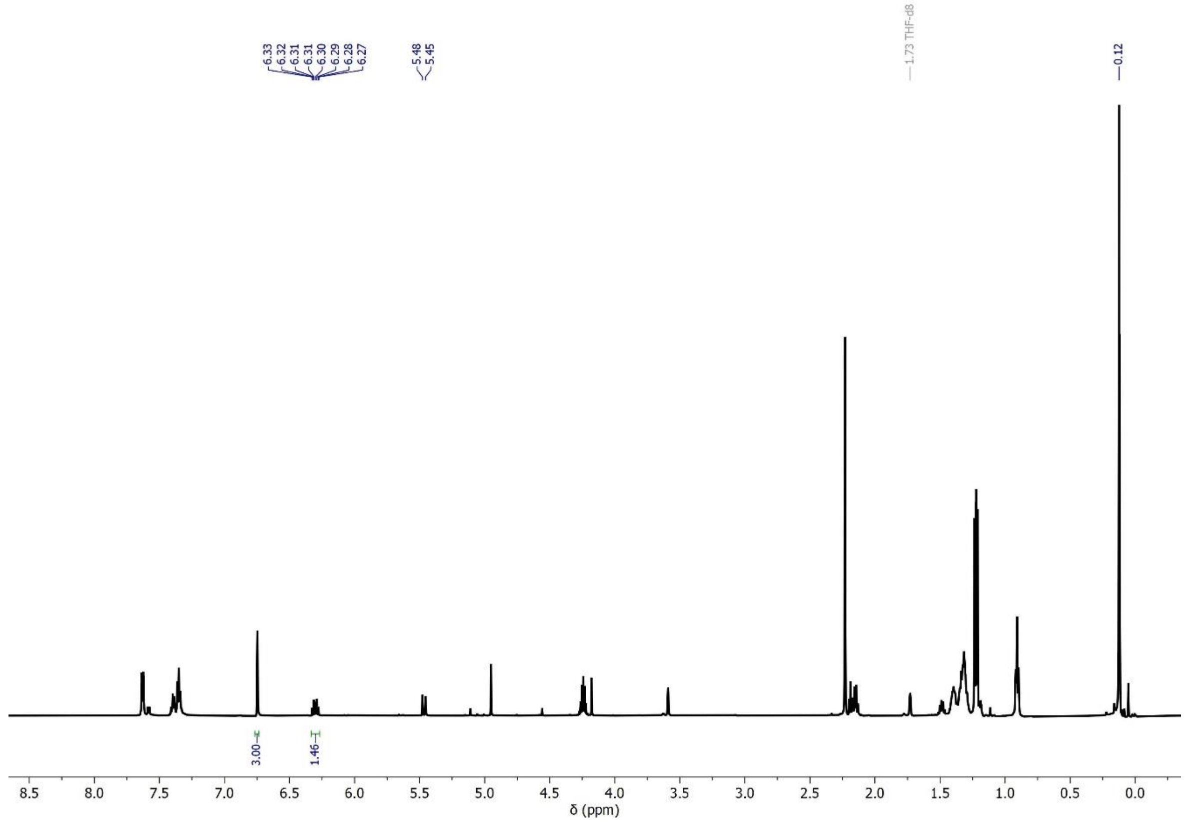

^{2}H NMR spectra

Phenylacetylene + $PhSiH_3$ + *i*PrOH-d_1

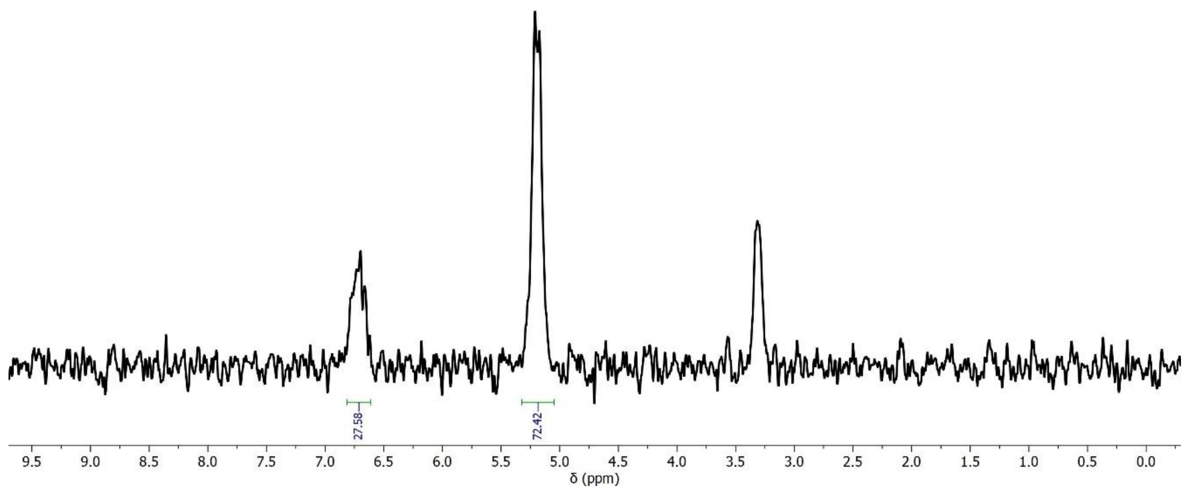

Phenylacetylene-d_1 + $PhSiH_3$ + *i*PrOH

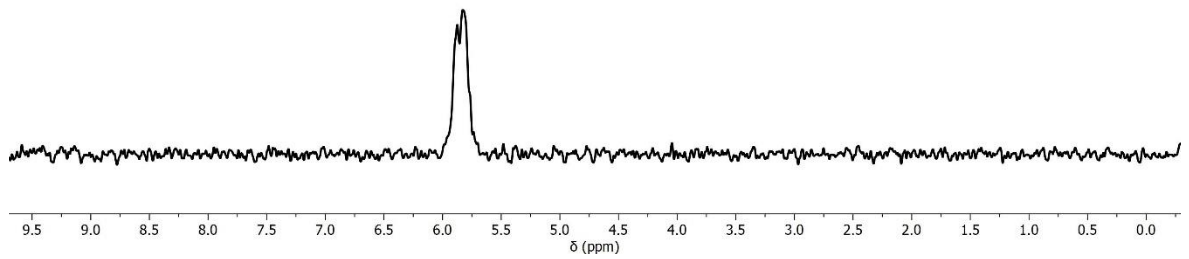

Phenylacetylene + $PhSiH_3$ + *i*PrOH-d_8

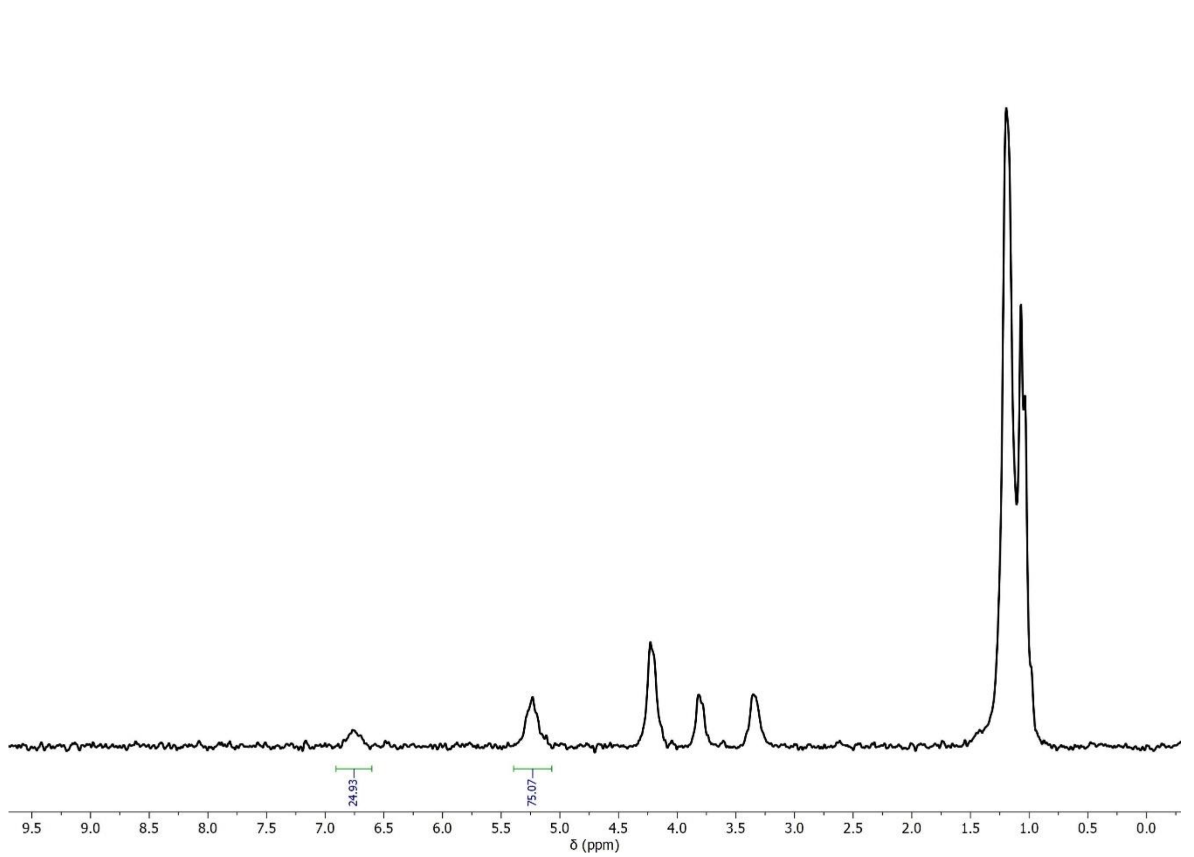

Phenylacetylene-d_1 + $PhSiH_3$ + *i*PrOH-d_1

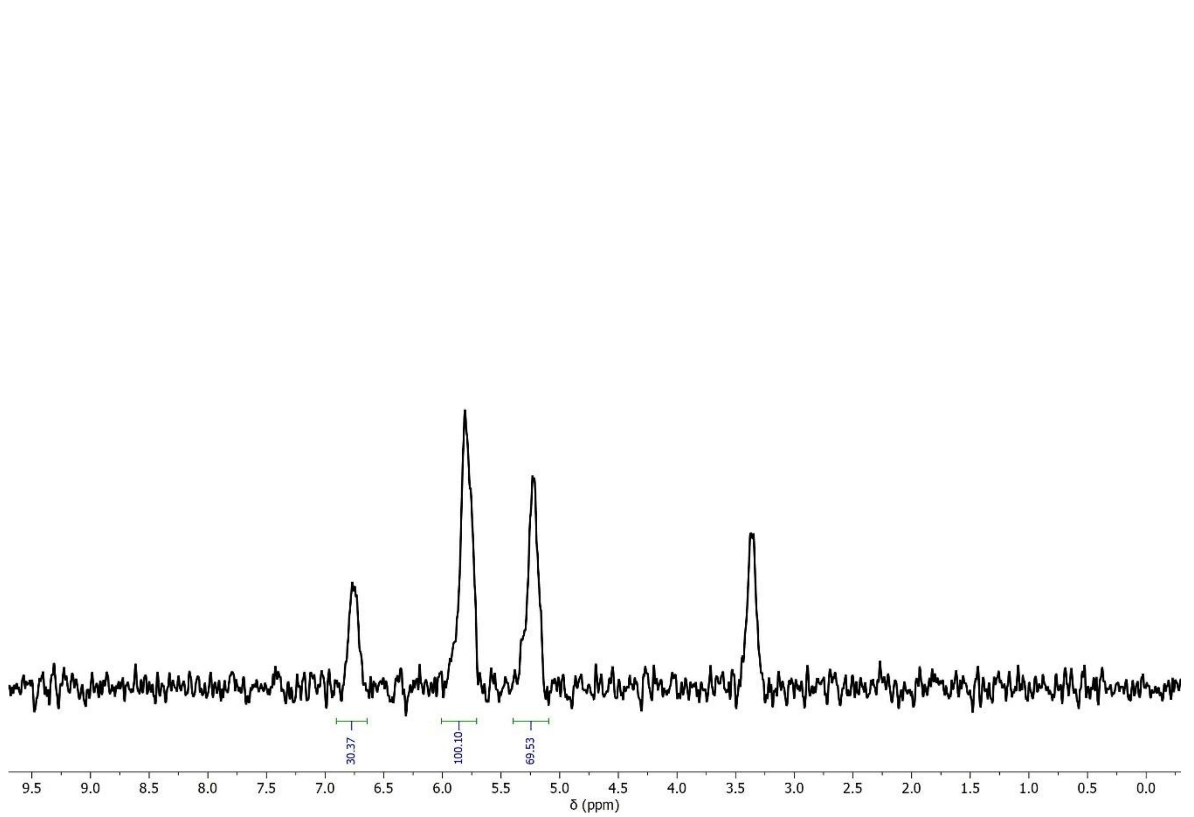

Phenylacetylene-d_1 + $PhSiH_3$ + *i*PrOH-d_8

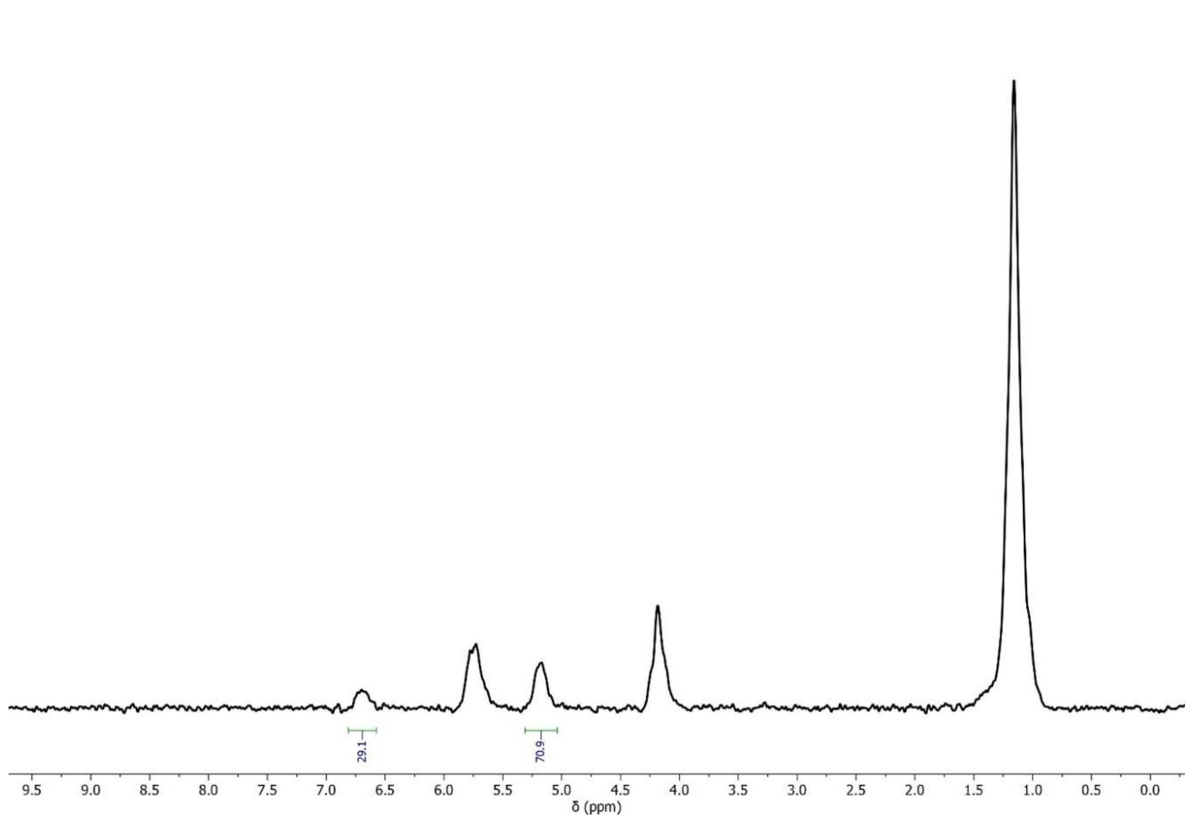

References

(1) Perrin, D. D.; Armarego, W. L. F. *Purification of Laboratory Chemicals*, 3rd ed.; Pergamon: New York, **1988**.

(2) Schratzberger, H.; Liebminger, L. A.; Stöger, B.; Veiros, L. F.; Kirchner, K. Base Metal Complexes bearing Pyrazole-derived PCP Ligands. *Dalton Trans.* **2023**, *52*, in press (DOI: 10.1039/D3DT02111D).

(3) Bruker computer programs: APEX3, SAINT and SADABS (Bruker AXS Inc., Madison, WI, 2020).

(4) G. M. Sheldrick, *Acta Crystallogr.* 2015, A**71**, 3–8.

(5) G. M. Sheldrick, *Acta Crystallogr.* 2015**,** C**71**, 3–8

(6) C. F. Macrae, P. R. Edgington, P. McCabe, E. Pidcock, G. P. Shields, R. Taylor, M. Towler and J. van de Streek, *J. Appl. Cryst.* 2006, **39**, 453–457.

(7) Gaussian 09, Revision **A.01**, Frisch, M. J.; Trucks, G. W.; Schlegel, H. B.; Scuseria, G. E.; Robb, M. A.; Cheeseman, J. R.; Scalmani, G.; Barone, V.; Mennucci, B.; Petersson, G. A.; Nakatsuji, H.; Caricato, M.; Li, X.; Hratchian, H. P.; Izmaylov, A. F.; Bloino, J.; Zheng, G.; Sonnenberg, J. L.; Hada, M.; Ehara, M.; Toyota, K.; Fukuda, R.; Hasegawa, J.; Ishida, M.; Nakajima, T.; Honda, Y.; Kitao, O.; Nakai, H.; Vreven, T.; Montgomery, Jr., J. A.; Peralta, J. E.; Ogliaro, F.; Bearpark, M.; Heyd, J. J.; Brothers, E.; Kudin, K. N.; Staroverov, V. N.; Kobayashi, R.; Normand, J.; Raghavachari, K.; Rendell, A.; Burant, J. C.; Iyengar, S. S.; Tomasi, J.; Cossi, M.; Rega, N.; Millam, J. M.; Klene, M.; Knox, J. E.; Cross, J. B.; Bakken, V.; Adamo, C.; Jaramillo, J.; Gomperts, R.; Stratmann, R. E.; Yazyev, O.; Austin, A. J.; Cammi, R.; Pomelli, C.; Ochterski, J. W.; Martin, R. L.; Morokuma, K.; Zakrzewski, V. G.; Voth, G. A.; Salvador, P.; Dannenberg, J. J.; Dapprich, S.; Daniels, A. D.; Farkas, Ö.; Foresman, J. B.; Ortiz, J. V.; Cioslowski, J.; Fox, D. J. Gaussian, Inc., Wallingford CT, 2009.

(8) Hehre, W. J., Radom, L., Schleyer, P. v.R. & Pople, J. A. *Ab Initio Molecular Orbital Theory*, John Wiley & Sons, NY, 1986.

(9) Parr, R. G.; Yang, W. Density Functional Theory of Atoms and Molecules; Oxford University Press: New York, 1989.

(10) (a) Perdew, J. P.; Burke, K.; Ernzerhof, M. Generalized Gradient Approximation Made Simple *Phys. Rev. Lett.* **1996**, *77*, 3865-3868. (b) Perdew, J. P.; Burke, K.; Ernzerhof, M. Generalized Gradient Approximation Made Simple *Phys. Rev. Lett.* **1997**, *78*, 1396-1396. (c) Perdew, J. P. Density-functional approximation for the correlation energy of the inhomogeneous electron gas *Phys. Rev. B* **1986**, *33*, 8822-8824.

(11) (a) Haeusermann, U.; Dolg, M.; Stoll, H.; Preuss, H.; Schwerdtfeger, P.; Pitzer, R. M. Accuracy of energy-adjusted quasirelativistic ab initio pseudopotentials *Mol. Phys.* **1993**, *78*, 1211-1224. (b) Kuechle, W.; Dolg, M.; Stoll, H.; Preuss, H. Energy-adjusted pseudopotentials for the actinides. Parameter sets and test calculations for thorium and thorium monoxide *J. Chem. Phys.* **1994**, *100*, 7535-7542. (c) Leininger, T.; Nicklass, A.; Stoll, H.; Dolg, M.; Schwerdtfeger, P. The accuracy of the pseudopotential approximation. II. A comparison of various core sizes for indium pseudopotentials in calculations for spectroscopic constants of InH, InF, and InCl *J. Chem. Phys.* **1996**, *105*, 1052-1059.

(12) (a) Ditchfield, R.; Hehre, W. J.; Pople, J. A. Self-Consistent Molecular-Orbital Methods. IX. An Extended Gaussian-Type Basis for Molecular-Orbital Studies of Organic Molecules *J. Chem. Phys.* **1971**, *54*, 724-728. (b) Hehre, W. J.; Ditchfield, R.; Pople, J. A. Self-Consistent Molecular Orbital Methods. 12. Further extensions of Gaussian-type basis sets for use in molecular-orbital studies of organic-molecules *J. Chem. Phys.* **1972**, *56*, 2257-2261. (c) Hariharan, P. C.; Pople, J. A. Accuracy

of AH equilibrium geometries by single determinant molecular-orbital theory *Mol. Phys.* **1974**, *27*, 209-214. d) Gordon, M. S. The isomers of silacyclopropane *Chem. Phys. Lett.* **1980**, *76*, 163-168. e) Hariharan, P. C.; Pople, J. A. Influence of polarization functions on molecular-orbital hydrogenation energies *Theor. Chim. Acta* **1973**, *28*, 213-222.

(13) (a) Peng, C.; Ayala, P. Y.; Schlegel, H. B.; Frisch, M. J. Using redundant internal coordinates to optimize equilibrium geometries and transition states *J. Comp. Chem.* **1996**, *17*, 49-56. b) Peng, C.; Schlegel, H. B. Combining Synchronous Transit and Quasi-Newton Methods for Finding Transition States *Israel J. Chem.* **1993**, *33*, 449-454.

(14) (a) McClean, A. D.; Chandler, G. S. Contracted Gaussian basis sets for molecular calculations. I. Second row atoms, *Z*=11-18 *J. Chem. Phys.* **1980**, *72*, 5639-5648. (b) Krishnan, R.; Binkley, J. S.; Seeger, R.; Pople, J. A. Self-consistent molecular orbital methods. XX. A basis set for correlated wave functions *J. Chem. Phys.* **1980**, *72*, 650-654. (c) Wachters, A. J. H. Gaussian Basis Set for Molecular Wavefunctions Containing Third-Row Atoms *J. Chem. Phys.* **1970**, *52*, 1033-1036. (d) Hay, P. J. Gaussian basis sets for molecular calculations - representation of 3D orbitals in transition-metal atoms *J. Chem. Phys.* **1977**, *66*, 4377-4384. (e) Raghavachari, K.; Trucks, G. W. Highly correlated systems: Excitation energies of first row transition metals Sc-Cu *J. Chem. Phys.* **1989**, *91*, 1062-1065. (f) Binning Jr., R. C.; Curtiss, L. A. Compact contracted basis-sets for 3rd-row atoms - Ga-Kr *J. Comp. Chem.* **1990**, *11*, 1206-1216. (g) McGrath, M. P.; Radom, L. Extension of Gaussian-1 (G1) theory to bromine-containing molecules *J. Chem. Phys.* **1991**, *94*, 511-516. (h) Curtiss, L. A.; McGrath, M. P.; Blaudeau, J.-P.; Davis, N. E.; Binning Jr., R. C.; Radom, L. Extension of Gaussian-2 theory to molecules containing third-row atoms Ga-Kr *J. Chem. Phys.*, **1995**, *103*, 6104-6113. (i) Clark, T.; Chandrasekhar, J.; Spitznagel, G. W.; Schleyer, P. v. R. Efficient diffuse function-augmented basis-sets for anion calculations. 3. The 3-21+G basis set for 1st-row elements, Li-F *J. Comp. Chem.* **1983**, *4*, 294-301. (j) Frisch, M. J.; Pople, J. A.; Binkley, J. S. Self-Consistent Molecular Orbital Methods. 25. Supplementary Functions for Gaussian Basis Sets *J. Chem. Phys.* **1984**, *80*, 3265-3269.

(15) (a) Cancès, M. T.; Mennucci, B.; Tomasi, J. A new integral equation formalism for the polarizable continuum model: Theoretical background and applications to isotropic and anistropic dielectrics *J. Chem. Phys.* **1997**, *107*, 3032-3041. (b) Cossi, M.; Barone, V.; Mennucci, B.; Tomasi, J. Ab initio study of ionic solutions by a polarizable continuum dielectric model *Chem. Phys. Lett.* **1998**, *286*, 253-260. (c) Mennucci, B.; Tomasi, J. Continuum solvation models: A new approach to the problem of solute's charge distribution and cavity boundaries *J. Chem. Phys.* **1997**, *106*, 5151-5158. (d) Tomasi, J.; Mennucci, B.; Cammi, R. Quantum mechanical continuum solvation models *Chem. Rev.* **2005**, *105*, 2999-3094.

(16) Marenich, A. V.; Cramer, C. J.; Truhlar, D. G. Universal solvation model based on solute electron density and a continuum model of the solvent defined by the bulk dielectric constant and atomic surface tensions *J. Phys. Chem. B*, **2009**, *113*, 6378-6396.

(17) Grimme, S.; Antony, J.; Ehrlich, S.; Krieg, H. A consistent and accurate ab initio parameterization of density functional dispersion correction (DFT-D) for the 94 elements H-Pu *J. Chem. Phys.* **2010**, *132*, 154104.

(18) (a) Becke, A. D.; Johnson, E. R. A density-functional model of the dispersion interaction *J. Chem. Phys.* **2005**, *122*, 154101. (b) Johnson, E. R.; Becke, A. D. A post-Hartree-Fock model of intermolecular interactions *J. Chem. Phys.* **2005**, *123*, 24101. (c) Johnson, E. R.; Becke, A. D. A post-Hartree-Fock model of intermolecular interactions: Inclusion of higher-order corrections *J. Chem. Phys.* **2006**, *124*, 174104.

(19) Pandey, D. K.; Khaskin, E.; Pal, S.; Fayzullin, R. R.; Khusnutdinova, J. R. Efficient Fe-Catalyzed Terminal Alkyne Semihydrogenation by H_2: Selectivity Control via a Bulky PNP Pincer Ligand. *ACS Catal.* **2023**, *13*, 375-381.

(20) Espinal-Viguri, M.; Neale, S. E.; Coles, N. T.; Macgregor, S. A., Webster. R. L. Room Temperature Iron-Catalyzed Transfer Hydrogenation and Regioselective Deuteration of Carbon–Carbon Double Bonds. *J. Am. Chem. Soc.* **2019**, *141*, 572-582.

(21) Molander, G. A.; Brown, A. R. Suzuki−Miyaura Cross-Coupling Reactions of Potassium Vinyltrifluoroborate with Aryl and Heteroaryl Electrophiles**.** *J. Org. Chem.* **2006**, *71*, 9681-9686.

(22) Wienhöfer, G.; Westerhaus, F. A.; Jagadeesh, R. V.; Junge, K.; Junge, H.; Beller, M. Selective iron-catalyzed transfer hydrogenation of terminal alkynes. *Chem. Commun.* **2012**, *48*, 4827– 4829.

(23) Sorribes, I.; Liu, L.; Corma, A. Nanolayered Co-Mo-S Catalysts for the Chemoselective Hydrogenation of Nitroarenes. *ACS Catal.* **2017**, *7*, 2698-2708.

(24) Liang, S.; Hammond, G. B.; Xu, B. Supported gold nanoparticles catalyzed *cis*-selective semihydrogenation of alkynes using ammonium formate as the reductant. *Chem. Commun.* **2016**, *52*, 6013-6016.

(25) Bedford, R. B.; Brenner, P. B.; Carter, E.; Carvell, W. T.; Cogswell, P. M; Gallagher, T.; Harvey, J. N.; Murphy, D. M.; Neeve, E. C.; Nunn, J.; Pye, D. R. Expedient Iron-Catalyzed Coupling of Alkyl, Benzyl and Allyl Halides with Arylboronic Esters. *Chem. Eur. J.* **2014**, *20*, 7935-7938.

(26) John, A.; Dereli, B.; Ortuño, M. A. Johnson, H. E.; Hillmyer, M. A.; Cramer, C. J.; Tolman, W. B. Selective Decarbonylation of Fatty Acid Esters to Linear α-Olefins. *Organometallics* **2017**, *36*, 2956-2964.

(27) Mitsudome, T.; Urayama, T.; Yamazaki, K.; Maehara, Y.; Yamasaki, J.; Gohara, K.; Maeno, Z., Mizugaki, T.; Jitsukawa, K.; Kaneda, K. Design of Core-Pd/Shell-Ag Nanocomposite Catalyst for Selective Semihydrogenation of Alkynes. *ACS Catal.* **2016**, *6*, 666-670.

(28) Gorgas, N.; Brünig, J.; Stöger, B.; Vanicek, S.; Tilset, M.; Veiros, L. F.; Kirchner, K. Efficient Z-Selective Semihydrogenation of Internal Alkynes Catalyzed by Cationic Iron(II) Hydride Complexes. *J. Am. Chem. Soc.* **2019**, *141*, 17452– 17458.
